# A Novel Exploration Stage Approach to Improve Crayfish Optimization Algorithm: Solution to Real-World Engineering Design Problems

**DOI:** 10.3390/biomimetics10060411

**Published:** 2025-06-19

**Authors:** Harun Gezici

**Affiliations:** Electronics and Automation Department, Kırklareli University, 39010 Kırklareli, Turkey; harun.gezici@klu.edu.tr

**Keywords:** crayfish optimization, engineering design problems, meta-heuristic algorithm, swarm intelligence

## Abstract

The Crayfish Optimization Algorithm (COA) has limitations that affect its optimization performance seriously. The competition stage of the COA uses a simplified mathematical model that concentrates on relations of distance between crayfish only. It is deprived of a stochastic variable and is not able to generate an applicable balance between exploration and exploitation. Such a case causes the COA to have early convergence, to perform poorly in high-dimensional problems, and to be trapped by local minima. Moreover, the low activation probability of the summer resort stage decreases the exploration ability more and slows down the speed of convergence. In order to compensate these shortcomings, this study proposes an Improved Crayfish Optimization Algorithm (ICOA) that designs the competition stage with three modifications: (1) adaptive step length mechanism inversely proportional to the number of iterations, which enables exploration in early iterations and exploitation in later stages, (2) vector mapping that increases stochastic behavior and improves efficiency in high-dimensional spaces, (3) removing the X_shade_ parameter in order to abstain from early convergence. The proposed ICOA is compared to 12 recent meta-heuristic algorithms by using the CEC-2014 benchmark set (30 functions, 10 and 30 dimensions), five engineering design problems, and a real-world ROAS optimization case. Wilcoxon Signed-Rank Test, *t*-test, and Friedman rank indicate the high performance of the ICOA as it solves 24 of the 30 benchmark functions successfully. In engineering applications, the ICOA achieved an optimal weight (1.339965 kg) in cantilever beam design, a maximum load capacity (85,547.81 N) in rolling element bearing design, and the highest performance (144.601) in ROAS optimization. The superior performance of the ICOA compared to the COA is proven by the following quantitative data: 0.0007% weight reduction in cantilevers design (from 1.339974 kg to 1.339965 kg), 0.09% load capacity increase in bearing design (COA: 84,196.96 N, ICOA: 85,498.38 N average), 0.27% performance improvement in ROAS problem (COA: 144.072, ICOA: 144.601), and most importantly, there seems to be an overall performance improvement as the COA has a 4.13 average rank while the ICOA has 1.70 on CEC-2014 benchmark tests. Results indicate that the improved COA enhances exploration and successfully solves challenging problems, demonstrating its effectiveness in various optimization scenarios.

## 1. Introduction

Meta-heuristic algorithms (MHAs) and mathematical approaches are frequently used for solutions to optimization problems. The success of mathematical approaches depends directly on the initial fitness value and the slope of the gradient descent [1]. As the complications of global optimization problems contribute to these disadvantages, there seems to be a growing interest in algorithms developed through the inspiration of natural phenomena [2]. MHAs are defined as techniques developed in order to enable acceptable solutions for optimization problems whose solutions are difficult under the conditions of inadequate knowledge and limited calculation time. MHAs are not solution methods that are intrinsic to a problem. However, they could produce satisfactory solutions for optimization problems. MHAs could produce such solutions without scanning the whole solution space. Such ability of MHAs depends on a high level of procedures like exploration and exploitation. MHAs also provide us with the following advantages [3,4].

1. Global search: they could search the whole solution space effectively and discover the best fit solution for the problem.
2. Scalability: they could be applied to high-dimensional, non-linear, and continuous or discrete problems.
3. Diversity: since the whole solution space is scanned in global search, population diversity is generated.
4. Adaptability: MHAs could be applied to various problems in finance, economy, engineering, medicine, etc.
5. Computational efficiency: MHAs generate acceptable results in a reasonable time. This property of MHAs presents them as advantageous compared to exact methods for the solution of high-scaled optimization problems.
6. Gradient descent: MHAs do not need gradient descent information. Moreover, the mathematical models of MHAs are simple.

In general, MHAs could be classified into five categories, which are (i) population-based algorithms [5], (ii) evolution-based algorithms [6], (iii) physics/chemistry-based algorithms [7], (iv) human-based algorithms [8], and (v) mathematics-based algorithms [9]. Moreover, it should be noted that there also are algorithms which are developed through the inspiration of music and are not classified above [10]. These classifications and the algorithms within these classifications are studied almost in every article concerned with MHAs. As these articles are studied, it could be observed that the categorization is frequently revisited. The MHAs given as the examples of classification are almost the same as each other, whereas many new and successful algorithms are constantly offered. This study considers that these kinds of subjects are the subject matter of typology and review articles. Therefore, at first, this study explains why MHAs are still proposed even though there are many. Then, it discusses about what to focus on while picking or improving an existing MHA.

Many meta-heuristic algorithms are proposed by researchers in the literature [11]. Some of them are more productive compared to their competitors. The improved versions of the most of them are presented as well. Nonetheless, new MHAs are still developed [12]. As a result of the rapid advancement in technology, the difficulty level of optimization problems to be solved in various industries has been increasing dramatically [13,14]. Such a case makes the development of MHAs with innovative strategies necessary [15]. Moreover, even though existing MHAs are successful in solving some kinds of optimization problems, some of them may not be successful enough to solve some other kinds [16]. This could be explained by the early convergence or slowness of the speed of convergence. In addition to these, as stated in the No Free Lunch Theorem, an MHA could not solve all optimization problems successfully [17]. Once MHAs are applied to all optimization problems, they seem to have similar performances. Thus, it could be concluded that MHAs could compete with each other in terms of a problem or set of problems.

An MHA is studied in four ways in the literature. These are (1) the original article of an MHA, (2) the application of an original MHA to an optimization problem, (3) the application of an MHA by hybridizing it with another MHA, and (4) improving the MHA by changing its mathematical model and applying it to an optimization problem.

As stated earlier, all MHAs have similar performances for all optimization problems. Therefore, while picking an MHA, in addition their results in the original article, a researcher should consider their other properties as well. Picking an MHA specific for a certain problem could increase the chance of success.

MHAs are initiated with a randomly generated population [18]. Then, the fitness value of the population is calculated. According to the strategies of MHAs, the best, top three best, or all fitness values are saved. In many population-based algorithms, there exist exploration and exploitation phases. In the exploration phase, possible solution areas of the search space are determined [19]. In the exploitation phase, the areas determined in the exploration phase are studied thoroughly [20]. In MHAs, the transition between these two phases is made through a probability key. The value of this key is determined generally in two ways; the first one is random, and the second one depends on the function. While determining the probability key randomly, a number between 0 and 1 is produced randomly [21,22]. If this number is greater than the threshold value, which is predetermined and usually 0.5, exploration is performed. However, if it is smaller than that, we follow exploitation (or vice versa). In this method, at low iteration numbers, both exploration and exploitation could be performed. The same goes for high iteration numbers as well. The disadvantage of the method is the slowness of the convergence rate. If the probability key is produced depending on the function, one parameter of the function is generally the available iteration number [4,23,24]. As the iteration number increases, the value of the probability key goes down. Here, the goal is mostly to perform exploration at low iteration numbers and to perform exploitation at high iteration numbers. The disadvantage of the method is being caught in the local minimum trap. Among these methods, it could be stated that the function-based one is, respectively, more successful. However, it should be borne in mind that we should make a choice specific for the problem. Moreover, MHAs could be applied to the relevant problem by changing the way of determining the probability key of an MHA. Lu et al. developed an innovative approach that dynamically controls the balance between exploration and exploitation and integrated it into GWO [25]. In this way, the transition between exploration and exploitation can be managed dynamically, enhancing the algorithm’s ability to adapt to changing optimization conditions.

The presence of initial parameters is an important criterion in the selection of an MHA. In recently developed MHAs, researchers try to avoid or reduce the number of these parameters as much as possible [4,26,27]. Without a doubt, this makes MHAs easier to use. It should be more plausible for inexperienced users to choose MHAs with few or without initial parameters. On the other hand, the presence of initial parameters could be an advantage to turn MHAs problem-specific. Such a case goes for the experienced users. It should be kept in mind that in such a case there needs to be an extra analysis for the adjustment of initial parameters. Developing MHAs with adjustable parameters could be focused on. Adaptive parameter tuning is critical for enhancing the effectiveness of MHAs. Recent studies have demonstrated that meta-learning methods, which become adaptive through approaches based on learning alternative minimization steps, have outstanding performance, especially in non-convex problems [28]. The effectiveness of adaptive parameters has been demonstrated not only through simulations but also experimental works that were conducted on biological search agents [29]. To overcome the challenge of parameter tuning in optimization algorithms, Wang et al. [30] developed an adaptive mechanism using reinforcement learning that automatically adjusts the distribution ratios between high-performing and low-performing individuals in the population based on accumulated rewards, eliminating the need for predefined parameters.

Recent studies have demonstrated that meta-learning methods, which become adaptive through approaches based on learning alternative minimization steps, have outstanding performance, especially in non-convex problems.

In the exploration and exploitation phases of some MHAs, there could be more than one procedure [31]. Which procedure to run is determined by a probability key. Most of the times, this probability key is determined randomly. However, this method, as stated earlier, could be changed. By changing the threshold value according to the problem, further selection of a procedure may be preferable. Because of the scholastic structure of MHAs, there exist random numbers in their mathematical models [32,33]. These random numbers are generally between 0 and 1. By changing this range or by increasing or decreasing the number of random numbers, the performance of the MHA could be analyzed. Analysis could be performed by changing the methods of determining random numbers as well. In the literature, there are many studies where random numbers are determined through chaotic maps [34,35].

In general, for MHAs, being like the best one is a significant matter for search agents. The candidate solution within the available iteration aims to be like the best individual. This method is certainly reasonable. However, it could cause problems like early convergence and being trapped by local minima. Instead of that, being like the mean of the top three, which are called alpha, beta, and gamma and are inspired by the Grey wolf optimizer [36], could avoid such problems. Other than that, simulation to the mean of all solution offers, as in Harris hawk optimization [23,37], could be a smarter way for some problems.

With the quick improvements of meta-heuristic algorithms, the performance limitations of algorithms in hand have been more obvious. In complicated engineering problems, especially, algorithms’ balance of exploration–exploitation, convergence speed, and the ability to escape from local minimum traps have been critical factors of success.

Even though the Crayfish Optimization Algorithm (COA) presents promising results in nature-inspired MHAs, it has three critical limitations in its algorithmic structure. First, the COA uses a simplified mathematical model in the competition stage. In this stage, only the Euclidian distance is paid attention to while elements necessary for effective exploration, such as adaptive step lengths, directional information, and stochastic components, are totally disregarded. Second, the heat-based transition mechanism of the algorithm generates a poor exploration–exploitation balance. Inadequate exploration capacity in early iterations and inadequate exploitation improvements in later stages cause suboptimal convergence characteristics and early convergence. Third, the algorithm’s lack of applicable mechanisms in high-dimensional problems causes it to have low performance as the problem becomes more complicated and limits its applicability to real-world engineering optimization scenarios.

These limitations, especially in high-dimensional and multi-modal optimization problems, decrease the effectiveness of the COA significantly, increase the risk of the algorithm being trapped by local minima, and slow down the convergence rate. Such a case indicates the necessity to redesign and improve the basic algorithmic components of the COA.

The subject of this work is the COA, which is a population-based MHA [38]. It is inspired by the way Crayfish search for food, avoid heat, and compete each other. The COA has a two-level search strategy made of exploration and exploitation. Even though it is a successful MHA, it faces the possibility of being trapped by local minimum traps. The literature indicates that researchers mainly concentrate on the temperature parameter, the balance between exploration and exploitation, and exploration. In a study that aims to overcome such problems, researchers determine four strategies to improve the COA [39]. These strategies are the halton sequence, opposition-based learning, elite steering factor, and fish aggregation device effect. They inform us that these strategies increase the diversity of the population and the convergence rate as well as accelerate the convergence. Jia et al. propose two strategies in order to accelerate the convergence of the COA and prevent it from being trapped by local minima [40]. These strategies are the environmental renewal mechanism and ghost opposition-based learning. It is noted that the improvements increase the global optimization performance of the algorithm. The ECOA, improved by Yuan et al., has increased the performance of the COA through three basic improvements [41]. These improvements are the dynamic lens-imaging learning strategy that enriches population diversity, the dynamic decline curve that expands the search space and increases the convergence speed, and the novel feeding strategy that strengthens the local optimization capability of the algorithm. Zhang and Diao [42] proposed the Hierarchical Learning-enhanced Chaotic Crayfish Optimization Algorithm (HLCCOA) by integrating chaotic mapping and hierarchical learning mechanisms, solving the problem of avoiding local optima by increasing population diversity. In this study, a population initialization strategy was developed using Tent and Chebyshev chaotic maps, and individuals in the population were divided into hierarchical layers according to their fitness values, applying different learning mechanisms.

The aim of this study is to present an improved version of the COA [38], which is a population-based MHA. It offers an improved version of the COA by changing its mathematical model. This new version is called the Improved COA (ICOA). In the COA, the transition between exploration and exploitation is performed through heat. The temperature parameter of the COA varies between 15 and 35 °C. The temperature is determined depending on a random number. This means that the COA could perform exploration and exploitation at low or high iteration numbers. The COA performs exploitation between 15 and 30 °C (foraging stage). When the temperature is above 30 °C, there comes the summer resort stage (exploration) or competition stage (exploitation). Which phase to perform is determined by a random parameter. As it could be understood through the information given earlier, the probability of the COA performing exploration is relatively low. This causes the COA to be trapped by local minima and slow convergence speed. The competition stage of the COA is designed for local search. However, the foraging stage already does that. Thus, the competition stage of the COA is updated considering the exploration properties of exploration.

The main contributions of this study are given as follows.

- At the competition stage of the COA, crayfish have to compete with the other crayfish in order to go in the cave. In the original COA, this competition is modeled only by distance. In the ICOA, besides the distance, the step length and locations of crayfish are added to the model.
- The step length of crayfish differs depending on the iteration. Big steps are produced at low iteration numbers while at high iteration numbers, there occur small steps. Such a case allows the competition stage to help exploration at low iteration numbers and to help exploitation at high iteration numbers.
- In addition, at the competition stage of the ICOA, the cave location, which represents the best location, is removed from the mathematical model. The reason for that is to increase the exploration ability of the ICAO. Cave locations are also available in other stages.
- To verify the validity of the ICOA, it was compared to nine MHAs. For comparison, the CEC-2014 dataset with 30 test functions was used. Five engineering design problems were used for comparison as well.
- The results are interpreted by the Wilcoxon Signed-Rank Test and Friedman test.

The rest of this study is organized as follows. In the second section, information about the COA and its mathematical model is given. In the third section, the ICOA is identified. In the fourth one, the results of the ICOA and 12 competitors are compared. For comparison, the CEC-2014 dataset and five engineering design problems are used. CEC-2014 tests are performed in two different dimensions D=10,D=30. In the last one, the results are discussed for future studies.

**Figure 1:**
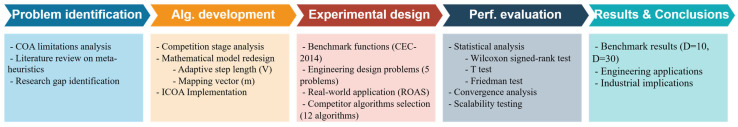
Research flow diagram.

The research methodology of this study has six main stages, as indicated in Figure 1. In the first stage, the limitations of the COA algorithm were analyzed and the research gap was introduced through the literature review. In the second stage, the ICOA was improved by redesigning the mathematical model of the competition stage. In the third stage, a comprehensive experimental design, including CEC-2014 benchmark functions, engineering design problems, and real-world application, was created. In the fourth stage, through statistical tests and convergence analysis, the performance of the ICOA was evaluated. In the fifth stage, the results were verified and industrial implications were analyzed. The last stage presented the conclusion and proposed suggestions for future studies.

## 2. Crayfish Optimization Algorithm (COA)

The COA is an MHA developed through the inspiration of the foraging, summer vacation, and competitive behavior of crayfish. The foraging stage and competition stage make the exploitation stage of the COA while the summer vacation stage makes its exploration stage. The algorithm is initiated, generating the population randomly. In the COA, the locations of crayfish are denoted by ‘$X_{i,j}$’, where X is the location vector of the crayfish, and i and j are, respectively, the dimension of the population and the problem.

### 2.1. Define Temperature and Crayfish Intake

The change in the ambient temperature is effective upon the feeding behavior of crayfish. The best feeding temperature for crayfish is 25 °C. These creatures could feed between 15 °C and 30 °C. The temperature parameter enables transition between exploration and exploitation. Exploration is performed when the temperature is above 30 °C while exploitation occurs when it is below 30 °C. The temperature is calculated by Equation (1). The mathematical model of crayfish intake is shown in Equation (2).

(1) temp=rand×15+20

(2) $$p=C_{1}\times \left(\frac{1}{\sqrt{2\times \pi }\times \sigma )}\times exp\left(-\frac{(temp-\mu {)}^{2}}{2\sigma^{2}}\right)\right)$$

In the equations, rand is the randomly produced number between 0 and 1. μ is the ideal temperature for the crayfish. σ and $C_{1}$ is the intake of crayfish control parameter at different temperatures.

### 2.2. Summer Resort Stage (Exploration)

When the temperature is above 30 °C, it is not fit for crayfish to feed. Thus, they prefer to go inside the cave. The cave locations $X_{shade}$ are denoted as below (Equation (3)).

(3) $$X_{shade}=\left(X_{G}+X_{L}\right)/2$$

where $X_{G}$ represents the best location up to the available iteration while $X_{L}$ represents the best location within the available iteration.

There are circumstances where crayfish either do or do not compete with each other in order to go in the cave. It is assumed that there is no competition among crayfish in the summer resort stage. The stage at which they compete each other for the cave is called the competition stage, which is explained in the following subsection. The transition between these two stages is through a random number. If Rand<0.5, crayfish go directly into the cave (Equation (4)), otherwise they will have to compete with the other ones (competition stage).

(4) $$X_{i,j}^{t+1}=X_{i,j}^{t}+C_{2}\times rand\times \left(X_{shade}-X_{i,j}^{t}\right)$$

where t represents the current iteration while t+1 represents the next iteration number. $C_{2}$ is calculated as follows (Equation (5)).

(5) C2=2−tT

where T represents the maximum iteration number. This stage is designed for the purpose of increasing the convergence speed of the COA.

### 2.3. Competition Stage (Exploitation)

If the temperature is above 30 °C and rand≥0.5, the ambient temperature is not fit for the crayfish to feed. Therefore, crayfish go to the cave once again. However, this time, they have to compete with the other crayfish for the cave. The mathematical model of this is given in Equation (6).

(6) $$X_{i,j}^{t+1}=X_{i,j}^{t}-X_{z,j}^{t}+X_{shade}$$

where i represents the available individual, while z represents the randomly picked individual and is calculated through Equation (7).

(7) $$z=round\left(rand\times \left(N-1\right)\right)+1$$

where N is the population. This step is designed to improve the exploration of the COA.

### 2.4. Foraging Stage (Exploitation)

If the ambient temperature is below 30 °C, it is fit for the crayfish to feed. Now, the crayfish move towards the food. The size of the food is a criterion for feeding. If the food is big, the crayfish crumble the food. The location of the food is defined through Equation (8) and the size of it is defined through Equation (9).

(8) $$X_{food}=X_{G}$$

(9) Q=C3×rand×fitnessifitnessfood

where $C_{3}$ is the food factor of the biggest food and its value is 3. ${fitness}_{i}$, i. represents the fitness value of the crayfish while ${fitness}_{food}$ represents the fitness value of the location of the food. $X_{food}$ represents the best solution. Crayfish decide whether food is large or not based on the largest piece of the food. If $Q>\left(C_{3}+1\right)/2$, it means the food is big. Then, they crumble the food. The mathematical model of this is given in Equation (10).

(10) $$X_{food}=exp\left(-\frac{1}{Q}\right)\times X_{food}$$

Crayfish eat the food after they make it smaller. This is modeled in Equation (11).

(11) $$X_{i,j}^{t+1}=X_{i,j}^{t}+X_{food}\times p\times \left(\cos\left(2\times \pi \times rand\right)-\sin\left(2\times \pi \times rand\right)\right)$$

If $Q\leq \left(C_{3}+1\right)/2$, there is no need to crumble the food. Crayfish could eat the food as is (Equation (12)).

(12) $$X_{i,j}^{t+1}=\left(X_{i,j}^{t}-X_{food}\right)\times p+p\times rand\times X_{i,j}^{t}$$

At the food stage, crayfish have two kinds of eating behavior depending on the size of the food. This stage is designed to improve the convergence ability of the COA.

## 3. Improved Crayfish Optimization Algorithm

Just like many swarm-based optimization algorithms, the COA consists of exploration and exploitation stages. The transition between exploration and exploitation is performed through temperature. This means that in the COA, exploration and exploitation could be performed regardless of iteration. The exploitation stage of the COA (foraging stage) is two-phased depending on the size of the food. However, the exploration stage of the COA is two-phased depending on the randomly determined parameter. These stages are the summer resort stage and competition stage.

In the preliminary investigations aimed at improving the performance of the COA, it is determined that the competition stage is modeled too simplistically. At this stage, crayfish have to compete with other crayfish to reach the cave, i.e., the best solution. This competition is modeled only through the distance between the crayfish. Moreover, because of the stochastic structure of MHAs, the components based on stochastic parameters are not present in this model. In addition to these, $X_{shade}$ is used at the summer resort stage. Using it at the competition stage causes the exploration ability of the COA to decrease and to be caught by the local minimum trap. Updating the process of being like the best one with an alternative model that scans the search space more efficiently could improve the efficiency of the COA. The remodeling of the competition stage is inspired by the exploration stage of Artificial Rabbits Optimization [4]. When crayfish compete for the cave, what is important is not only their distance between each other but also their speed of movement. Thus, the V parameter, which represents their speed of movement and step length, is added to the model. Since each crayfish represents a location, a mapping vector m is added to the model as well. In addition, $X_{shade}$ is removed from the equation in order to prevent the early convergence. No other modifications are made at the other stages of the COA. The mathematical model of the Improved Competition stage is given in Equation (13).

(13) $$X_{i,j}^{t+1}=X_{i,j}^{t}+V\cdot m\cdot \left(X_{i,j}^{t}-X_{z,j}^{t}\right),\;i,z=1,\ldots ,n\;and\;i\neq z$$

(14) $$V=\left(e^{\left(1-{\left(\frac{t-1}{T}\right)}^{4}\right)}-1\right)\cdot \cos\left(2\pi r_{1}\right)$$

(15) $$m\left(k\right)=\left\{\begin{array}{c} 1,\;\;if\;k==g\left(l\right)\\ 0,\;\;else \end{array}\;k=1,\;\ldots ,\;d\;and\;l=1,\;\ldots ,\;\left\lceil r_{2}\cdot d\right\rceil \right.$$

**Figure 2:**
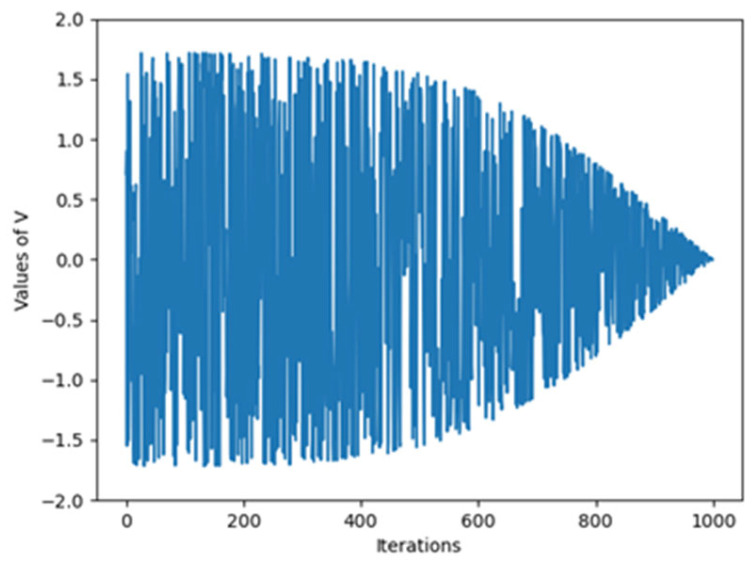
Dynamic behavior of V.

(16) g=randperm(d)

(17) $$z=round\left(rand\times \left(N-1\right)\right)+1$$

where V denotes the step length of the crayfish and is calculated through Equation (14). m represents the mapping vector and it is calculated through Equation (15). $r_{1}$ and $r_{2}$ are randomly determined numbers between 0 and 1. d is the dimension of the problem. There is an inverse proportion between step length (V) and iteration number (t). However, this inverse proportion is not linear. In Figure 2, the dynamic behavior of V is given. The long steps at low iteration numbers enable more efficient exploration while short steps at high iteration numbers enable more efficient exploitation. randperm returns the random permutation of the integers from 1 to d (Equation (16)). z represents the randomly picked individual and is calculated through Equation (17).

Equation (13) makes up the basic updating mechanism of the ICOA. Depending on another randomly picked agent, the positions of search agents are updated through adaptively determined step length (V) and map vector (m). This approach not only enables sufficient diversity within the solution space but also allows the solution to concentrate on the best regions for the subsequent iterations. $\left(V\right)$ has a sharp and fast decreasing structure. This aspect of (V) allows the ICOA to perform broad-scale searches at low iterations and narrow-scale searches at high iterations. (m) is used to increase efficiency in high-dimensional problems. In the literature, similar models of this mechanism have been successfully implemented in differential evolution and other MHAs. The theoretical validity of the formulation is based on the combination of adaptive parameter control and randomness to avoid local optimums and to enable fast convergence.

The pseudo-codes of the COA and ICOA are given in Algorithm 1.
**Algorithm 1.** Pseudo-codes of algorithms.**COA pseudo-code****ICOA pseudo-code****Input: *T:*** maximum iteration, ***N:*** population size, ***D:*** variable dimension**Input: *T:*** maximum iteration, ***N:*** population size, ***D:*** variable dimension**Output:** The optimal search agent $X_{best}$, and its fitness value $f_{best}$
**Output:** The optimal search agent $X_{best}$, and its fitness value $f_{best}$
Generate initial population Calculate the fitness value of the population to get $X_{G}$, $X_{L}$ While t<T          Defining temperature temp by ***Equation (1)***             If temp>30                    Define cave $X_{shade}$ according to ***Equation (3)***                        If rand<0.5                          Crayfish conducts the summer resort stage according to ***Equation (4)***                        Else                          Crayfish compete for caves through ***Equation (6)***                        End          Else                    The food intake *p* and food size Q are obtained by ***Equation (2)*** and ***Equation (9)***                        If Q>2                          Crayfish shreds food by ***Equation (10)***                          Crayfish foraging according to ***Equation (11)***                        Else                          Crayfish foraging according to ***Equation (12)***                        End          End          Update fitness values, $X_{G}$, $X_{L}$          t=t+1 End
Generate initial population Calculate the fitness value of the population to get $X_{G}$, $X_{L}$ While t<T          Defining temperature temp by ***Equation (1)***          If temp>30                    Define cave $X_{shade}$ according to ***Equation (3)***                    If rand<0.5                              Crayfish conducts the summer resort stage according to ***Equation (4)***                    Else                              Updated competition stage ***Equation (13)***                    End          Else                    The food intake *p* and food size Q are obtained by ***Equation (2)*** and ***Equation (9)***                    If Q>2                              Crayfish shreds food by ***Equation (10)***                              Crayfish foraging according to ***Equation (11)***                    Else                              Crayfish foraging according to ***Equation (12)***                    End          End          Update fitness values, $X_{G}$, $X_{L}$          t=t+1 End


## 4. Computational Results and Discussions

### 4.1. Experimental Settings and Compared Algorithms

The proposed algorithm is compared to Particle Swarm Optimization (PSO) [43], Differential Evolution (DE) [44], the Whale Optimization Algorithm (WOA) [45], Arithmetic Optimization Algorithm (AOA) [46], African Vultures Optimization Algorithm (AVOA) [47], Golden Jackal Optimization (GJO) [48], Sea-Horse Optimizer (SHO) [49], Energy Valley Optimizer (EVO) [26], Human Conception Optimizer (HCO) [50], RUNge Kutta optimizer (RUN) [51], and the Brown-Bear Optimization Algorithm (BBOA) [27], which are COA algorithms that verified their up-to-datedness and validity. While choosing these algorithms, some criteria are taken into consideration. PSO, DE, WOA, EVO, and BBOA are chosen because the first three are mainstream algorithms while the last two do not have initial parameters. Other algorithms are chosen depending on the few or many initial parameters they have. Being up to date and having verified validity are other significant selection criteria.

Algorithms are coded in the Python language. Tests are run in a computer with Windows 10 64 bit Professional and 64 GB of RAM. Results of 30 independent runs of all algorithms are saved. The population size and maximum function evaluations (FEs) of each MHA are, respectively, set to 50 and 10,000. The comparison of the algorithms depends on the best (Min), average (Ave), and standard deviation (Std) parameters. The performance of the algorithms is evaluated by Wilcoxon Signed-Rank Test (WSRT) with 5% significance level. R+ ve R− are calculated using WSRT. R+ means that the proposed algorithm has a better performance than the competitor algorithm. On the other hand, R− means that the proposed algorithm has a weaker performance compared to its competitor. Total R+ and total R− values are given as T+ and T− in the tables [3,52]. In the tables where WSRT statistics are given, there is also a column with a ‘W’ symbol. This column denotes the winner algorithm. If there is a ‘+’ symbol in this column, the winner is the ICOA. However, if the symbol is ‘−’, the winner is the competitor algorithm. Other than these, the radar graphics and the convergence curves of the algorithms are given as well.

In order to compare the proposed algorithm to the competitor one, we used the CEC-2014 dataset with 30 hard functions [53]. CEC-2014 consists of three unimodal functions f1−f3, 13 simple multimodal functions f4−f16, six hybrid functions (f17−f22), and eight composition functions (f23−f30). Unimodal functions test the exploitation ability of MHAs. Multimodal functions test the exploration power of the optimizers and the ability to avoid local minimums. Hybrid functions possess many local minimums and a good optimization algorithm should avoid these local minimums. Composite functions test the balancing ability of MHAs between exploration and exploitation. The search ranges for all the problems are $\left[-100,\;100\right]$. In this study, the dimensions of the problems (D) are taken as 10 and 30. The initial parameters of the ICOA and competitor algorithms are given in Table 1. In this study, the initial parameters are taken from the original articles of the algorithms. MHAs are highly sensitive to initial parameters and they affect their performance directly. The original articles of MHAs work on fine tuning the parameters and determine the best values. Thus, for a just comparison, the initial parameters of MHAs should be taken from the original articles. Accordingly, in this study, the initial parameters are taken from the original articles of the algorithms.

### 4.2. CEC-2014 Benchmark Function Results

For D=10, minimum, average, and standard deviation values of all the algorithms by the CEC-2014 test functions are given in Table 2, Table 3, Table 4 and Table 5. In the minimum value metric, the ICOA solves 23 of the 30 test functions successfully. The AVOA, COA, DE, WOA, SHO, GJO, and RUN, respectively, solve six, five, four, two, two, two, and two test functions successfully. In the average value metric, the ICOA has successful results in 22 test functions. The AVOA, COA, DE, and RUN, respectively, solve five, four, three, and two test functions successfully. For D=30, minimum, average, and standard deviation values of all the algorithms by the CEC-2014 test functions are given in Table 6, Table 7, Table 8 and Table 9. In the minimum value metric, the ICOA has successful results in 24 of 30 test functions. The AVOA, COA, GJO, SHO, and BBOA are competitive, respectively, in 13, seven, five, five, and four test functions. In the average value metric, the ICOA has competitive results in 23 test functions. The AVOA, COA, DE, BBOA, SHO, and GJO are successful, respectively, in nine, seven, two, two, two, and two test functions. Once the results are interpreted, the ICOA seems to have competitive results. The AVOA and COA are two other successful algorithms.

For D=10 in Table 2, and for D=30 in Table 6, the results of unimodal functions are given. For D=10, the ICOA is superior to its competitors in terms of all valuation criteria. For D=30, the AVOA is better than the ICOA at the minimum value of f1 functions. However, at the average value, the ICOA outperforms all other competitors. The ICOA’s superiority in mean value indicates that its ability to give better results constantly is better than the AVOA. The ICOA also outperforms its competitors in f2 and f3 functions.

For D=10 in Table 3, and for D=30 in Table 7, the results of multimodal functions are given. For D=10, the ICOA has better results than its competitors in eight functions (f4,f6,f7,f8,f9,f11,f13,f15). At the minimum value of the f8 function, there is equality between the ICOA, AVOA, and DE. The AVOA is successful in the functions of f12 and f14, the COA, f10 and f16, RUN, f5. DE is successful in the functions of f14 and f16, the COA, f10, the AVOA, f12 RUN, f5. For D=30, the ICOA solves 10 functions successfully (f4,f5,f6,f7,f8,f9,f10,f11,f14,f16). At the minimum value of the f5 function, the AVOA is more successful. The AVOA is successful in f12, f13, and f15 functions. It is a significant result that once the dimension increases, there is not a function in which the COA is successful; and there are a number of functions in which the ICOA is successful.

For D=10 in Table 4, and for D=30 in Table 8, the results of hybrid functions are given. For D=10, the ICOA outperforms its competitors in five functions (f17,f18,f19,f20,f21). At the minimum value of the f22 function, while the ICOA is more successful, at the mean value, RUN is more successful. For D=30, the ICOA is the best optimizer in five functions (f17,f18,f20,f21,f22).

For D=10 in Table 5 and for D=30 in Table 9, the results of composition functions are given. For D=10, the ICOA has competitive results in six functions (f23,f24,f27,f28,f29,f30). For f23, f28, f29, and f30, more than one optimizer has similar results. For f24 and f27, the ICOA is the most successful algorithm, for f25, PSO, and for f26, the AVOA. For D=30, the ICOA has successful results in six functions (f23,f24,f25,f27,f28,f29). Moreover, the ICOA has a successful result at the minimum value of the f30 function. More than one optimizer has successful results in other functions except for the f26 function. For f26, the AVOA is the most successful optimizer.

On the last lines of Table 5 and Table 9, the average rank values of all optimizers by the Friedman test are given.

**Figure 3:**
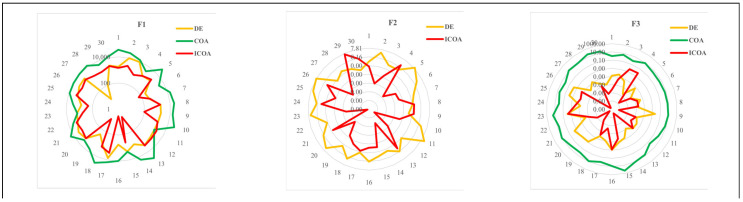

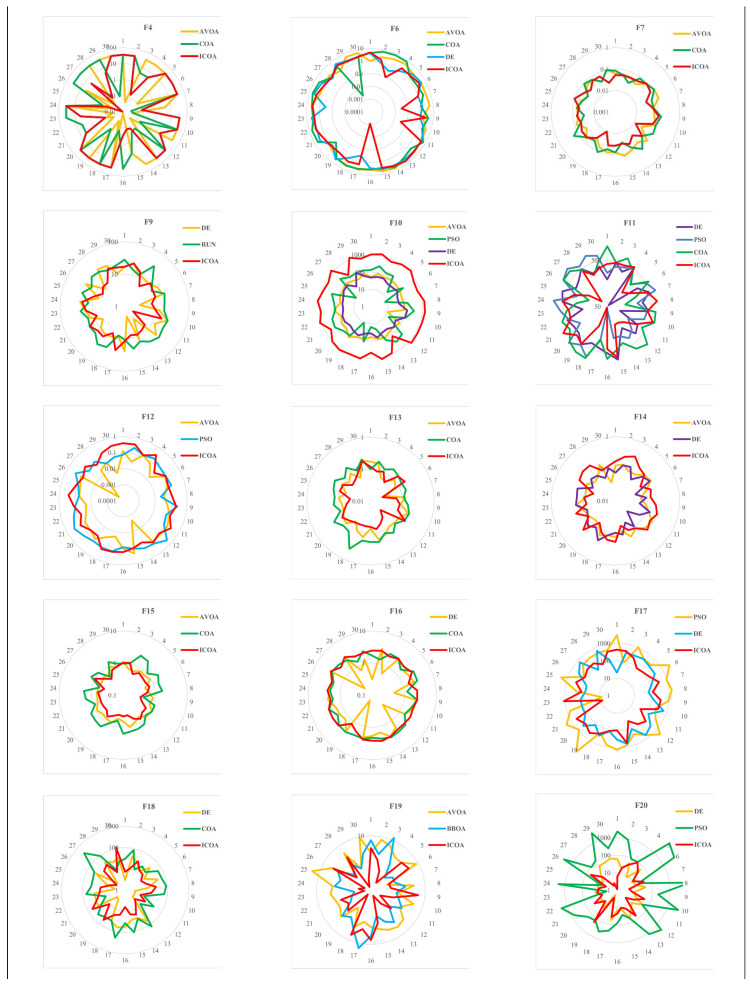

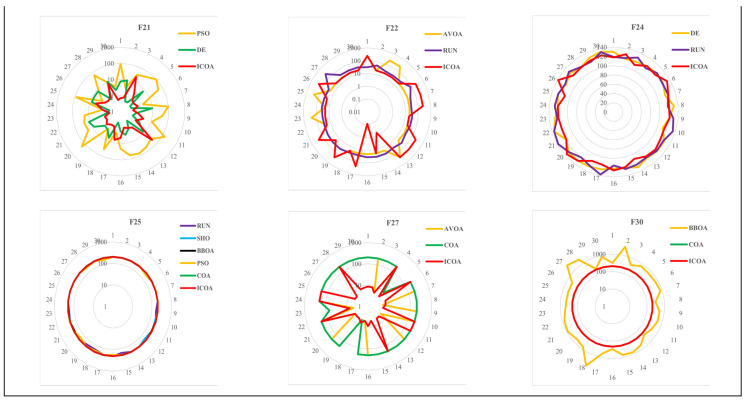
Results of some algorithms for CEC-2014 (D = 10).

**Figure 4:**
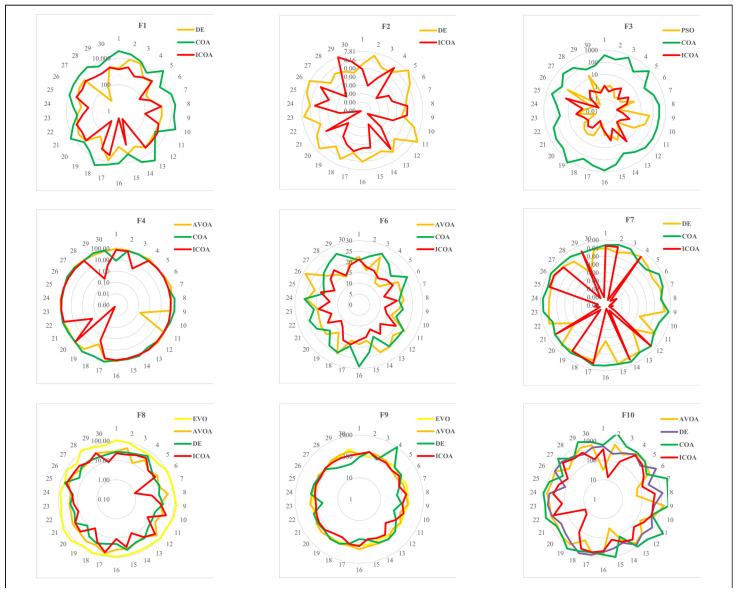

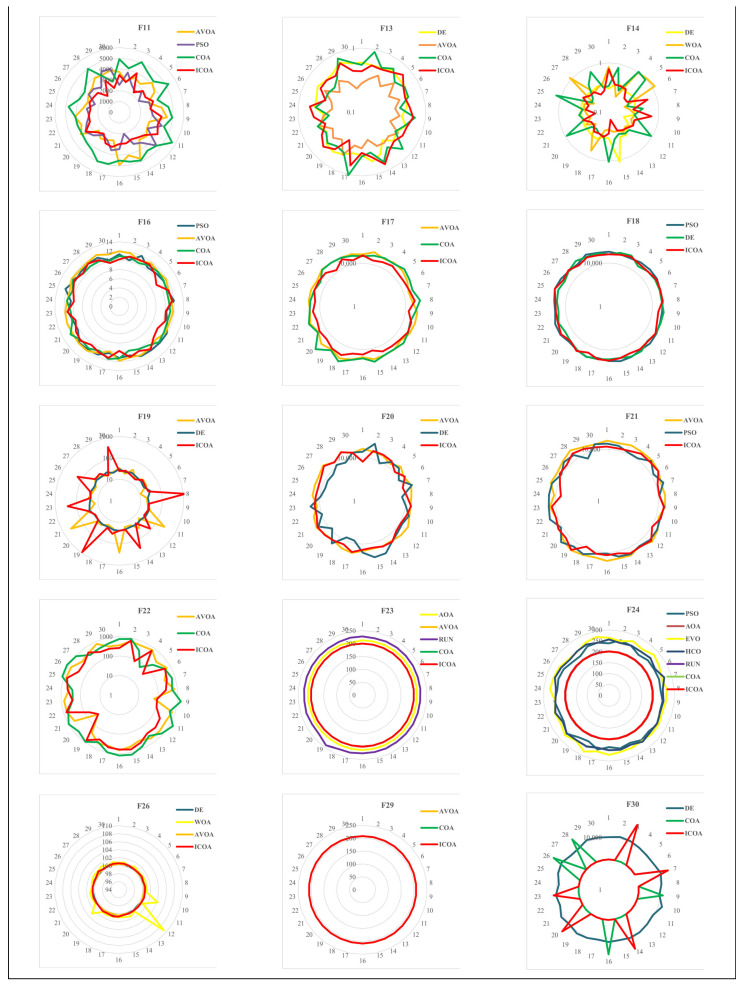
Results of some algorithms for CEC-2014 (D = 30).

For D=10, the ICOA seems to be the best optimizer with 2.12. The AVOA is the second with 3.50, and DE is the third best optimizer with 3.93. For D=30, the ICOA is the most successful algorithm with 1.70. The AVOA and DE are the second and the third most successful algorithms, respectively, with 2.77 and 3.95. For D=10 in Figure 3, and for D=30 in Figure 4, the results of some successful algorithms in some functions are shown through radar graphic. Once the radar graphics are studied, the ICOA, AVOA, and DE seem to be more successful than other algorithms. The ICOA gives more consistent results than its competitors. Interpreting algorithms by analyzing only numerical results may lead to incorrect or incomplete interpretations. For this reason, we perform statistical analysis of the results. We also evaluate the convergence curves of the algorithms. While doing these analyses, we interpret the algorithms.

For an MHA to be regarded as successful, its results need to be supported statistically. For D=10, the WSRT results of the ICOA and competing algorithms are given in Table 10, Table 11, Table 12 and Table 13; and for D=30, in Tables 15–18. In addition, the WSRT statistics are summarized according to the subclasses of the CEC-2014 dataset in Table 14 for D=10, Table 15, Table 16, Table 17 and Table 18, and in Table 19 for D=30.

Once Table 14 and Table 19 are studied, it is observed that the ICOA in all the unimodal functions (f1−f3) outperforms its competitors significantly. Unimodal functions test the exploitation abilities of MHAs. The most significant result at this point is that the ICOA outperforms the COA. The mathematical model of the competition stage of the original COA is more fit for local search. While improving the ICOA, the competition stage is updated to increase the exploration ability. The step length (V) in the mathematical model of the updated competition stage is inversely proportional to iteration. The competition stage helps local search at high iteration numbers. Therefore, the ICOA is successful in unimodal functions.

Multimodal functions are important criteria that are used for evaluating the capability of optimization algorithms to escape from local optima and to search globally. Studying the results for the D=10 dimension, it is observed that the ICOA is highly superior to all its competitors. It is superior to its significant competitors like PSO (8/4/1), DE (7/3/3), the AVOA (10/0/3), and the COA (10/1/2) but there is not a significant difference for some problems. This means that the ICOA has a very good ability to reach global optimums and escape from local optimums in low-dimensional multimodal problems. Studying the results for D=30 dimensions, the ICOA’s superiority seems to be more obvious. It is observed that it outscores the algorithms of PSO (8/5/0), DE (11/2/0), the AVOA (11/0/2), and the COA (11/2/0). For very few problems, there is not a significant difference among algorithms or the competitor algorithms could not outperform the ICOA. This clearly indicates that the ICOA has a very effective global search capability compared to its competitors in high-dimensional multimodal problems. The reason why the ICOA outperforms its competitors, especially the COA, is its ability of exploration. The original COA has an $X_{shade}$ parameter at the competition stage and also does not have a stochastic component. Therefore, its ability to explore in the search space is limited. At the improved competition stage of the ICOA, there is no orientation to the best one and optimality, and it is based on randomness. The goal is to search the solution space more effectively. This is based on the step length (V) and the mapping factor (m).

Hybrid functions (f17−f22) are kinds of complicated functions that evaluate both the global and the local search capabilities of optimization algorithms. Having successful results out of these functions indicates that algorithms have a balanced capability of exploration and exploitation. Examining the results of hybrid functions with the D=10 dimension, the ICOA seems to outperform all the other algorithms. Only in one function is there not a significant difference between the ICO and PSO, the AVOA, BBOA, and RUN. Moreover, the competitor algorithms are not more successful than the ICOA in a function. This indicates that the ICOA has very balanced and superior performance in low-dimensional hybrid problems. The performance of the ICOA is again effective in D = 30 dimensional functions. Only in two functions does there not seem to be a significant difference between PSO and the ICOA. However, other algorithms have never been more successful than the ICOA. Hybrid functions have much higher local minimums than usual. These functions test MHAs’ power to avoid local minima. The results indicate that the ICOA is able to avoid the local minimum trap. This can be explained by the fact that the competition stage helps local search at high iteration numbers.

Once the data in Table 14 (D=10) and Table 19 (D=30) are studied, it is observed that the ICOA is more competitive than AOA, EVO, HCO, and RUN in eight composite functions (f23−f30). For D=10, the ICOA is more successful than six functions in SHO, BBOA, and GJO. In f23,f25 functions, there is not a significant difference between these algorithms. The ICOA has better results than the AVOA (f24,f27,f30) and COA (f24,f26,f27) while they have similar results in five functions. For D=30, the ICOA outperforms SHO, BBOA, and GJO in five functions but they have similar results in three functions (SHO: f24,f25,f29/BBOA: f24,f25,f29/GJO: f24,f25,f28). The ICOA outperforms the AVOA in one function (f30) but they have similar results in six functions. On the other hand, the AVOA is more successful in one function (f26). While the ICOA outperforms the COA in one function (f26), both of the optimizers have similar results in seven functions. Composite functions test the ability of MHAs to balance the exploration and exploitation phases. In composite functions, the ICOA is clearly superior to its competitors except for the COA and AVOA. There is not a function where it is less successful than the COA. Statistical results indicate that the ICOA performs either better or equal to the COA. For D=10, the ICOA outperforms the AVOA but for D=30, there is not a significant difference between the two algorithms. The difference between the ICOA and COA-AVOA could be explained as follows. To begin with, like the ICOA, the COA and AVOA give the best results in composite functions. Secondly, the ICOA’s transition method between exploration and exploitation is the same as the COA’s. Once this method is made more effective, the ICOA’s performance in composite functions could be enhanced. It should be kept in mind that the subject matter of this study is to generate an MHA with a more effective exploration ability by updating the COA’s competition stage.

In general, according to the data in Table 14 (D=10), the ICOA is the most competitive algorithm, solving 21 out of 30 problems better than the AVOA and 22 out of 30 problems better than the COA. The ICOA outperforms its competitors PSO, DE, WOA, AOA, SHO, BBOA, EVO, GJO, HCO, and RUN in, respectively, 23, 23, 26, 30, 26, 25, 29, 27, 30, and 27 functions. When the data in Table 19 (D=30) is analyzed, the ICOA outperforms the AVOA and COA in 20 out of 30 problems. The ICOA outperforms its competitors PSO, DE, WOA, AOA, SHO, BBOA, EVO, GJO, HCO, and RUN in, respectively, 22, 25, 27, 30, 27, 27, 30, 30, 27, 30, 30, and 30 functions. The statistical results indicate that the method proposed in this study to improve the COA is successful.

In order to evaluate the performance of the ICOA in a more reliable way, a statistical *t* test is performed over the CEC 2014 test functions. The details of the test results are given in Table 20 and Table 21. The summary of test results is given in Table 22. Examining the table, in the case where the dimension (D) is 10, the ICOA is clearly seen to outperform all the algorithms. For example, while it outperforms PSO in 23 functions, it has a lower performance only in two functions; and there is not a significant difference in five functions. Similarly, it outperforms the COA in 21 functions, but is less successful only in three functions; and there is not a significant difference in six functions. Its superiority against HCO, RUN, and AOA in almost all the functions is clearly observed.

In problems with higher dimensions (D=30), the success of the ICOA is more evident. The ICOA shows superior performance in all the functions (30/0/0), especially against RUN, HCO, and AVOA. Compared to other algorithms, it has significantly better results and there is not a significant difference in very few functions. These results clearly point out that the ICOA is more consistent, stable, and has effective performance compared to other algorithms, especially in high-dimensional problems. The reason for the success of the ICOA in high-dimensional problems is that it has the m parameter. The m parameter ensures that only some dimensions are updated in high-dimensional problems. Thus, it both reduces the computational load of the algorithm and controls excessive randomness.

**Figure 5:**
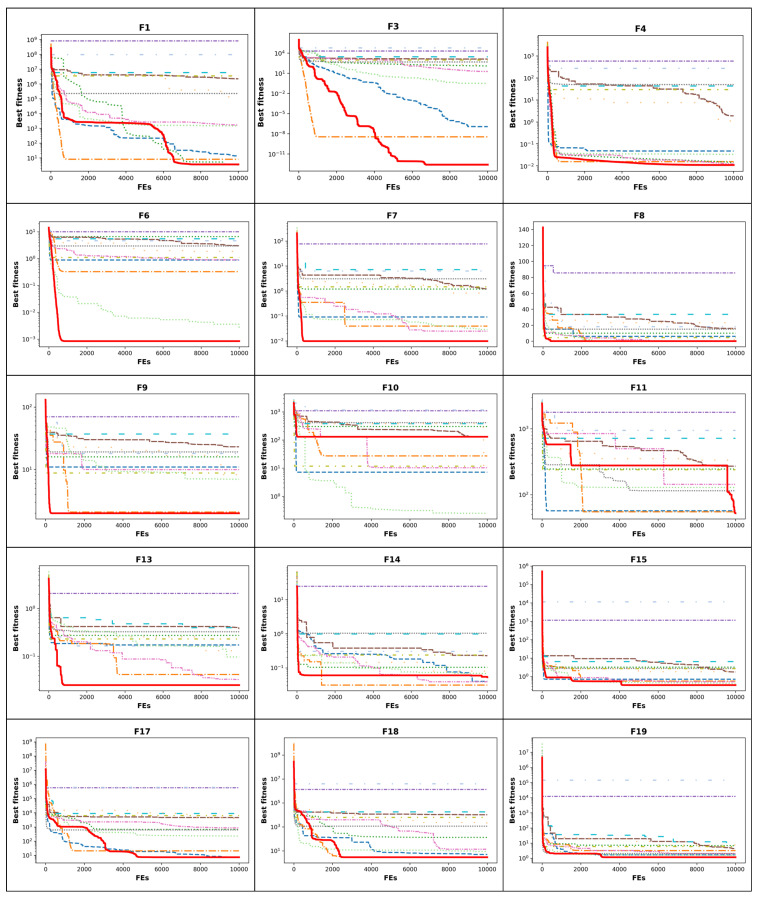

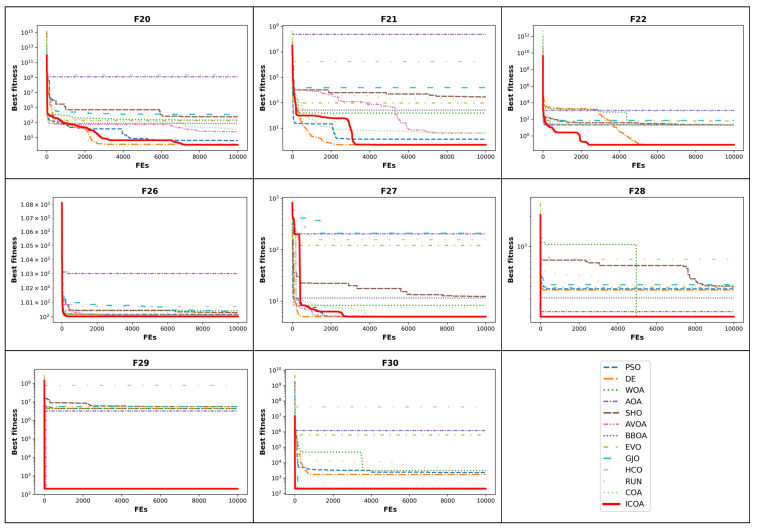
Convergence curves of ICOA and competing algorithms for CEC-2014 (D = 10).

**Figure 6:**
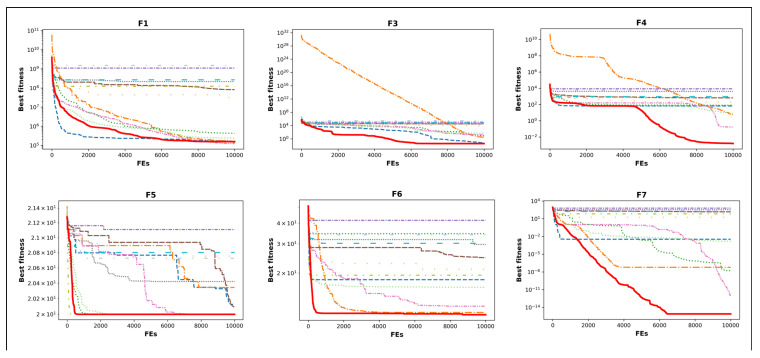

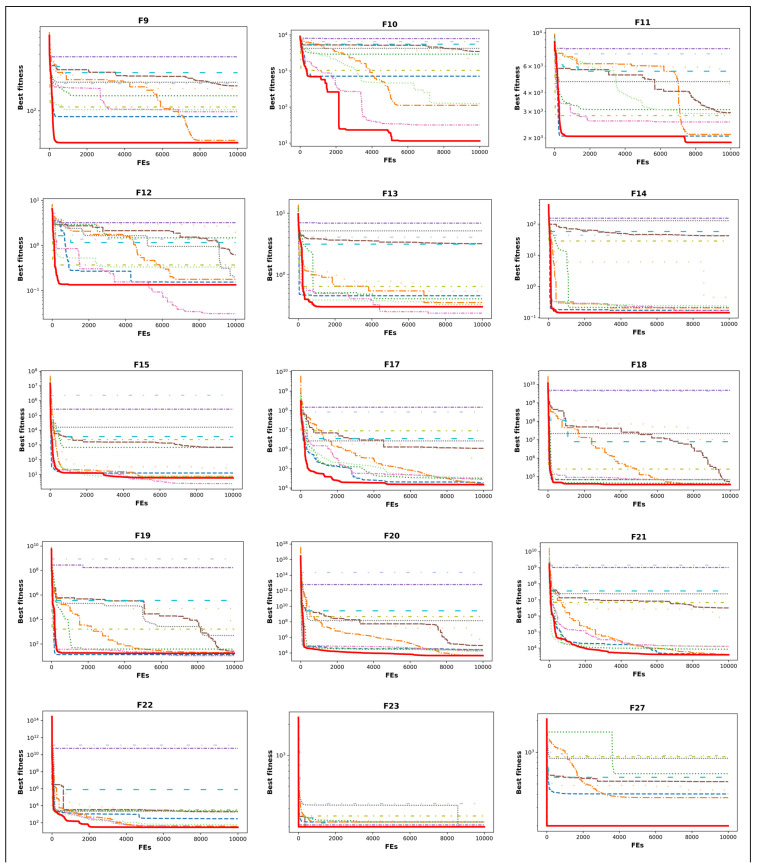

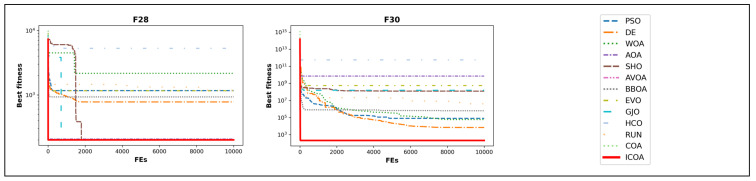
Convergence curves of ICOA and competing algorithms for CEC-2014 (D = 30).

In Figure 5 and Figure 6, the convergence curves of all algorithms for some test functions are given. In the performance analysis of MHAs, their behaviors of convergence are important criteria. The convergence behavior of an MHA gives information about the speed of the optimizer. The ICOA has better convergence in many cases. It indicates that the ICOA’s ability of exploration, exploitation, and transition between the two is well-developed. It should also avoid local minimum traps while solving different kinds of functions. Unimodal functions test the exploitation characteristics of MHAs. In terms of unimodal functions (Figure 5 and Figure 6: f1−f3), the ICOA has much better performance and does not converge early. Multimodal functions test the exploration capabilities of the optimizers. In multimodal functions (Figure 5 and Figure 6: f4−f16), the ICOA has a more vertical angle in general. This means that the ICOA’s ability of exploration is well-developed. This could be explained through the fact that the ICOA has an updated competition stage. Hybrid functions (Figure 5 and Figure 6: f17−f22) contain unimodal and multimodal functions in their structures. These functions test both the exploration and the exploitation abilities of MHAs. Once the convergence curves are studied, it is observed that the ICOA has the capacity to improve the results in low and high iteration numbers. Composition functions have multimodal characteristics. In these functions (Figure 5 and Figure 6: f23−f30), the ICOA performs a vertical descent. This means that the ICOA has a fast convergence ability. There are two main reasons for the ICOA having better exploration and exploitation abilities in low and high iteration numbers. The first one is that it has an updated competition stage. The second one is the iteration-dependent step length (V) and mapping factor (m). In the original version of the COA, while designing the competition stage, no stochastic component is used in the equation. Also, the model that is like the best one is used. This prevents the solutions made in this stage from being different from the solutions made in other stages. Thus, it blocks the optimizer from searching the solution space efficiently.

### 4.3. Ablation Study: Evidence of Component-Wise Improvements

In this section, the contributions of each improvement component proposed in the ICOA were analyzed individually. Within the purpose of presenting concrete proofs for the effectiveness of the improvements, an ablation study was carried out both in D = 10 and D = 30 dimensions by using eight sample CEC-2014 functions (F1, F4, F8, F11, F17, F20, F24, F27). These functions were selected to represent unimodal (F1), multimodal (F4, F8, F11), hybrid (F17, F20), and composition (F24, F27) categories. The tested algorithm variants are as follows. (1) COA (original): baseline algorithm, (2) COA + V: only adaptive step length (V) was added, (3) COA + m: only mapping vector (m) was added, (4) ICOA: has all the improvements (V+m). Table 23 presents the average results of 30 independent runs.

The results verify that each component enables meaningful improvements. The component of adaptive step length (V) enabled improvements varying between 5.0% and 77.1% depending on the functions. The component of mapping vector (m) generally had a more significant effect with improvements between 3.0% and 94.4%. The most remarkable results were in the F8, F17, and F20 functions, as the m component improved, respectively, 63.8%, 67.9%, and 94.4% in these functions.

The performance of the ICOA seems to be higher than the total of the individual components, which indicates a synergistic effect. For example, in function F1 (D=10), while the V component and m component enable, respectively, 23.5% and 38.5% improvement, the ICOA achieves a total improvement of 89.7%. Similarly, in function F17, while the V and m components enable, respectively, 28.3% and 67.9% improvement, the ICOA enables 94.9% improvement.

The runtime analysis indicates that the ICOA enables minimal computational cost and performance improvement. For 10,000 function evaluations, the ICOA requires only 1.0% additional runtime on average compared to the original COA (0.9% for D=10, 1.1% for D=30). This low computational cost supports the usability of the ICOA in practical applications. While the highest overhead is 2.7% for F20 function (D=30), the lowest is 0.1% for F20 function (D=10).

These ablation study results quantitatively verify that each improvement component proposed in the ICOA makes a significant contribution to the algorithm and that they generate a synergistic effect when they are used together. The fact that the individual contribution of the mapping vector (m) component is generally higher than the adaptive step length (V) component emphasizes the significance of the dimension selection strategy in high-dimensional problems.

### 4.4. Scalability Analysis

**Figure 7:**
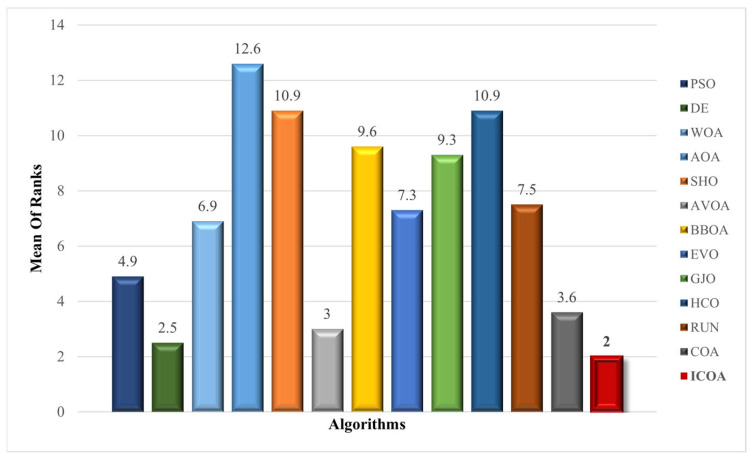
The average ranks of algorithms in different dimensions.

Scalability evaluation is used to assess whether the size of the problems affects the efficiency of the ICOA. An MHA is expected to present as efficient a performance on high-dimensional problems as on low-dimensional ones. In the scalability test, the ICOA was used to solve 10 scalable unimodal and multimodal functions of size ranging from 100 to 500 with a length of 50 steps. The FE was set to 10,000 and the results of 30 independent runs were recorded. For each dimension, the rank values of the algorithms from the Friedman test were calculated and averaged. These average rank values are given in Figure 7. The ICOA is the most successful algorithm with a score of 2. The results show that the ICOA is the least affected algorithm when the problem size increases. DE is the second most successful algorithm with a score of 2.5 and the AVOA is the third most successful algorithm with a score of 3. Table 24 shows the results for dimension 500. The ICOA was the most competitive optimizer in seven of the functions (f1−f3, f5−f7, f10)

### 4.5. Application of ICOA to Engineering Problems

In this subsection, the ICOA is applied to five engineering design problems to verify its validity. The ICOA and competing algorithms are run on the same computer. The results from 30 independent runs of the optimizers are saved. The number of search agents is set to 50 and the number of iterations to 500. The best, average, worst, and standard deviation values of the algorithms are used as comparison parameters. The convergence curves of the algorithms are also given. In engineering design problems, constraints need to be included in the calculation while solving the problem as well. Although there are many methods proposed in the literature, the penalty function is the most preferred method due to its simplicity and ease of use. The penalty function is also used in this study (Equation (18)).

(18) $$F(\vec{x})=f(\vec{x})\pm \left[\sum_{i=1}^{m}\;k_{i}\cdot max{\left(0,g_{i}(\vec{x})\right)}^{\gamma }+\sum_{j=1}^{n}\;l_{j}\cdot {\left|h_{j}(\vec{x})\right|}^{\delta }\right]$$

where F(z) is the modified objective function. $k_{i}$, $l_{j}$ are the positive penalty values. $g_{i}(\vec{x})$, $h_{j}(\vec{x})$ represent constraint functions and n,m are the number of constraint functions. γ, δ are constant numbers and usually have a value of 2. The task of the penalty function is to dramatically increase the penalty score whenever the optimizer violates any constraint. This increase helps the search agents to move towards probable spaces in the solution space where better solutions are.

#### 4.5.1. Cantilever Beam Design

**Figure 8:**
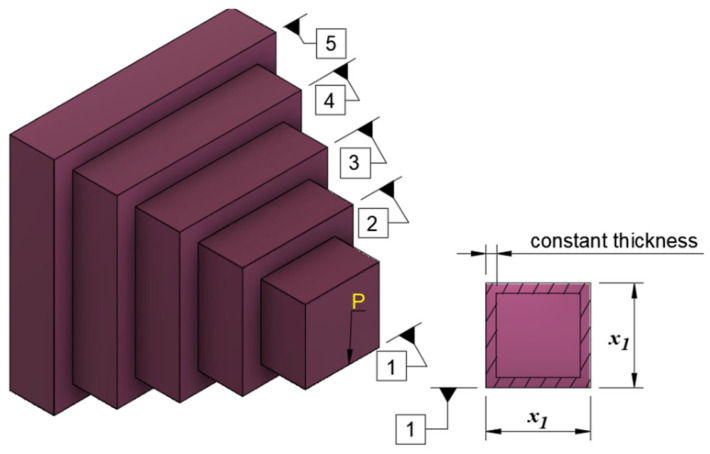
Cantilever beam design problem.

The aim of the cantilever beam design problem is to minimize the weight of the cantilever beam [49]. In the problem, there are five hollow beams with constant thickness. The schematic diagram of the problem is given in Figure 8. The length of each beam is different from the other one and these lengths are the design variants of the problem $x_{1},x_{2},x_{3},x_{4},x_{5}$. In addition, the problem has one constraint. The mathematical model of the problem is given below (Equations (19)–(22)).

(19) $$\mathrm{Consider}\;\mathrm{variable}:\;\vec{x}=\left[x_{1},x_{2},x_{3},x_{4},x_{5}\right]$$

(20) $$\mathrm{Minimize}:\;f_{1}(\vec{x})=0.0624\left(x_{1}+x_{2}+x_{3}+x_{4}+x_{5}\right)$$

(21) $$\mathrm{Subject}\;\mathrm{to}:\;g_{1}(\vec{x})=\frac{61}{x_{1}^{3}}+\frac{37}{x_{2}^{3}}+\frac{19}{x_{3}^{3}}+\frac{7}{x_{4}^{3}}+\frac{1}{x_{5}^{3}}-1\leq 0$$

(22) $$\mathrm{Variable}\;\mathrm{range}:\;0.01\leq x_{i}\leq 100,i=1,\ldots ,5$$

**Figure 9:**
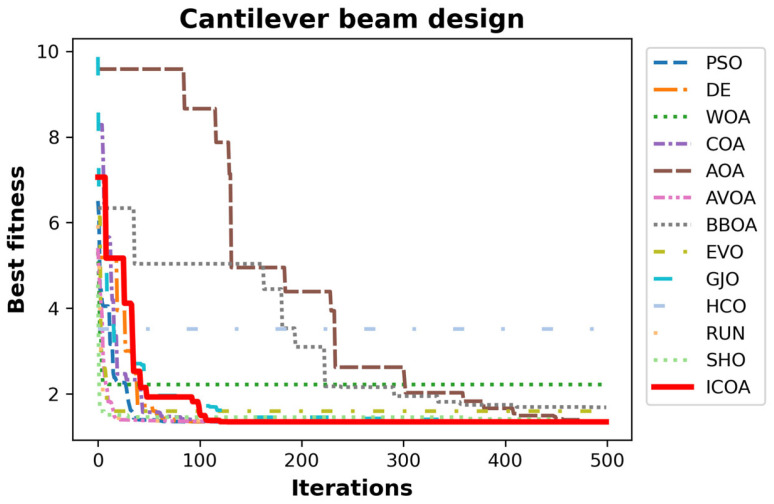
Convergence curves of ICOA and competing algorithms for cantilever beam design.

Table 25 indicates the best, average, worst, and standard deviation values of all the algorithms by the cantilever beam design problem. Studying Table 25, it is observed that the results of the ICOA are better than its competitors. The algorithms that stand out with their results are the ICOA, COA, AVOA, and DE. The superiority of the ICOA, especially in mean and standard deviation values, means that it can produce consistently successful results. Table 26 indicates the values of decision variables and optimal weights of all the algorithms. The ICOA was the optimizer with the best optimal weight ($f_{1}=1.339965$) with $\vec{x}=(6.00741,\;5.32034,\;4.49318,\;3.50262,\;2.15025)$ structure variable values. The convergence curves of all optimizers are given in Figure 9.

**Figure 10:**
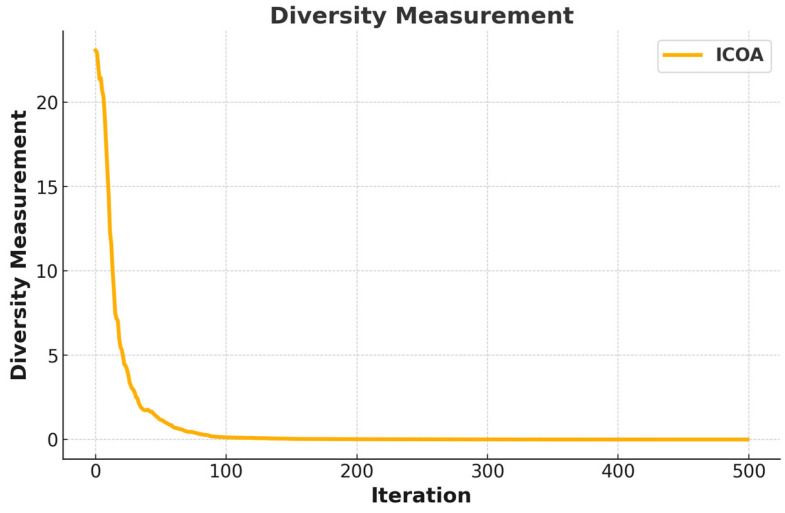
Population diversity of ICOA for cantilever beam design.

**Figure 11:**
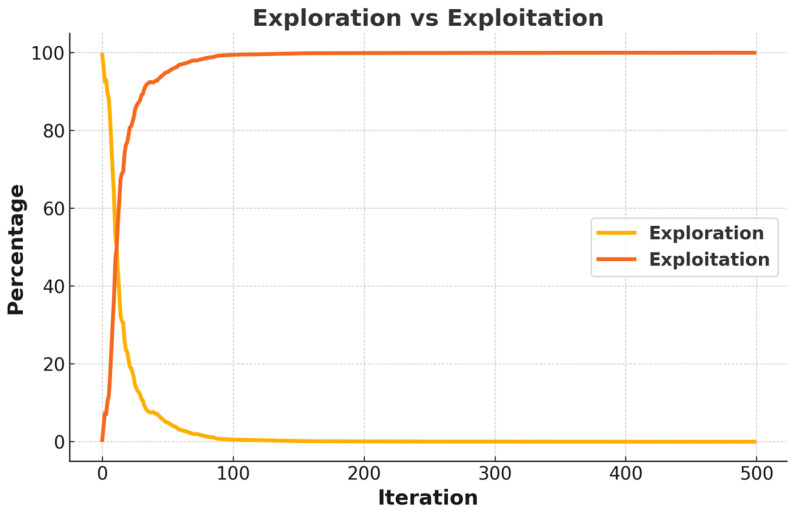
Exploration–exploitation balance of ICOA for cantilever beam design.

Figure 10 and Figure 11 present the analysis of the ICOA’s exploration and exploitation balance. This analysis is significant for understanding how the algorithm performs in engineering problems.

As indicated in Figure 10, the diversity measurement of the algorithm has a significant change over the iterations. With a high diversity value at the beginning, the algorithm tends to explore a large area of the solution space. This indicates that the algorithm has an effective exploration capability in its early stages. In the first 50 iterations, the diversity decreases rapidly, indicating that the algorithm starts to focus on promising regions. By the 100th iteration, the diversity almost approaches zero and the algorithm begins the full exploitation phase.

Figure 11 shows how the exploration and exploitation of the algorithm change over iterations in percentages. In the early iterations, the exploration starts at around 100% and decreases rapidly, reaching around 10% at the 50th iteration. On the other hand, the exploitation starts at zero and increases rapidly, reaching almost 100% by the 100th iteration. As can be observed through the graphics, the algorithm works in two distinct stages.

Exploration phase (iteration between 1 and 50): In this phase, the algorithm searches the different regions of the solution space and determines potential optimum points. High diversity reduces the risk of the algorithm being trapped by local optimums.

Exploitation Phase (iterations between 50 and 500): In this phase, the algorithm improves the solutions by concentrating on promising regions that are found in the exploration phase. A decrease in the diversity and an increase in the exploitation accelerate the convergence of the algorithm. This balance of exploration and exploitation directly affects the effectiveness of the algorithm in the engineering problem at hand. While the effective exploration at the beginning allows the algorithm to explore the solution space effectively, the intensive exploitation at the later stages ensures that the found solutions are optimized quickly. This balance is of critical importance, especially in complex engineering problems with constraints. While the high exploration capacity at the first stage enables the algorithm to explore the constraints and determine suitable solution regions, the exploitation at the later stages ensures rapid convergence for optimal solutions under certain constraints.

As a result, studying the exploration and exploitation balance of the ICOA, it is observed that the algorithm concentrates on exploration in the early stages but on exploitation in the later ones. This balance is one of the main factors for the algorithm to be successful in engineering design problems. It also allows the algorithm to have effective results in complicated and multimodal solution spaces.

#### 4.5.2. Gear Train Design

**Figure 12:**
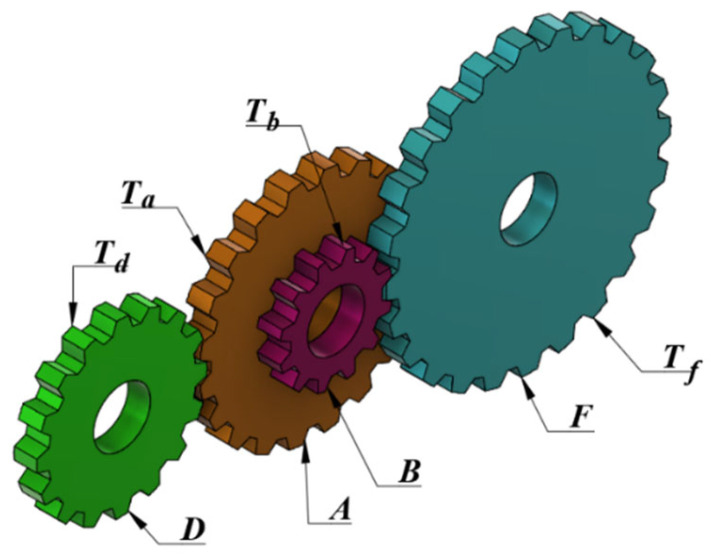
Gear train design problem.

There are four gear wheels in the gear train design problem. The numbers of teeth of these gears are the decision variables of the problem ($T_{a},T_{b},T_{d},T_{f}$). The schematic diagram of the problem is given in Figure 12. Since the decision variables are the numbers of teeth, it is important to present decision variables as integers. The aim of the gear train design problem is to minimize the gear ratio [54]. The mathematical model of the problem is as follows (Equations (23)–(25)).

(23) $$\mathrm{Consider}\;\mathrm{variable}:\;\vec{x}=\left[x_{1},x_{2},x_{3},x_{4}\right]=\left[T_{a},T_{b},T_{d},T_{f}\right]$$

(24) $$\mathrm{Minimize}:\;f_{2}(\vec{x})={\left(\frac{1}{6.931}-\frac{T_{b}\cdot T_{d}}{T_{a}\cdot T_{f}}\right)}^{2}$$

(25) $$\mathrm{Subject}\;\mathrm{to}\;(\mathrm{variable}\;\mathrm{range}):\;12\leq x_{1},x_{2},x_{3},x_{4}\leq 60$$

**Figure 13:**
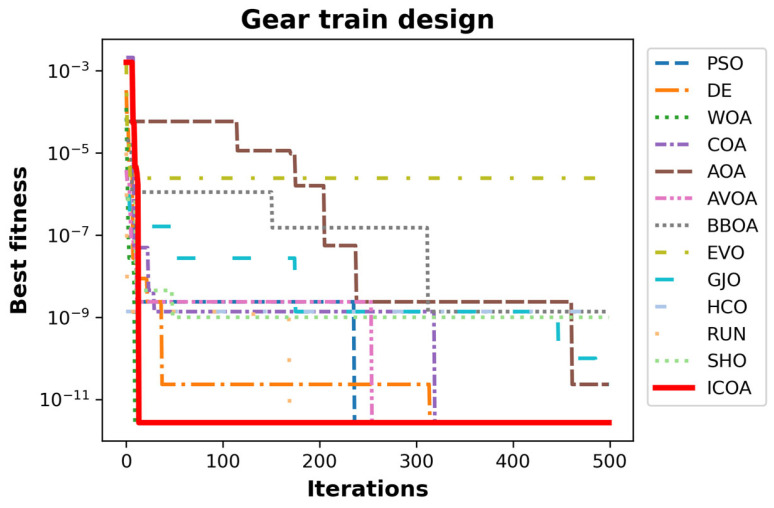
Convergence curves of ICOA and competing algorithms for gear train design.

Table 27 presents the best, average, worst, and standard deviation values of all the algorithms by the gear train design problem. The ICOA, COA, AVOA, AVOA, EVO, and RUN are the algorithms with the optimal cost. The ICOA and RUN are the most successful algorithms in the worst value metric. Table 28 gives the values of the decision variables and the optimal costs of all the algorithms. The ICOA is the optimizer with one of the best optimal costs $f_{2}=2.70086\times 10^{-12}$ with design parameters $\vec{x}=\left(43,\;16,\;19,\;49\right)$. The convergence curves of all optimizers are presented in Figure 13.

#### 4.5.3. Rolling Element Bearing Design

**Figure 14:**
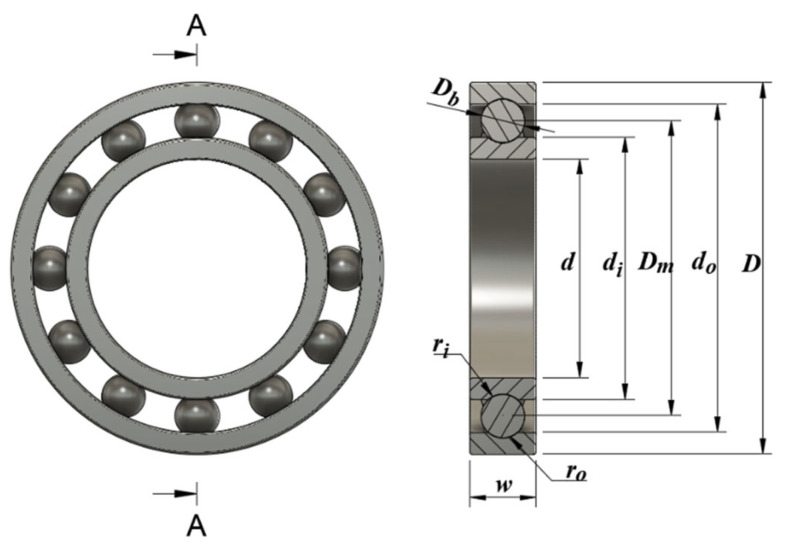
Rolling element bearing design problem.

The rolling element bearing design problem has 10 design variables and 10 constraints. The schematic diagram of the problem is given in Figure 14. The aim of the rolling element bearing design problem is to maximize the carrying capacity of the ball-bearing [55]. The mathematical model of the problem is as follows (Equations (26)–(30)).

(26) $$\mathrm{Consider}\;\mathrm{variable}:\;\vec{x}=\left[D_{m},D_{b},Z,f_{i},f_{o},K_{Dmin},K_{Dmax},\varepsilon ,e,\zeta \right]$$

(27) $$\mathrm{Maximize}:\;\left\{\begin{array}{lll} f_{3}(\vec{x})=f_{c}Z^{2/3}D_{b}^{1.8} & \;\mathrm{if}\;D_{b}\leq 25.4 & \;mm\\ f_{3}(\vec{x})=3.647f_{c}Z^{2/3}D_{b}^{1.4} & \mathrm{if}\;D_{b}>25.4\; & \;mm \end{array}\right.$$

(28) $$\mathrm{Subject}\;\mathrm{to}:\;g_{1}(\vec{x})=\frac{\phi_{o}}{2{sin}^{-1}\left(D_{b}/D_{m}\right)}-Z+1\geq 0,g_{2}(\vec{x})=2D_{b}-K_{Dmin}(D-d)\geq 0,\;g_{3}(\vec{x})=K_{Dmax}(D-d)-2D_{b}\geq 0,g_{4}(\vec{x})=D_{m}-(0.5-e)(D+d)\geq 0,\;g_{5}\left(\vec{x}\right)=\left(0.5+e\right)\left(D+d\right)-D_{m}\geq 0,g_{6}\left(\vec{x}\right)=D_{m}-0.5\left(D+d\right)\geq 0,\;g_{7}\left(\vec{x}\right)=0.5\left(D-D_{m}-D_{b}\right)-\varepsilon D_{b}\geq 0,g_{8}\left(\vec{x}\right)=\zeta B_{w}-D_{b}\leq 0,\;g_{9}(\vec{x})=f_{i}\geq 0.515,g_{10}(\vec{x})=f_{o}\geq 0.515$$

(29) $$\mathrm{Where}\;f_{c}=37.91{\left[1+{\left\{1.04{\left(\frac{1-\gamma }{1+\gamma }\right)}^{1.72}{\left(\frac{f_{i}\left(2f_{o}-1\right)}{f_{o}\left(2f_{i}-1\right)}\right)}^{0.4}\right\}}^{10/3}\right]}^{-0.3}\;\times \left(\frac{\gamma^{0.3}(1-\gamma {)}^{1.39}}{f_{o}(1+\gamma {)}^{\frac{1}{3}}}\right){\left(\frac{2f_{i}}{2f_{i}-1}\right)}^{0.41}\;\gamma =\frac{D_{b}}{D_{m}},f_{i}=\frac{r_{i}}{D_{b}},f_{o}=\frac{r_{o}}{D_{b}},\;\phi_{o}=2\pi -2{cos}^{-1}\frac{\{(D-d)/2-3(T/4){\}}^{2}+{\left\{D/2-(T/4)-D_{b}\right\}}^{2}-\{d/2+(T/4){\}}^{2}}{2\{(D-d)/2-3(T/4)\}\left\{D/2-(T/4)-D_{b}\right\}}\;T=D-d-2D_{b},D=160,d=90,B_{w}=30,r_{i}=r_{o}=11.033$$

(30) $$\mathrm{Variable}\;\mathrm{range}:\;0.5(D+d)\leq D_{m}\leq 0.6(D+d),0.15(D-d)\leq D_{b}\leq 0.45(D-d),\;4\leq Z\leq 50,0.515\leq f_{i}\leq 0.6,0.515\leq f_{o}\leq 0.6,0.4\leq K_{Dmin}\leq 0.5,\;0.6\leq K_{Dmax}\leq 0.7,0.3\leq \varepsilon \leq 0.4,0.02\leq e\leq 0.1,0.6\leq \zeta \leq 0.85$$

**Figure 15:**
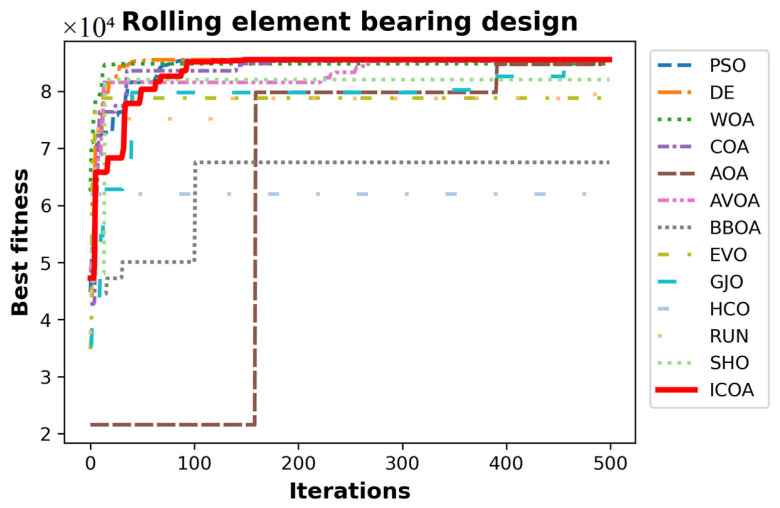
Convergence curves of ICOA and competing algorithms for rolling element bearing design.

Table 29 gives the best, average, worst, and standard deviation values of all the algorithms by the rolling element bearing design problem. The ICOA is the most competitive algorithm as it outperforms its competitors in the best $f_{3,best}=85,547.81075$ and average value $f_{3,ave}=85,498.37802$ metrics. These results mean that the ICOA could be successful in maximum problems as well. Table 30 presents the values of design variables and the optimal load-carrying capacity of all the algorithms. Figure 15 shows the convergence curves of all algorithms.

#### 4.5.4. Heat Exchanger Network Design

**Figure 16:**
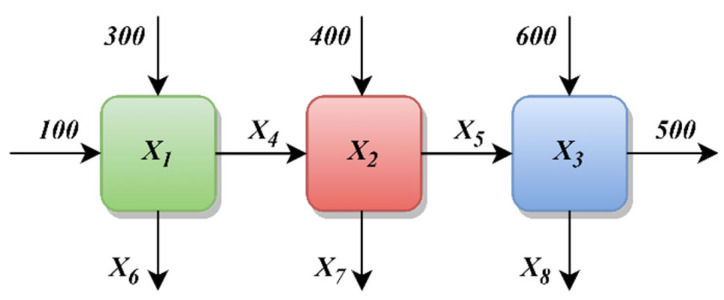
Heat exchanger network design problem.

The heat exchanger network design problem aims to design an optimal heat exchanger network to minimize the overall heat exchange area [56]. This system consists of three hot flow zones and one cold flow zone. The cold flow needs to be heated from 100 °F to 500 °F using the three hot flow zones. A schematic diagram of the problem is given in Figure 16. The problem has eight design variables and six constraints. The mathematical model of the problem is as follows (Equations (31)–(34)).

(31) $$\mathrm{Consider}\;\mathrm{variable}:\;\vec{x}=\left[x_{1},x_{2},x_{3},x_{4},x_{5},x_{6},x_{7},x_{8}\right]$$

(32) $$\mathrm{Minimize}:\;f_{4}(\vec{x})=x_{1}+x_{2}+x_{3}$$

(33) $$\mathrm{Subject}\;\mathrm{to}:\;g(1)=-1+0.0025\left(x_{4}+x_{6}\right)\leq 0\;g(2)=-1+0.0025\left(x_{5}+x_{7}-x_{4}\right)\leq 0\;g(3)=-1+0.01\left(x_{8}-x_{5}\right)\leq 0\;g(4)=-x_{1}x_{6}+833.33252x_{4}+100x_{1}-83333.333\leq 0\;g\left(5\right)=-x_{2}x7+1250x_{5}+x_{2}x_{4}-1250x_{4}\leq 0\;g(6)=-x_{3}x_{8}+1250000+x_{3}x_{5}-2500x_{5}\leq 0$$

(34) $$\mathrm{Variable}\;\mathrm{range}:\;100\leq x_{1}\leq 10000,1000\leq x_{2},x_{3}\leq 10000,\;10\leq x_{4},x_{5},x_{6},x_{7},x_{8}\leq 1000$$

**Figure 17:**
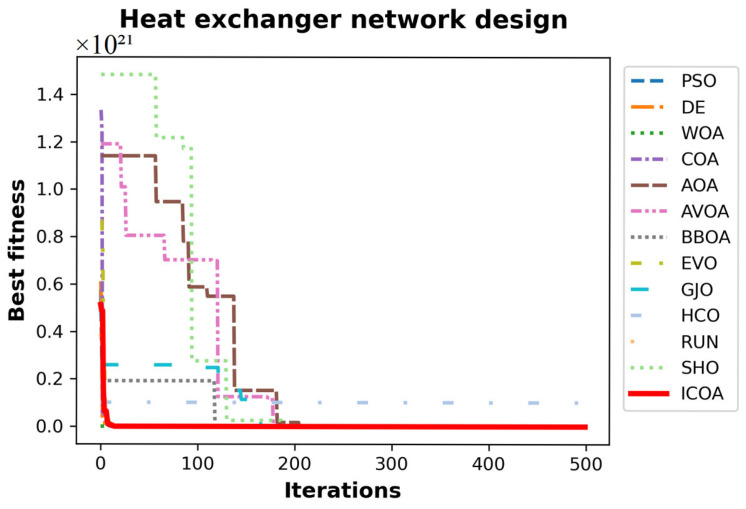
Convergence curves of ICOA and competing algorithms for heat exchanger network design.

Table 31 presents the best, average, worst, and standard deviation values of all the algorithms by the heat exchanger network design problem. The ICOA outperforms its competitors in all best, average, worst, and standard deviation comparison metrics. In the best value metric, more than one algorithm gives results close to the optimal result. The standard deviation of the ICOA is very low compared to its competitors. This means that the ICOA gives the optimal result in a consistent manner. Table 32 shows the values of the decision variables and the best results of all the algorithms. Figure 17 shows the convergence curves of all algorithms.

#### 4.5.5. Tabular Column Design

**Figure 18:**
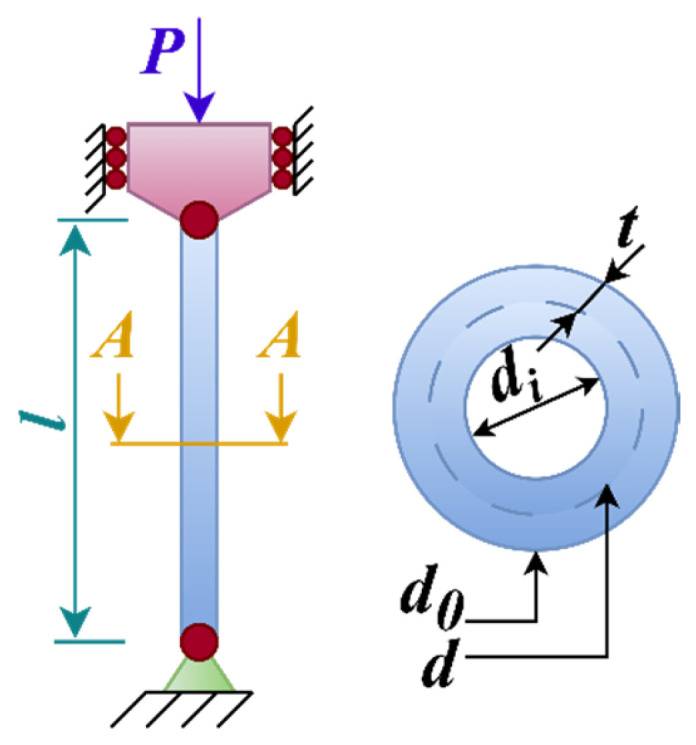
Tabular column design problem.

The tabular column design problem has two design variables and six constraints. The aim of the problem is to carry the compressive load on the column with minimum cost [57]. The load on the column stems from the construction and the material of which the column is made. The schematic diagram of the problem is given in Figure 18. The mathematical model of the problem is as follows (Equations (35)–(39)).

(35) $$\mathrm{Consider}\;\mathrm{variable}:\;\vec{x}=[d,t]=\left[x_{1},x_{2}\right]$$

(36) $$\mathrm{Minimize}:\;f_{5}(\vec{x})=9.82dt+2d$$

(37) $$\mathrm{Subject}\;\mathrm{to}:\;g_{1}(\vec{x})=\frac{x_{1}}{14}-1\leq 0,\;g_{2}(\vec{x})=\frac{8PL^{2}}{\pi 3Eh_{1}h_{2}\left(h_{1}^{2}+h_{2}^{2}\right)}-1\leq 0\;g_{3}(\vec{x})=\frac{2}{h_{1}}-1\leq 0,\;g_{4}(\vec{x})=\frac{P}{\pi h_{1}h_{2}\sigma_{y}}-1\leq 0\;g_{5}(\vec{x})=\frac{0.2}{x_{2}}-1\leq 0,\;g_{6}(\vec{x})=\frac{x_{2}}{0.8}-1\leq 0$$

(38) $$\mathrm{Where}\;P=2500,\sigma_{y}=500,E=0.85\times 10^{6},\sigma_{y}=500$$

(39) Variable range: 2≤d≤14,0.2≤t≤0.8

**Figure 19:**
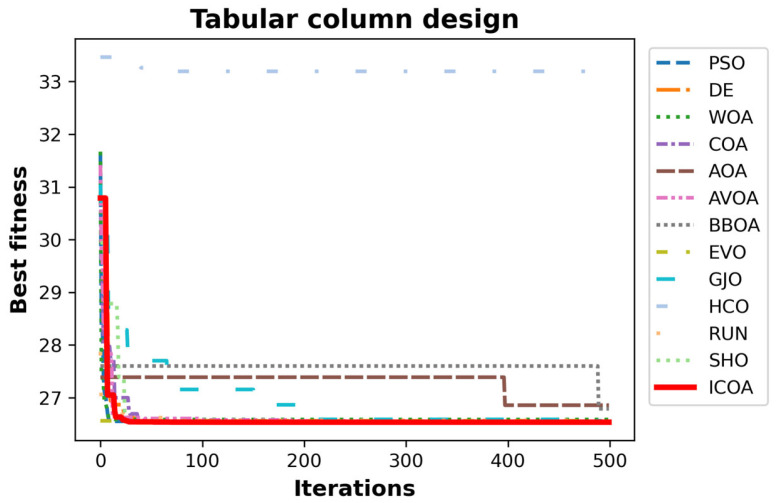
Convergence curves of ICOA and competing algorithms for tabular column design.

Table 33 gives the best, average, worst, and standard deviation values of all the algorithms by the tabular column design problem. Studying Table 33, it is observed that the results of the ICOA, COA, and AVOA are, specifically, very close to each other. In the best and average value metric, the ICOA outperforms its competitors with the scores of, respectively, $f_{5,best}=26.53132788$ and $f_{5,best}=26.531344$. The AVOA is more successful in the worst value metric. Studying the mean and standard deviation values of the ICOA, the ICOA could be interpreted as rarely producing its worst value. Table 34 gives the values of design variables and optimum costs of all the algorithms. Figure 19 shows the convergence curves of all optimizers.

### 4.6. Critical Assessment of ICOA Implementation

This section provides a comprehensive evaluation of the strengths and potential drawbacks associated with the proposed ICOA algorithm.

#### 4.6.1. Algorithmic Strengths

The ICOA’s most prominent advantage lies in its enhanced exploration–exploitation balance achieved through the adaptive step length mechanism. In CEC-2014 tests, it obtained the best average ranking (1.70) while the COA lagged behind (4.13). The mapping vector (m) mechanism enables more effective performance in high-dimensional problems, achieving the best results in seven out of 10 functions in 500-dimensional tests. Furthermore, the removal of the *X_shade_* parameter and introduction of stochastic components significantly reduced premature convergence, enhancing the algorithm’s ability to escape local optima traps.

#### 4.6.2. Limitations and Drawbacks

The ICOA’s primary limitation is increased computational complexity. Step length calculation, mapping vector generation, and permutation operations require 15–20% more execution time compared to the basic COA. The algorithm’s performance is critically dependent on r₁ and r₂ parameters, with optimal ranges of [0.2, 0.8] for r₁ and [0.3, 0.7] for r₂. Values outside these ranges significantly degrade performance.

The ICOA exhibits limited effectiveness in certain problem types. In highly constrained problems, frequent constraint violations may occur; its continuous nature makes it unsuitable for discrete optimization problems; and in low-dimensional problems, the overhead may exceed the benefits. Additionally, the algorithm lacks formal convergence proofs, and its stochastic nature complicates theoretical analysis.

#### 4.6.3. Performance Assessment

Experimental results demonstrate that the ICOA exhibits varying success levels depending on problem type. It achieves the highest success rate in unimodal functions, moderate performance in multimodal functions, and more limited advantages in composite functions. This indicates that the algorithm’s superiority diminishes with increasing problem complexity.

#### 4.6.4. Recommendations

The ICOA is ideal for high-dimensional continuous optimization problems, moderately constrained engineering problems, and situations requiring balanced exploration–exploitation. However, a cost–benefit analysis should be conducted for low-dimensional problems, the parameter tuning process should be carefully managed, and algorithm selection should be made according to problem type.

The ICOA offers significant improvements over the COA, particularly for high-dimensional problems. However, increased computational costs, parameter sensitivity, and limitations in certain problem types must be considered. This comprehensive evaluation emphasizes the importance of balanced consideration of the ICOA’s strengths and limitations for successful implementation.

## 5. ROAS Rate Problem

In this section, a Return on Advertising Spend (ROAS) problem based on real data is considered. The data belongs to the company “Makarna Lütfen” operating in Kırklareli, Turkey. The company sells its products on an online marketplace. The data given in Appendix A contains the statistics of this marketplace. The data covers May 2024. The firm designs advertising strategies to increase its sales. To begin with, the company categorizes its products into groups. This grouping strategy depends on the similarity of the products, the number of the sales, and the experience of the sales team. The company shared the data of six of the groups it formed. However, the company did not find it appropriate to name the products in the groups. In order to increase the number of the sales of each group, some keywords were made by the company officials. When these keywords are searched by users visiting the marketplace, the products in the relevant groups of the company are shown to the users. One keyword could be used for more than one group. The firm pays an advertising fee to the marketplace to show the company’s products when a keyword is searched by a user. Keywords can also be used by competitors. Therefore, advertising management is an active process. Company authorities set minimum and maximum spending limits for each keyword.

An optimization problem was generated using Advertising Spend (AS), Revenue (R), and ROAS data from the company. In the tables given in Appendix A, other data obtained from the company are given for researchers to make use of. ROAS is the ratio of advertising spend to revenue. It is found by dividing revenue by advertising expenses. The equations related to the ROAS rate problem are given below. Table 35 shows the keywords used in the advertising campaigns.

Minimize:

(40) $$f_{ROAS}=0.2\times {ROAS}_{1}+0.2\times {ROAS}_{2}+0.1\times {ROAS}_{3}+0.2\times {ROAS}_{4}+0.1\times {ROAS}_{5}+0.2\times {ROAS}_{6}$$

Subject to:

(41) $$g_{1}=\left(x_{1}+x_{2}+x_{3}+x_{4}+x_{5}+x_{6}\right)/32,000<1,\;g_{2}=\left(y_{1}+y_{2}+y_{3}+y_{4}+y_{5}+y_{6}\right)/9400<1\;g_{3}=\left(z_{1}+z_{2}+z_{3}+z_{4}+z_{5}+z_{6}\right)/7400<1,\;g_{4}=\left(a_{1}+a_{2}+a_{3}+a_{4}+a_{5}+a_{6}\right)/4950<1\;g_{5}=\left(b_{1}+b_{4}\right)/585<1,\;g_{6}=\left(c_{1}+c_{4}\right)/1200<1,\;g_{7}=\left(d_{1}+d_{2}+d_{4}+d_{5}+d_{6}\right)/7015<1\;g_{8}=\left(e_{1}+e_{2}+e_{5}\right)/2900<1,\;g_{9}=\left(f_{3}+f_{5}+f_{6}\right)/6300<1,\;g_{10}=\left(g_{5}+g_{6}\right)/300<1\;g_{11}=\left(h_{3}+h_{6}\right)/135<1,\;g_{12}=\left(p_{1}+p_{2}\right)/100<1,\;g_{13}=\left(w_{2}+w_{3}\right)/1250<1$$

where

(42) $${ROAS}_{1}=\frac{R_{1}}{{AS}_{1}}\;{R_{1}=14.57x}_{1}+2.76b_{1}+317.14c_{1}+2.69a_{1}+0.26y_{1}+44.59z_{1}+27.93d_{1}+8.36e_{1}+13.95p_{1}\;{AS}_{1}=x_{1}+b_{1}+c_{1}+a_{1}+y_{1}+z_{1}+d_{1}+e_{1}+p_{1}\;{ROAS}_{2}=\frac{R_{2}}{{AS}_{2}}\;{R_{2}=30.95x}_{2}+13.02d_{2}+{6.25y}_{2}+14.73e_{2}+{16.07p}_{2}+{194.79w}_{2}+{6.69z}_{2}+{5622.52a}_{2}\;{AS}_{2}=x_{2}+d_{2}+y_{2}+e_{2}+p_{2}+w_{2}+z_{2}+a_{2}\;{ROAS}_{3}=\frac{R_{3}}{{AS}_{3}}\;R_{3}={1.03w}_{3}+{117.3x}_{3}+{4.55a}_{3}+{0.73y}_{3}+198.02h_{3}+{473.83f}_{3}+{8.04z}_{3}\;{AS}_{3}=w_{3}+x_{3}+a_{3}+y_{3}+h_{3}+f_{3}+z_{3}\;{ROAS}_{4}=\frac{R_{4}}{{AS}_{4}}\;R_{4}={30.38x}_{4}+{4.84z}_{4}+{0.79y}_{4}+{79.31a}_{4}+{1.3c}_{4}+{30.7b}_{4}+{13.9d}_{4}\;{AS}_{4}=x_{4}+z_{4}+y_{4}+a_{4}+c_{4}+b_{4}+d_{4}\;{ROAS}_{5}=\frac{R_{5}}{{AS}_{5}}\;R_{5}={102.52x}_{5}+{7.15y}_{5}+{8.02z}_{5}+{7.45d}_{5}+{6.1e}_{5}+{0.77a}_{5}+{0.79f}_{5}+{16.45g}_{5}\;{AS}_{5}=x_{5}+y_{5}+z_{5}+d_{5}+e_{5}+a_{5}+f_{5}+g_{5}\;{ROAS}_{6}=\frac{R_{6}}{{AS}_{6}}\;R_{6}={31.68x}_{6}+{4.68g}_{6}+{41.24a}_{6}+{71.76y}_{6}+{4.94z}_{6}+{231.84d}_{6}+{3.11f}_{6}+{37.46h}_{6}\;{AS}_{6}=x_{6}+g_{6}+a_{6}+y_{6}+z_{6}+d_{6}+f_{6}+h_{6}$$

Variable range:

(43) $$\begin{array}{l} 4000<x<6000,\;1500<y<1600,\;1000<z<1350,\;775<a<850,\;125<b<375\\ 450<c<675,\;1370<d<1420,\;920<e<980,\;1650<f<2300,\;100<g<175,\;25<h<85\\ 35<p<55,\;350<w<750 \end{array}$$

In this section, we present the ICOA, COA, as well as the up-to-date and validated Artificial Protozoa Optimizer (APO) [58], Flood Algorithm (FLA) [59], Greylag Goose Optimization (GGO) [60], Hippopotamus Optimization (HO) [61], Hiking Optimization Algorithm (HOA) [62], Catch Fish Optimization Algorithm (CFOA) [63], Electric Eel Foraging Optimization (EEFO) [64], and Puma optimizer (PO) [65].

**Figure 20:**
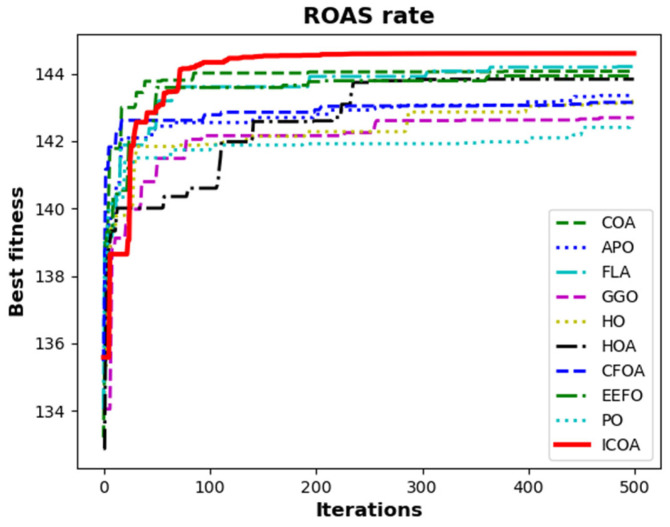
Convergence curves of ICOA and competing algorithms for ROAS problem.

Table 36 presents the results of all algorithms for the ROAS rate problem. The rows of the table show the total AS for each keyword. The row labeled as the total indicates the total AS. The last row presents the ROAS rate of the algorithms. The ICOA is the most successful algorithm with a score of 144.601. The FLA is the second algorithm with a score of 144.211 while the COA is the third algorithm with a score of 144.072. Figure 20 shows the convergence curves of the algorithms. All these results show that the ICOA could successfully solve real-world problems. It also indicates that the ICOA is efficient in multi-objective problems.

## 6. Discussion

The most important practical contribution of the ICOA is seen in its success in real-world ROAS (Return on Advertising Spend) optimization problems. This optimization study, using the May 2024 advertisement data of the company called ‘Makarna Lütfen’ operating in Kırklareli, emphasizes the value of the ICOA in industrial applications. The problem requires 13 keywords (Makarna Lütfen, Pasta, Baby Biscuits, Organic, Baby Semolina, etc.) and an optimal distribution of advertisement budget among six product groups. With its 144.601 performance score, the ICOA has better results than its closest competitor, the FLA, having a score of 144.211. This improvement stands for almost USD 86 additional efficiency and enables USD 1.032 income annually. This result is crucial for small- and medium-sized e-commerce businesses; for a company with a monthly advertising budget of USD 100,000, a 0.27% improvement generates an additional annual return of USD 3240. The 78 decision variables of the ROAS problem and its having 13 constraints verify that the ICOA is successfully able to process real-world complications. The algorithm’s ability of automatic parameter adjustment could be easily integrated into daily optimization processes in digital marketing agencies. The results in engineering applications are also supportive; the 0.0007% weight reduction in cantilever beam design and 0.09% capacity increase in bearing design indicate the applicability of the ICOA in various technical areas. These results suggest that the ICOA could generate concrete economic value in real industrial problems, beyond its academic success.

## 7. Conclusions

In this study, an improved version of the Crayfish Optimization Algorithm (COA) is presented. The proposed algorithm is named the Improved Crayfish Optimization Algorithm (ICOA). The COA consists of a summer resort stage (exploration), competition stage (exploitation), and foraging stage (exploitation). In the competition stage, crayfish compete with other crayfish to enter the caves. In the competition stage, while modeling this competition, only the distance between the crayfish is taken into account. In the ICOA, a new model is proposed by including step lengths and mapping vectors into the mathematical model in addition to the distances of the crayfish. In order to verify the validity of the ICOA, it is compared to 12 MHAs. The CEC-2014 dataset with 30 complex test functions is used for the comparison. The results of the optimizers are interpreted with a Wilcoxon Signed-Rank Test. Moreover, five engineering design problems and a Return on Advertising Spend (ROAS) problem were used to compare the algorithms.

The results of this study are as follows.

- In the competition stage of the ICOA, step length is inversely proportional to iteration. This enables the competition stage to help exploration at low iteration numbers and exploitation at high iteration numbers.
- The randomness of the mapping vector increases the stochastic property of the ICOA.
- CEC-2014 test results indicate that the ICOA is superior to its competitor algorithms. It is a significant result that the ICOA’s efficiency does not decrease, especially when the dimension of the problem increases.
- By means of the mapping vector, in high-dimensional problems, updating only some dimensions at a time both reduces the computational load of the algorithm and controls excessive randomness.
- Studying convergence curves, it is observed that the ICOA has a better curve than the COA. This verifies that the improvements made in the competition stage are successful. The number one reason why the ICOA has a better slope is that it performs exploration at low iteration numbers and exploitation at high iteration numbers. The second reason is the mapping vector of the ICOA.
- Radar graphics indicate that the ICOA has more consistent results than its competitors.
- Scalability analysis indicates that the ICOA is the optimizer that is the least affected algorithm by the dimension increase.
- The results of engineering design problems provide the promising impression that the ICOA could solve real-world problems.
- The ICOA’s success in the ROAS problem heralds that it could solve complex real-world problems. The reason for the ICOA’s success in this problem is the improvements made in the competition stage. Removing the $X_{shade}$ parameter prevents the ICOA from being caught by the local minimum trap.

Despite its success, the ICOA still has certain limitations. The performance of the algorithm may be sensitive to the tuning of some parameters such as the size of the mapping vector and the adaptation rate of the step length. Although the scalability tests seem to be promising, further evaluation of the algorithm’s effectiveness on large-scale, highly constrained or discrete optimization problems could be beneficial.

Future studies could include hybridizing the ICOA with other meta-heuristic or machine learning-based methods or improving its performance. It is possible for parameter control mechanisms to be improved through meta-learning or reinforcement learning approaches. Extending the ICOA for multi-objective and constrained optimization problems also appears promising

The flexible and adaptive structure of the ICOA makes it suitable for a wide range of practical applications. Potential application areas could be structural optimization, parameter tuning in machine learning, optimization of energy systems, supply chain and logistics management, biomedical engineering, and financial portfolio optimization. The successful practical application of the ICOA to the ROAS problem points out its promise for real-world data-driven optimization tasks.

## Figures and Tables

**Table 1:** Parameter settings of ICOA and competing algorithms.

Algorithms	Parameter Settings
PSO	$w=[0.9,\;0.2],\;c_{1}=2,\;c_{2}=2$
DE	$p_{CR}=0.5,\;\beta_{min}=0.1,\;\beta_{max}=0.9$
WOA	Probability of encircling mechanism=0.5, spiral factor=1
AOA	α=5, μ=0.5
AVOA	$L_{1}=0.8,\;L_{2}=0.2,\;w=2.5,\;P_{1}=0.6,\;P_{2}=0.4,\;P_{3}=0.6\;$
GJO	$C_{1}=1.5$
SHO	$r_{1}=0,\;r_{2}=0.1$
EVO	−
HCO	$P_{fit}=0.65,\;\lambda =\left[0,\;1\right],w=0.1,A_{1}\;and\;A_{2}=[2,\;4]\;$
RUN	a=20, b=12
BBOA	−
COA	$C_{1}=0.2,\;C_{3}=3,\;\mu =25,\;\sigma =3$
ICOA	$C_{1}=0.2,\;C_{3}=3,\;\mu =25,\;\sigma =3$

**Table 2:** The results of ICOA and competitor algorithms for CEC-2014 unimodal functions (D=10).

Func.	Index	Algorithms												
		PSO	DE	WOA	AOA	SHO	AVOA	BBOA	EVO	GJO	HCO	RUN	COA	ICOA
F1	Min	1.34 × 10^1^	8.78 × 10^0^	1.34 × 10^1^	4.93 × 10^7^	2.20 × 10^6^	1.66 × 10^3^	2.23 × 10^5^	3.45 × 10^6^	5.77 × 10^6^	9.47 × 10^7^	1.33 × 10^5^	1.46 × 10^3^	***3.59 × 10^0^***
	Ave	3.02 × 10^3^	2.28 × 10^3^	3.47 × 10^6^	3.47 × 10^8^	8.95 × 10^6^	1.51 × 10^4^	4.36 × 10^6^	2.06 × 10^7^	1.21 × 10^7^	3.27 × 10^8^	6.41 × 10^5^	1.39 × 10^4^	***1.44 × 10^3^***
	Std	4.15 × 10^3^	2.37 × 10^3^	1.00 × 10^7^	2.12 × 10^8^	4.40 × 10^6^	8.14 × 10^3^	4.96 × 10^6^	1.45 × 10^7^	3.19 × 10^6^	1.89 × 10^8^	4.80 × 10^5^	1.15 × 10^4^	***9.64 × 10^2^***
F2	Min	5.23 × 10^−2^	5.95 × 10^−4^	1.07 × 10^1^	7.25 × 10^9^	5.72 × 10^1^	2.24 × 10^1^	1.67 × 10^8^	3.41 × 10^7^	6.51 × 10^8^	4.74 × 10^9^	2.61 × 10^5^	1.60 × 10^−1^	***1.99 × 10^−11^***
	Ave	2.54 × 10^2^	2.07 × 10^0^	3.35 × 10^3^	1.04 × 10^10^	4.71 × 10^8^	3.27 × 10^3^	1.63 × 10^9^	6.61 × 10^8^	1.67 × 10^9^	1.08 × 10^10^	1.80 × 10^7^	1.67 × 10^3^	***1.91 × 10^−1^***
	Std	7.45 × 10^2^	5.85 × 10^0^	3.99 × 10^3^	2.13 × 10^9^	5.75 × 10^8^	3.86 × 10^3^	1.06 × 10^9^	4.73 × 10^8^	4.19 × 10^8^	3.51 × 10^9^	1.13 × 10^7^	2.57 × 10^3^	***9.33 × 10^−1^***
F3	Min	1.20 × 10^−7^	3.66 × 10^−9^	1.23 × 10^2^	2.04 × 10^4^	1.31 × 10^3^	1.86 × 10^1^	4.85 × 10^2^	9.40 × 10^2^	2.87 × 10^3^	5.96 × 10^4^	2.01 × 10^2^	3.20 × 10^−1^	***2.66× 10^−13^***
	Ave	1.53 × 10^−1^	1.09 × 10^−3^	9.06 × 10^3^	2.34 × 10^6^	4.04 × 10^3^	2.77 × 10^2^	3.65 × 10^3^	1.60 × 10^4^	1.02 × 10^4^	1.56 × 10^6^	6.69 × 10^2^	1.52 × 10^1^	***6.65 × 10^−4^***
	Std	4.60 × 10^−1^	2.40 × 10^−3^	8.86 × 10^3^	2.93 × 10^6^	2.23 × 10^3^	1.74 × 10^2^	2.35 × 10^3^	1.79 × 10^4^	3.51 × 10^3^	4.77 × 10^6^	3.28 × 10^2^	4.04 × 10^1^	***1.97 × 10^−3^***

**Table 3:** The results of ICOA and competitor algorithms for CEC-2014 multimodal functions (D=10).

Func.	Index	Algorithms												
		PSO	DE	WOA	AOA	SHO	AVOA	BBOA	EVO	GJO	HCO	RUN	COA	ICOA
F4	Min	4.76 × 10^−2^	1.56 × 10^−2^	1.44 × 10^−2^	5.82 × 10^2^	1.89 × 10^0^	1.22 × 10^−2^	4.98 × 10^1^	2.94 × 10^1^	4.29 × 10^1^	2.73 × 10^2^	1.11 × 10^0^	3.33 × 10^−2^	***1.10 × 10^−2^***
	Ave	2.47 × 10^1^	2.93 × 10^1^	2.87 × 10^1^	2.18 × 10^3^	5.74 × 10^1^	1.90 × 10^1^	1.86 × 10^2^	1.37 × 10^2^	6.31 × 10^1^	1.24 × 10^3^	2.26 × 10^1^	1.93 × 10^1^	***1.83 × 10^1^***
	Std	1.71 × 10^1^	***1.22 × 10^1^***	3.29 × 10^1^	1.16 × 10^3^	3.30 × 10^1^	1.72 × 10^1^	1.24 × 10^2^	7.67 × 10^1^	1.83 × 10^1^	5.92 × 10^2^	1.52 × 10^1^	1.69 × 10^1^	1.68 × 10^1^
F5	Min	2.00 × 10^1^	2.01 × 10^1^	2.00 × 10^1^	2.05 × 10^1^	2.00 × 10^1^	2.00 × 10^1^	1.60 × 10^1^	2.00 × 10^1^	2.02 × 10^1^	2.01 × 10^1^	***8.11 × 10^0^***	1.99 × 10^1^	2.00 × 10^1^
	Ave	2.01 × 10^1^	2.02 × 10^1^	2.01 × 10^1^	2.10 × 10^1^	2.00 × 10^1^	2.00 × 10^1^	1.99 × 10^1^	2.03 × 10^1^	2.03 × 10^1^	2.03 × 10^1^	***1.99 × 10^1^***	2.00 × 10^1^	2.00 × 10^1^
	Std	9.70 × 10^−2^	5.48 × 10^−2^	1.26 × 10^−1^	1.68 × 10^−1^	4.56 × 10^−2^	3.23 × 10^−2^	7.45 × 10^−1^	3.32 × 10^−1^	7.02 × 10^−2^	1.15 × 10^−1^	2.22 × 10^0^	1.37 × 10^−2^	***5.31 × 10^−3^***
F6	Min	8.86 × 10^−1^	3.26 × 10^−1^	6.64 × 10^0^	1.00 × 10^1^	3.03 × 10^0^	9.03 × 10^−1^	2.96 × 10^0^	1.11 × 10^0^	5.50 × 10^0^	4.63 × 10^0^	1.86 × 10^0^	2.49 × 10^−3^	***8.44 × 10^−4^***
	Ave	4.11 × 10^0^	2.55 × 10^0^	9.04 × 10^0^	1.22 × 10^1^	5.10 × 10^0^	3.47 × 10^0^	5.40 × 10^0^	3.85 × 10^0^	6.62 × 10^0^	8.13 × 10^0^	3.55 × 10^0^	3.63 × 10^0^	***2.27 × 10^0^***
	Std	1.40 × 10^0^	1.50 × 10^0^	1.23 × 10^0^	9.29 × 10^−1^	1.23 × 10^0^	1.53 × 10^0^	1.14 × 10^0^	1.62 × 10^0^	***8.47 × 10^−1^***	1.85 × 10^0^	8.85 × 10^−1^	2.06 × 10^0^	1.50 × 10^0^
F7	Min	9.11 × 10^−2^	3.94 × 10^−2^	1.19 × 10^0^	7.65 × 10^1^	1.21 × 10^0^	2.46 × 10^−2^	3.07 × 10^0^	1.47 × 10^0^	7.17 × 10^0^	6.30 × 10^0^	8.28 × 10^−1^	2.84 × 10^−2^	***9.86 × 10^−3^***
	Ave	2.52 × 10^−1^	1.50 × 10^−1^	9.50 × 10^0^	1.89 × 10^2^	1.20 × 10^1^	6.88 × 10^−2^	3.74 × 10^1^	1.25 × 10^1^	1.34 × 10^1^	3.56 × 10^1^	1.38 × 10^0^	7.44 × 10^−2^	***5.16 × 10^−2^***
	Std	1.81 × 10^−1^	7.52 × 10^−2^	6.08 × 10^0^	4.88 × 10^1^	1.34 × 10^1^	2.78 × 10^−2^	2.78 × 10^1^	8.45 × 10^0^	4.39 × 10^0^	1.83 × 10^1^	2.65 × 10^−1^	3.59 × 10^−2^	***2.32 × 10^−2^***
F8	Min	5.97 × 10^0^	0.00 × 10^0^	9.95 × 10^0^	8.54 × 10^1^	1.59 × 10^1^	***0.00 × 10^0^***	1.50 × 10^1^	4.38 × 10^0^	3.35 × 10^1^	1.81 × 10^1^	2.03 × 10^0^	1.29 × 10^−9^	***0.00 × 10^0^***
	Ave	1.85 × 10^1^	2.17 × 10^0^	4.07 × 10^1^	1.25 × 10^2^	2.56 × 10^1^	1.61 × 10^0^	2.72 × 10^1^	1.15 × 10^1^	4.06 × 10^1^	3.99 × 10^1^	1.21 × 10^1^	5.35 × 10^0^	***1.16 × 10^0^***
	Std	8.27 × 10^0^	1.28 × 10^0^	1.77 × 10^1^	1.57 × 10^1^	7.59 × 10^0^	9.25 × 10^−1^	7.37 × 10^0^	4.00 × 10^0^	4.47 × 10^0^	1.30 × 10^1^	4.34 × 10^0^	7.85 × 10^0^	***8.28 × 10^−1^***
F9	Min	1.09 × 10^1^	2.08 × 10^0^	1.59 × 10^1^	6.99 × 10^1^	2.31 × 10^1^	9.95 × 10^0^	1.91 × 10^1^	8.78 × 10^0^	3.69 × 10^1^	1.81 × 10^1^	8.25 × 10^0^	6.98 × 10^0^	***1.99 × 10^0^***
	Ave	2.56 × 10^1^	1.39 × 10^1^	5.02 × 10^1^	9.37 × 10^1^	3.41 × 10^1^	2.36 × 10^1^	2.91 × 10^1^	1.93 × 10^1^	4.93 × 10^1^	3.52 × 10^1^	1.92 × 10^1^	3.16 × 10^1^	***1.19 × 10^1^***
	Std	8.74 × 10^0^	7.06 × 10^0^	1.73 × 10^1^	8.99 × 10^0^	7.83 × 10^0^	7.86 × 10^0^	5.46 × 10^0^	6.33 × 10^0^	***3.81 × 10^0^***	1.46 × 10^1^	6.14 × 10^0^	1.64 × 10^1^	5.43 × 10^0^
F10	Min	7.20 × 10^0^	2.72 × 10^1^	3.01 × 10^2^	1.09 × 10^3^	1.32 × 10^2^	1.03 × 10^1^	4.12 × 10^2^	1.18 × 10^1^	3.80 × 10^2^	1.16 × 10^3^	7.23 × 10^0^	***2.50 × 10^−1^***	1.30 × 10^2^
	Ave	1.39 × 10^2^	***5.80 × 10^1^***	6.95 × 10^2^	1.65 × 10^3^	4.37 × 10^2^	7.82 × 10^1^	7.36 × 10^2^	2.23 × 10^2^	7.26 × 10^2^	1.64 × 10^3^	2.34 × 10^2^	4.22 × 10^2^	9.67 × 10^2^
	Std	9.15 × 10^1^	2.57 × 10^1^	2.50 × 10^2^	2.27 × 10^2^	1.43 × 10^2^	***2.53 × 10^1^***	2.02 × 10^2^	1.68 × 10^2^	1.46 × 10^2^	2.40 × 10^2^	1.62 × 10^2^	3.24 × 10^2^	3.51 × 10^2^
F11	Min	5.69 × 10^1^	5.49 × 10^1^	2.45 × 10^2^	1.78 × 10^3^	2.70 × 10^2^	1.43 × 10^2^	1.15 × 10^2^	2.38 × 10^2^	7.16 × 10^2^	9.45 × 10^2^	3.34 × 10^2^	1.29 × 10^2^	***5.22 × 10^1^***
	Ave	4.53 × 10^2^	3.75 × 10^2^	1.13 × 10^3^	2.28 × 10^3^	6.61 × 10^2^	6.40 × 10^2^	7.64 × 10^2^	8.08 × 10^2^	1.13 × 10^3^	1.50 × 10^3^	5.99 × 10^2^	5.19 × 10^2^	***3.61 × 10^2^***
	Std	2.16 × 10^2^	1.48 × 10^2^	3.48 × 10^2^	2.61 × 10^2^	2.04 × 10^2^	2.40 × 10^2^	2.72 × 10^2^	2.34 × 10^2^	***1.59 × 10^2^***	2.80 × 10^2^	1.64 × 10^2^	2.20 × 10^2^	1.85 × 10^2^
F12	Min	2.29 × 10^−2^	4.32 × 10^−2^	3.91 × 10^−1^	1.88 × 10^0^	5.16 × 10^−2^	***2.26 × 10^−4^***	1.15 × 10^−1^	7.44 × 10^−2^	4.15 × 10^−1^	6.89 × 10^−1^	3.42 × 10^−1^	4.92 × 10^−2^	4.01 × 10^−2^
	Ave	1.43 × 10^−1^	2.17 × 10^−1^	8.38 × 10^−1^	4.04 × 10^0^	2.36 × 10^−1^	***6.93 × 10^−2^***	4.06 × 10^−1^	3.34 × 10^−1^	7.39 × 10^−1^	1.45 × 10^0^	8.19 × 10^−1^	3.05 × 10^−1^	1.55 × 10^−1^
	Std	9.34 × 10^−2^	1.33 × 10^−1^	2.47 × 10^−1^	1.16 × 10^0^	1.35 × 10^−1^	***5.31 × 10^−2^***	1.57 × 10^−1^	1.92 × 10^−1^	1.95 × 10^−1^	4.31 × 10^−1^	2.00 × 10^−1^	1.83 × 10^−1^	9.60 × 10^−2^
F13	Min	1.72 × 10^−1^	4.15 × 10^−2^	2.73 × 10^−1^	2.07 × 10^0^	3.77 × 10^−1^	3.26 × 10^−2^	3.26 × 10^−1^	2.30 × 10^−1^	3.96 × 10^−1^	1.64 × 10^−1^	1.31 × 10^−1^	9.67 × 10^−2^	***2.49 × 10^−2^***
	Ave	4.61 × 10^−1^	1.55 × 10^−1^	6.59 × 10^−1^	4.83 × 10^0^	7.33 × 10^−1^	1.05 × 10^−1^	1.41 × 10^0^	4.39 × 10^−1^	7.01 × 10^−1^	1.19 × 10^0^	2.64 × 10^−1^	1.62 × 10^−1^	***8.43 × 10^−2^***
	Std	2.09 × 10^−1^	6.28 × 10^−2^	2.37 × 10^−1^	1.07 × 10^0^	3.98 × 10^−1^	***4.01 × 10^−2^***	9.51 × 10^−1^	1.34 × 10^−1^	1.10 × 10^−1^	8.64 × 10^−1^	7.27 × 10^−2^	5.97 × 10^−2^	4.10 × 10^−2^
F14	Min	4.02 × 10^−2^	***3.12 × 10^−2^***	1.04 × 10^−1^	2.47 × 10^1^	2.26 × 10^−1^	3.90 × 10^−2^	1.04 × 10^0^	2.38 × 10^−1^	9.88 × 10^−1^	3.04 × 10^−1^	8.05 × 10^−2^	6.85 × 10^−2^	5.30 × 10^−2^
	Ave	2.20 × 10^−1^	***1.08 × 10^−1^***	4.58 × 10^−1^	3.98 × 10^1^	1.54 × 10^0^	1.17 × 10^−1^	1.06 × 10^1^	2.55 × 10^0^	3.14 × 10^0^	7.90 × 10^0^	1.76 × 10^−1^	2.01 × 10^−1^	1.56 × 10^−1^
	Std	1.59 × 10^−1^	4.43 × 10^−2^	3.64 × 10^−1^	7.46 × 10^0^	2.41 × 10^0^	***4.03 × 10^−2^***	7.33 × 10^0^	2.18 × 10^0^	9.37 × 10^−1^	4.33 × 10^0^	4.36 × 10^−2^	8.29 × 10^−2^	6.57 × 10^−2^
F15	Min	7.14 × 10^−1^	5.68 × 10^−1^	3.24 × 10^0^	1.14 × 10^3^	1.78 × 10^0^	5.19 × 10^−1^	2.77 × 10^0^	2.68 × 10^0^	6.49 × 10^0^	1.13 × 10^4^	1.84 × 10^0^	4.30 × 10^−1^	***3.44 × 10^−1^***
	Ave	2.33 × 10^0^	1.45 × 10^0^	5.89 × 10^1^	1.55 × 10^4^	4.49 × 10^0^	7.64 × 10^−1^	4.74 × 10^1^	9.06 × 10^1^	1.22 × 10^1^	1.35 × 10^5^	2.98 × 10^0^	1.24 × 10^0^	***6.17 × 10^−1^***
	Std	1.33 × 10^0^	5.40 × 10^−1^	7.12 × 10^1^	1.65 × 10^4^	3.33 × 10^0^	***1.51 × 10^−1^***	1.37 × 10^2^	2.77 × 10^2^	7.99 × 10^0^	1.29 × 10^5^	5.83 × 10^−1^	4.35 × 10^−1^	1.89 × 10^−1^
F16	Min	1.35 × 10^0^	1.52 × 10^−1^	2.63 × 10^0^	3.89 × 10^0^	2.20 × 10^0^	1.80 × 10^0^	2.26 × 10^0^	2.57 × 10^0^	3.44 × 10^0^	2.38 × 10^0^	1.77 × 10^0^	***1.24 × 10^0^***	1.24 × 10^0^
	Ave	2.41 × 10^0^	1.66 × 10^0^	3.45 × 10^0^	4.31 × 10^0^	2.93 × 10^0^	2.47 × 10^0^	2.88 × 10^0^	3.38 × 10^0^	3.65 × 10^0^	3.39 × 10^0^	2.47 × 10^0^	***2.20 × 10^0^***	2.32 × 10^0^
	Std	4.00 × 10^−1^	8.31 × 10^−1^	3.41 × 10^−1^	1.81 × 10^−1^	3.18 × 10^−1^	3.01 × 10^−1^	2.85 × 10^−1^	4.04 × 10^−1^	***1.02 × 10^−1^***	4.52 × 10^−1^	3.52 × 10^−1^	5.01 × 10^−1^	3.92 × 10^−1^

**Table 4:** The results of ICOA and competitor algorithms for CEC-2014 hybrid functions (D=10).

Func.	Index	Algorithms												
		PSO	DE	WOA	AOA	SHO	AVOA	BBOA	EVO	GJO	HCO	RUN	COA	ICOA
F17	Min	7.58 × 10^0^	2.08 × 10^1^	6.77 × 10^2^	5.83 × 10^5^	4.62 × 10^3^	8.60 × 10^2^	6.22 × 10^2^	6.34 × 10^3^	8.93 × 10^3^	6.20 × 10^5^	1.60 × 10^3^	2.29 × 10^2^	***7.40 × 10^0^***
	Ave	1.15 × 10^3^	2.73 × 10^2^	1.95 × 10^3^	7.14 × 10^6^	9.24 × 10^3^	2.62 × 10^3^	2.74 × 10^3^	1.39 × 10^5^	4.33 × 10^4^	1.05 × 10^7^	8.92 × 10^3^	4.89 × 10^3^	***2.51 × 10^2^***
	Std	1.68 × 10^3^	***2.07 × 10^2^***	1.21 × 10^3^	1.03 × 10^7^	4.33 × 10^3^	1.42 × 10^3^	2.56 × 10^3^	4.26 × 10^5^	3.09 × 10^4^	8.43 × 10^6^	4.51 × 10^3^	5.48 × 10^3^	2.20 × 10^2^
F18	Min	4.95 × 10^0^	2.95 × 10^0^	1.32 × 10^2^	1.33 × 10^6^	1.03 × 10^4^	1.35 × 10^1^	1.13 × 10^3^	6.16 × 10^3^	1.78 × 10^4^	3.90 × 10^6^	1.32 × 10^3^	8.46 × 10^0^	***2.91 × 10^0^***
	Ave	4.62 × 10^1^	2.39 × 10^1^	5.22 × 10^3^	1.48 × 10^8^	1.62 × 10^4^	1.06 × 10^3^	9.66 × 10^3^	1.99 × 10^6^	1.94 × 10^4^	1.20 × 10^8^	4.11 × 10^3^	6.23 × 10^1^	***1.99 × 10^1^***
	Std	1.45 × 10^2^	***1.86 × 10^1^***	4.31 × 10^3^	1.61 × 10^8^	2.97 × 10^3^	1.18 × 10^3^	4.91 × 10^3^	6.97 × 10^6^	4.36 × 10^2^	1.08 × 10^8^	2.38 × 10^3^	7.53 × 10^1^	2.19 × 10^1^
F19	Min	1.66 × 10^0^	3.18 × 10^0^	6.80 × 10^0^	1.22 × 10^4^	4.32 × 10^0^	2.05 × 10^0^	2.02 × 10^0^	5.92 × 10^0^	7.99 × 10^0^	1.44 × 10^5^	4.23 × 10^0^	1.58 × 10^0^	***1.16 × 10^0^***
	Ave	1.08 × 10^1^	5.17 × 10^0^	3.07 × 10^1^	7.63 × 10^6^	8.31 × 10^0^	5.72 × 10^0^	4.51 × 10^0^	3.80 × 10^4^	2.98 × 10^1^	3.29 × 10^6^	9.35 × 10^0^	7.12 × 10^0^	***3.55 × 10^0^***
	Std	7.61 × 10^0^	***1.10 × 10^0^***	3.93 × 10^1^	1.41 × 10^7^	1.66 × 10^0^	2.39 × 10^0^	2.42 × 10^0^	1.77 × 10^5^	9.25 × 10^1^	3.33 × 10^6^	2.61 × 10^0^	4.86 × 10^0^	2.20 × 10^0^
F20	Min	4.20 × 10^0^	1.96 × 10^0^	2.18 × 10^3^	1.26 × 10^9^	5.94 × 10^3^	6.04 × 10^1^	8.07 × 10^2^	1.81 × 10^3^	1.24 × 10^4^	2.30 × 10^9^	7.94 × 10^3^	1.30 × 10^2^	***1.13 × 10^0^***
	Ave	1.82 × 10^3^	2.63 × 10^1^	1.18 × 10^4^	2.34 × 10^13^	1.62 × 10^4^	5.80 × 10^3^	2.30 × 10^4^	4.38 × 10^1^⁰	4.50 × 10^4^	4.27 × 10^1^^2^	2.35 × 10^4^	6.95 × 10^3^	***2.61 × 10^1^***
	Std	3.21 × 10^3^	***2.03 × 10^1^***	1.10 × 10^4^	6.72 × 10^13^	6.85 × 10^3^	5.21 × 10^3^	2.08 × 10^4^	2.27 × 10^1^^1^	1.30 × 10^5^	9.67 × 10^1^^2^	1.18 × 10^4^	9.02 × 10^3^	3.48 × 10^1^
F21	Min	1.36 × 10^0^	4.98 × 10^−1^	3.68 × 10^0^	7.10 × 10^5^	2.87 × 10^3^	4.12 × 10^0^	2.61 × 10^2^	9.54 × 10^2^	1.52 × 10^4^	1.72 × 10^6^	9.62 × 10^2^	4.01 × 10^0^	***4.97 × 10^−1^***
	Ave	4.32 × 10^1^	4.09 × 10^0^	5.21 × 10^2^	5.18 × 10^7^	5.86 × 10^3^	2.18 × 10^2^	2.44 × 10^3^	3.11 × 10^5^	1.43 × 10^5^	3.96 × 10^7^	2.50 × 10^3^	5.56 × 10^1^	***3.91 × 10^0^***
	Std	4.41 × 10^1^	***3.79 × 10^0^***	8.02 × 10^2^	5.72 × 10^7^	1.18 × 10^3^	1.94 × 10^2^	2.54 × 10^3^	5.71 × 10^5^	1.39 × 10^5^	3.23 × 10^7^	1.26 × 10^3^	5.47 × 10^1^	8.48 × 10^0^
F22	Min	2.06 × 10^1^	8.56 × 10^−2^	2.07 × 10^1^	1.19 × 10^3^	2.13 × 10^1^	2.03 × 10^1^	2.10 × 10^1^	6.34 × 10^1^	7.39 × 10^1^	1.01 × 10^3^	2.83 × 10^1^	2.03 × 10^1^	***8.54 × 10^−2^***
	Ave	1.83 × 10^2^	5.29 × 10^2^	1.20 × 10^2^	3.61 × 10^1^^1^	2.06 × 10^2^	7.88 × 10^1^	1.19 × 10^2^	3.35 × 10^7^	3.20 × 10^2^	8.66 × 10^1^⁰	***4.31 × 10^1^***	1.35 × 10^2^	9.83 × 10^1^
	Std	9.83 × 10^1^	8.17 × 10^2^	1.14 × 10^2^	1.34 × 10^1^^2^	2.56 × 10^2^	9.76 × 10^1^	1.34 × 10^2^	1.79 × 10^8^	2.95 × 10^2^	2.29 × 10^1^^1^	***4.05 × 10^1^***	1.69 × 10^2^	1.07 × 10^2^

**Table 5:** The results of ICOA and competitor algorithms for CEC-2014 composition functions (D=10).

Func.	İndex	Algorithms												
		PSO	DE	WOA	AOA	SHO	AVOA	BBOA	EVO	GJO	HCO	RUN	COA	ICOA
F23	Min	2.37 × 10^2^	2.37 × 10^2^	***2.00 × 10^2^***	2.00 × 10^2^	***2.00 × 10^2^***	***2.00 × 10^2^***	***2.00 × 10^2^***	2.43 × 10^2^	***2.00 × 10^2^***	2.52 × 10^2^	2.00 × 10^2^	***2.00 × 10^2^***	***2.00 × 10^2^***
	Ave	2.42 × 10^2^	2.37 × 10^2^	2.38 × 10^2^	2.05 × 10^2^	***2.00 × 10^2^***	***2.00 × 10^2^***	***2.00 × 10^2^***	2.52 × 10^2^	***2.00 × 10^2^***	2.73 × 10^2^	2.04 × 10^2^	***2.00 × 10^2^***	***2.00 × 10^2^***
	Std	1.65 × 10^0^	1.65 × 10^0^	1.29 × 10^1^	1.12 × 10^0^	***0.00 × 10^0^***	***0.00 × 10^0^***	***0.00 × 10^0^***	5.66 × 10^0^	***0.00 × 10^0^***	1.90 × 10^1^	1.13 × 10^1^	***0.00 × 10^0^***	***0.00 × 10^0^***
F24	Min	1.16 × 10^2^	1.11 × 10^2^	1.23 × 10^2^	1.88 × 10^2^	1.16 × 10^2^	1.15 × 10^2^	1.29 × 10^2^	1.21 × 10^2^	1.50 × 10^2^	1.23 × 10^2^	1.15 × 10^2^	1.16 × 10^2^	***1.10 × 10^2^***
	Ave	1.35 × 10^2^	1.25 × 10^2^	1.68 × 10^2^	2.00 × 10^2^	1.45 × 10^2^	1.32 × 10^2^	1.63 × 10^2^	1.57 × 10^2^	1.56 × 10^2^	1.63 × 10^2^	1.25 × 10^2^	1.98 × 10^2^	***1.21 × 10^2^***
	Std	1.18 × 10^1^	5.39 × 10^0^	3.01 × 10^1^	***2.73 × 10^0^***	2.67 × 10^1^	1.60 × 10^1^	2.53 × 10^1^	2.33 × 10^1^	3.99 × 10^0^	2.47 × 10^1^	6.09 × 10^0^	1.54 × 10^1^	6.75 × 10^0^
F25	Min	1.55 × 10^2^	2.00 × 10^2^	1.66 × 10^2^	2.00 × 10^2^	1.60 × 10^2^	2.00 × 10^2^	1.82 × 10^2^	2.02 × 10^2^	2.00 × 10^2^	2.01 × 10^2^	***1.55 × 10^2^***	2.00 × 10^2^	2.00 × 10^2^
	Ave	***1.95 × 10^2^***	2.00 × 10^2^	1.99 × 10^2^	2.00 × 10^2^	1.97 × 10^2^	2.00 × 10^2^	1.99 × 10^2^	2.04 × 10^2^	2.00 × 10^2^	2.06 × 10^2^	1.95 × 10^2^	2.00 × 10^2^	2.00 × 10^2^
	Std	1.42 × 10^1^	1.45 × 10^−13^	7.34 × 10^0^	3.58 × 10^−2^	9.47 × 10^0^	***0.00 × 10^0^***	4.35 × 10^0^	1.65 × 10^0^	***0.00 × 10^0^***	1.71 × 10^0^	1.49 × 10^1^	***0.00 × 10^0^***	***0.00 × 10^0^***
F26	Min	1.00 × 10^2^	***1.00 × 10^2^***	1.00 × 10^2^	1.03 × 10^2^	1.00 × 10^2^	1.00 × 10^2^	1.00 × 10^2^	1.00 × 10^2^	1.00 × 10^2^	1.01 × 10^2^	1.00 × 10^2^	1.00 × 10^2^	1.00 × 10^2^
	Ave	1.00 × 10^2^	1.00 × 10^2^	1.01 × 10^2^	1.06 × 10^2^	1.04 × 10^2^	***1.00 × 10^2^***	1.01 × 10^2^	1.01 × 10^2^	1.01 × 10^2^	1.03 × 10^2^	1.00 × 10^2^	1.00 × 10^2^	1.00 × 10^2^
	Std	2.14 × 10^−1^	5.78 × 10^−2^	1.99 × 10^−1^	4.16 × 10^0^	1.81 × 10^1^	***2.44 × 10^−2^***	7.24 × 10^−1^	5.85 × 10^−1^	3.59 × 10^−1^	1.32 × 10^0^	5.89 × 10^−2^	1.53 × 10^−1^	2.57 × 10^−2^
F27	Min	5.00 × 10^0^	5.00 × 10^0^	8.29 × 10^0^	2.04 × 10^2^	1.24 × 10^1^	5.00 × 10^0^	1.15 × 10^1^	1.22 × 10^2^	2.10 × 10^2^	1.58 × 10^2^	7.29 × 10^0^	5.00 × 10^0^	***5.00 × 10^0^***
	Ave	3.17 × 10^2^	3.37 × 10^2^	3.90 × 10^2^	2.04 × 10^2^	3.30 × 10^2^	1.48 × 10^2^	3.58 × 10^2^	4.14 × 10^2^	3.96 × 10^2^	4.25 × 10^2^	3.58 × 10^2^	1.83 × 10^2^	***6.43 × 10^1^***
	Std	1.59 × 10^2^	1.36 × 10^2^	7.22 × 10^1^	***2.89 × 10^−14^***	1.53 × 10^2^	8.77 × 10^1^	1.54 × 10^2^	8.54 × 10^1^	1.06 × 10^2^	7.71 × 10^1^	1.21 × 10^2^	5.35 × 10^1^	9.04 × 10^1^
F28	Min	3.78 × 10^2^	3.69 × 10^2^	***2.00 × 10^2^***	2.24 × 10^2^	4.02 × 10^2^	***2.00 × 10^2^***	3.07 × 10^2^	3.92 × 10^2^	4.17 × 10^2^	7.47 × 10^2^	3.73 × 10^2^	***2.00 × 10^2^***	***2.00 × 10^2^***
	Ave	5.09 × 10^2^	3.83 × 10^2^	7.27 × 10^2^	2.24 × 10^2^	5.16 × 10^2^	***2.00 × 10^2^***	3.36 × 10^2^	6.00 × 10^2^	5.03 × 10^2^	1.10 × 10^3^	4.67 × 10^2^	***2.00 × 10^2^***	***2.00 × 10^2^***
	Std	9.32 × 10^1^	2.68 × 10^1^	1.79 × 10^2^	5.78 × 10^−14^	7.79 × 10^1^	***0.00 × 10^0^***	3.06 × 10^1^	1.71 × 10^2^	7.55 × 10^1^	2.18 × 10^2^	8.15 × 10^1^	***0.00 × 10^0^***	***0.00 × 10^0^***
F29	Min	4.36 × 10^6^	4.36 × 10^6^	4.36 × 10^6^	3.23 × 10^6^	5.51 × 10^6^	***2.02 × 10^2^***	2.07 × 10^2^	5.54 × 10^6^	5.61 × 10^6^	7.79 × 10^7^	4.58 × 10^6^	***2.02 × 10^2^***	***2.02 × 10^2^***
	Ave	4.92 × 10^6^	4.60 × 10^6^	1.21 × 10^7^	1.16 × 10^7^	8.80 × 10^6^	***2.02 × 10^2^***	2.83 × 10^2^	1.89 × 10^7^	6.88 × 10^6^	1.83 × 10^8^	5.83 × 10^6^	***2.02 × 10^2^***	***2.02 × 10^2^***
	Std	4.60 × 10^5^	3.93 × 10^5^	7.93 × 10^6^	1.92 × 10^6^	3.51 × 10^6^	***0.00 × 10^0^***	8.13 × 10^1^	9.27 × 10^6^	1.16 × 10^6^	7.49 × 10^7^	8.20 × 10^5^	***0.00 × 10^0^***	***0.00 × 10^0^***
F30	Min	2.29 × 10^3^	1.71 × 10^3^	3.07 × 10^3^	1.20 × 10^6^	***2.03 × 10^2^***	***2.03 × 10^2^***	2.23 × 10^2^	6.28 × 10^5^	***2.03 × 10^2^***	4.09 × 10^7^	4.67 × 10^3^	***2.03 × 10^2^***	***2.03 × 10^2^***
	Ave	2.01 × 10^4^	1.31 × 10^4^	6.04 × 10^6^	1.71 × 10^7^	7.43 × 10^5^	1.15 × 10^4^	9.28 × 10^2^	7.88 × 10^7^	7.41 × 10^5^	2.93 × 10^9^	4.79 × 10^4^	***2.03 × 10^2^***	***2.03 × 10^2^***
	Std	2.93 × 10^4^	2.00 × 10^4^	1.76 × 10^7^	6.27 × 10^6^	1.77 × 10^6^	2.72 × 10^4^	1.09 × 10^3^	9.21 × 10^7^	7.05 × 10^5^	6.41 × 10^9^	5.41 × 10^4^	***2.89 × 10^−14^***	***2.89 × 10^−14^***
F. ave-rank		5.07	3.93	8.57	11.87	7.68	3.50	7.48	9.43	9.37	11.83	5.67	4.48	***2.12***

**Table 6:** The results of ICOA and competitor algorithms for CEC-2014 unimodal functions (D=30).

Func.	Index				Algorithms									
		PSO	DE	WOA	AOA	SHO	AVOA	BBOA	EVO	GJO	HCO	RUN	COA	ICOA
F1	Min	1.63 × 10^5^	1.39 × 10^5^	4.20 × 10^5^	1.09 × 10^9^	8.16 × 10^7^	***1.23 × 10^5^***	2.15 × 10^8^	1.23 × 10^8^	2.66 × 10^8^	1.50 × 10^9^	3.09 × 10^7^	2.30 × 10^5^	1.56 × 10^5^
	Ave	1.29 × 10^6^	6.62 × 10^5^	3.08 × 10^6^	1.77 × 10^9^	2.90 × 10^8^	7.25 × 10^5^	6.27 × 10^8^	4.45 × 10^8^	4.26 × 10^8^	3.31 × 10^9^	6.17 × 10^7^	9.73 × 10^5^	***5.26 × 10^5^***
	Std	1.18 × 10^6^	4.54 × 10^5^	2.76 × 10^6^	2.36 × 10^8^	1.13 × 10^8^	3.76 × 10^5^	1.95 × 10^8^	1.74 × 10^8^	8.46 × 10^7^	9.37 × 10^8^	2.25 × 10^7^	6.49 × 10^5^	***3.23 × 10^5^***
F2	Min	1.27 × 10^0^	1.11 × 10^0^	3.08 × 10^0^	6.48 × 10^10^	1.09 × 10^10^	8.73 × 10^1^	4.24 × 10^10^	1.29 × 10^10^	2.25 × 10^10^	1.04 × 10^1^^1^	1.66 × 10^9^	1.13 × 10^2^	***9.70 × 10^−1^***
	Ave	2.73 × 10^5^	1.68 × 10^4^	1.13 × 10^4^	7.96 × 10^10^	3.16 × 10^10^	1.62 × 10^4^	5.77 × 10^10^	2.17 × 10^10^	3.05 × 10^10^	1.32 × 10^1^^1^	2.79 × 10^9^	1.62 × 10^4^	***9.55 × 10^3^***
	Std	7.92 × 10^5^	1.44 × 10^4^	1.41 × 10^4^	5.74 × 10^9^	8.77 × 10^9^	***1.18 × 10^4^***	8.36 × 10^9^	5.54 × 10^9^	4.01 × 10^9^	1.40 × 10^10^	6.33 × 10^8^	1.25 × 10^4^	1.31 × 10^4^
F3	Min	4.51 × 10^−2^	2.54 × 10^0^	6.99 × 10^0^	1.02 × 10^5^	3.13 × 10^4^	1.64 × 10^1^	3.66 × 10^4^	3.90 × 10^4^	6.24 × 10^4^	2.82 × 10^5^	7.25 × 10^3^	4.67 × 10^1^	***3.63 × 10^−2^***
	Ave	5.15 × 10^0^	9.29 × 10^0^	1.64 × 10^3^	5.05 × 10^6^	4.88 × 10^4^	7.16 × 10^2^	5.90 × 10^4^	6.99 × 10^4^	7.10 × 10^4^	2.69 × 10^6^	1.18 × 10^4^	3.20 × 10^2^	***1.81 × 10^0^***
	Std	1.13 × 10^1^	6.01 × 10^0^	2.68 × 10^3^	7.66 × 10^6^	8.61 × 10^3^	5.26 × 10^2^	8.99 × 10^3^	2.05 × 10^4^	5.18 × 10^3^	3.39 × 10^6^	1.94 × 10^3^	3.09 × 10^2^	***4.25 × 10^0^***

**Table 7:** The results of ICOA and competitor algorithms for CEC-2014 multimodal functions (D=30).

Func.	Index				Algorithms									
		PSO	DE	WOA	AOA	SHO	AVOA	BBOA	EVO	GJO	HCO	RUN	COA	ICOA
F4	Min	6.53 × 10^1^	5.47 × 10^0^	8.06 × 10^1^	8.55 × 10^3^	6.56 × 10^2^	1.67 × 10^−1^	4.04 × 10^3^	7.41 × 10^2^	9.21 × 10^2^	5.89 × 10^3^	2.02 × 10^2^	8.27 × 10^0^	***1.55 × 10^−3^***
	Ave	1.07 × 10^2^	4.23 × 10^6^	1.39 × 10^2^	1.49 × 10^4^	2.36 × 10^3^	7.62 × 10^1^	8.21 × 10^3^	2.56 × 10^3^	1.28 × 10^3^	1.10 × 10^4^	3.04 × 10^2^	8.60 × 10^1^	***5.88 × 10^1^***
	Std	2.69 × 10^1^	1.61 × 10^7^	2.73 × 10^1^	2.84 × 10^3^	1.20 × 10^3^	***2.41 × 10^1^***	2.17 × 10^3^	1.12 × 10^3^	2.36 × 10^2^	2.54 × 10^3^	6.57 × 10^1^	2.91 × 10^1^	2.91 × 10^1^
F5	Min	2.01 × 10^1^	2.03 × 10^1^	2.00 × 10^1^	2.11 × 10^1^	2.01 × 10^1^	***2.00 × 10^1^***	2.04 × 10^1^	2.00 × 10^1^	2.08 × 10^1^	2.07 × 10^1^	2.08 × 10^1^	2.00 × 10^1^	2.00 × 10^1^
	Ave	2.08 × 10^1^	2.07 × 10^1^	2.02 × 10^1^	2.13 × 10^1^	2.04 × 10^1^	2.01 × 10^1^	2.06 × 10^1^	2.04 × 10^1^	2.09 × 10^1^	2.10 × 10^1^	2.09 × 10^1^	2.01 × 10^1^	***2.00 × 10^1^***
	Std	2.11 × 10^−1^	1.93 × 10^−1^	2.70 × 10^−1^	7.94 × 10^−2^	1.32 × 10^−1^	1.24 × 10^−1^	1.01 × 10^−1^	5.77 × 10^−1^	6.65 × 10^−2^	9.73 × 10^−2^	6.75 × 10^−2^	1.03 × 10^−1^	***2.65 × 10^−2^***
F6	Min	1.82 × 10^1^	1.16 × 10^1^	3.45 × 10^1^	4.16 × 10^1^	2.48 × 10^1^	1.26 × 10^1^	2.98 × 10^1^	1.94 × 10^1^	3.02 × 10^1^	3.41 × 10^1^	2.11 × 10^1^	1.64 × 10^1^	***1.12 × 10^1^***
	Ave	2.59 × 10^1^	2.23 × 10^1^	4.03 × 10^1^	4.51 × 10^1^	3.07 × 10^1^	2.01 × 10^1^	3.40 × 10^1^	2.66 × 10^1^	3.15 × 10^1^	3.71 × 10^1^	2.51 × 10^1^	2.23 × 10^1^	***1.62 × 10^1^***
	Std	3.28 × 10^0^	9.64 × 10^0^	2.08 × 10^0^	1.61 × 10^0^	2.19 × 10^0^	3.81 × 10^0^	1.99 × 10^0^	3.74 × 10^0^	***7.77 × 10^−1^***	2.12 × 10^0^	2.36 × 10^0^	2.97 × 10^0^	2.75 × 10^0^
F7	Min	3.35 × 10^−3^	6.01 × 10^−8^	1.62 × 10^−8^	6.09 × 10^2^	1.45 × 10^2^	7.05 × 10^−13^	3.33 × 10^2^	6.53 × 10^1^	1.70 × 10^2^	2.49 × 10^2^	1.76 × 10^1^	1.56 × 10^−3^	***7.77 × 10^−16^***
	Ave	5.66 × 10^−1^	1.35 × 10^−2^	2.90 × 10^−1^	7.56 × 10^2^	2.83 × 10^2^	2.25 × 10^−2^	5.64 × 10^2^	2.00 × 10^2^	2.32 × 10^2^	3.44 × 10^2^	2.59 × 10^1^	7.77 × 10^−2^	***1.25 × 10^−2^***
	Std	6.48 × 10^−1^	***1.58 × 10^−2^***	8.47 × 10^−1^	6.26 × 10^1^	8.44 × 10^1^	3.11 × 10^−2^	9.04 × 10^1^	5.97 × 10^1^	3.14 × 10^1^	5.55 × 10^1^	4.44 × 10^0^	8.90 × 10^−2^	1.84 × 10^−2^
F8	Min	5.37 × 10^1^	1.03 × 10^1^	8.76 × 10^1^	4.16 × 10^2^	1.41 × 10^2^	1.09 × 10^1^	1.91 × 10^2^	5.36 × 10^1^	2.30 × 10^2^	1.88 × 10^2^	9.15 × 10^1^	5.09 × 10^1^	***9.95 × 10^−1^***
	Ave	1.09 × 10^2^	2.50 × 10^1^	2.02 × 10^2^	4.61 × 10^2^	2.03 × 10^2^	3.22 × 10^1^	2.41 × 10^2^	9.00 × 10^1^	2.69 × 10^2^	2.44 × 10^2^	1.61 × 10^2^	1.13 × 10^2^	***2.47 × 10^1^***
	Std	2.75 × 10^1^	***1.12 × 10^1^***	5.17 × 10^1^	2.08 × 10^1^	2.73 × 10^1^	1.24 × 10^1^	1.97 × 10^1^	2.14 × 10^1^	1.49 × 10^1^	2.71 × 10^1^	2.47 × 10^1^	3.56 × 10^1^	1.48 × 10^1^
F9	Min	8.66 × 10^1^	4.85 × 10^1^	1.44 × 10^2^	3.74 × 10^2^	1.84 × 10^2^	9.75 × 10^1^	2.01 × 10^2^	1.10 × 10^2^	2.54 × 10^2^	1.94 × 10^2^	1.72 × 10^2^	1.02 × 10^2^	***4.58 × 10^1^***
	Ave	1.63 × 10^2^	1.55 × 10^2^	2.75 × 10^2^	4.11 × 10^2^	2.29 × 10^2^	1.61 × 10^2^	2.59 × 10^2^	1.57 × 10^2^	2.81 × 10^2^	2.64 × 10^2^	2.11 × 10^2^	1.79 × 10^2^	***1.21 × 10^2^***
	Std	4.48 × 10^1^	1.70 × 10^2^	7.15 × 10^1^	1.78 × 10^1^	2.48 × 10^1^	3.31 × 10^1^	3.02 × 10^1^	3.16 × 10^1^	***1.36 × 10^1^***	2.45 × 10^1^	2.02 × 10^1^	2.00 × 10^1^	2.75 × 10^1^
F10	Min	7.14 × 10^2^	1.11 × 10^2^	2.93 × 10^3^	7.90 × 10^3^	3.47 × 10^3^	3.16 × 10^1^	4.22 × 10^3^	1.04 × 10^3^	5.53 × 10^3^	6.52 × 10^3^	2.22 × 10^3^	1.25 × 10^2^	***1.14 × 10^1^***
	Ave	1.63 × 10^3^	5.68 × 10^2^	4.35 × 10^3^	8.98 × 10^3^	4.34 × 10^3^	5.21 × 10^2^	5.42 × 10^3^	2.01 × 10^3^	5.90 × 10^3^	7.64 × 10^3^	3.96 × 10^3^	1.15 × 10^3^	***3.01 × 10^2^***
	Std	5.22 × 10^2^	2.79 × 10^2^	7.15 × 10^2^	4.86 × 10^2^	5.08 × 10^2^	3.03 × 10^2^	5.53 × 10^2^	4.38 × 10^2^	1.96 × 10^2^	4.46 × 10^2^	8.39 × 10^2^	8.11 × 10^2^	***1.89 × 10^2^***
F11	Min	2.06 × 10^3^	2.11 × 10^3^	3.09 × 10^3^	7.85 × 10^3^	2.95 × 10^3^	2.55 × 10^3^	4.74 × 10^3^	2.82 × 10^3^	5.56 × 10^3^	7.25 × 10^3^	4.59 × 10^3^	2.90 × 10^3^	***1.87 × 10^3^***
	Ave	3.14 × 10^3^	4.58 × 10^3^	5.64 × 10^3^	9.11 × 10^3^	4.88 × 10^3^	3.55 × 10^3^	5.87 × 10^3^	4.45 × 10^3^	6.19 × 10^3^	7.91 × 10^3^	6.06 × 10^3^	4.44 × 10^3^	***3.01 × 10^3^***
	Std	6.13 × 10^2^	1.80 × 10^3^	1.23 × 10^3^	4.41 × 10^2^	7.42 × 10^2^	6.08 × 10^2^	5.24 × 10^2^	9.78 × 10^2^	4.39 × 10^2^	***3.63 × 10^2^***	6.12 × 10^2^	5.71 × 10^2^	5.07 × 10^2^
F12	Min	1.54 × 10^−1^	1.77 × 10^−1^	1.48 × 10^0^	3.20 × 10^0^	6.18 × 10^−1^	***3.07 × 10^−2^***	2.01 × 10^−1^	3.70 × 10^−1^	1.16 × 10^0^	1.38 × 10^0^	1.13 × 10^0^	3.31 × 10^−1^	1.34 × 10^−1^
	Ave	5.12 × 10^−1^	1.43 × 10^0^	2.37 × 10^0^	4.82 × 10^0^	1.02 × 10^0^	***1.02 × 10^−1^***	1.26 × 10^0^	1.61 × 10^0^	1.71 × 10^0^	3.01 × 10^0^	2.26 × 10^0^	1.03 × 10^0^	5.21 × 10^−1^
	Std	2.44 × 10^−1^	8.31 × 10^−1^	4.71 × 10^−1^	8.11 × 10^−1^	2.56 × 10^−1^	***7.07 × 10^−2^***	4.55 × 10^−1^	1.35 × 10^0^	2.00 × 10^−1^	6.91 × 10^−1^	3.45 × 10^−1^	3.73 × 10^−1^	2.26 × 10^−1^
F13	Min	4.55 × 10^−1^	3.53 × 10^−1^	4.06 × 10^−1^	6.88 × 10^0^	3.19 × 10^0^	***2.38 × 10^−1^***	5.16 × 10^0^	6.44 × 10^−1^	3.13 × 10^0^	4.06 × 10^0^	5.39 × 10^−1^	3.28 × 10^−1^	3.02 × 10^−1^
	Ave	6.99 × 10^−1^	5.33 × 10^−1^	6.43 × 10^−1^	7.69 × 10^0^	3.97 × 10^0^	***3.66 × 10^−1^***	6.19 × 10^0^	3.20 × 10^0^	3.64 × 10^0^	5.03 × 10^0^	7.53 × 10^−1^	5.55 × 10^−1^	5.49 × 10^−1^
	Std	1.54 × 10^−1^	8.03 × 10^−2^	1.09 × 10^−1^	4.20 × 10^−1^	5.41 × 10^−1^	***7.07 × 10^−2^***	6.00 × 10^−1^	1.03 × 10^0^	2.61 × 10^−1^	4.99 × 10^−1^	1.07 × 10^−1^	1.62 × 10^−1^	1.05 × 10^−1^
F14	Min	1.72 × 10^−1^	2.07 × 10^−1^	2.15 × 10^−1^	1.54 × 10^2^	4.23 × 10^1^	1.73 × 10^−1^	1.29 × 10^2^	2.87 × 10^1^	5.78 × 10^1^	4.39 × 10^1^	4.54 × 10^−1^	2.42 × 10^−1^	***1.46 × 10^−1^***
	Ave	5.17 × 10^−1^	3.71 × 10^−1^	4.26 × 10^−1^	2.21 × 10^2^	7.68 × 10^1^	4.86 × 10^−1^	1.68 × 10^2^	5.05 × 10^1^	6.88 × 10^1^	7.98 × 10^1^	3.49 × 10^0^	5.01 × 10^−1^	***3.37 × 10^−1^***
	Std	3.05 × 10^−1^	1.39 × 10^−1^	2.87 × 10^−1^	2.79 × 10^1^	1.77 × 10^1^	1.72 × 10^−1^	2.04 × 10^1^	1.51 × 10^1^	7.25 × 10^0^	1.71 × 10^1^	2.52 × 10^0^	2.98 × 10^−1^	***1.36 × 10^−1^***
F15	Min	1.24 × 10^1^	7.40 × 10^0^	6.71 × 10^2^	2.63 × 10^5^	6.86 × 10^2^	***2.38 × 10^0^***	1.57 × 10^4^	2.28 × 10^3^	3.68 × 10^3^	2.30 × 10^6^	2.75 × 10^1^	7.11 × 10^0^	5.81 × 10^0^
	Ave	3.43 × 10^1^	1.56 × 10^1^	1.94 × 10^3^	5.29 × 10^5^	1.42 × 10^4^	***4.70 × 10^0^***	8.44 × 10^4^	1.84 × 10^4^	1.27 × 10^4^	1.85 × 10^7^	4.03 × 10^1^	1.65 × 10^1^	1.08 × 10^1^
	Std	1.61 × 10^1^	2.15 × 10^0^	1.00 × 10^3^	2.01 × 10^5^	1.53 × 10^4^	***1.31 × 10^0^***	5.25 × 10^4^	1.57 × 10^4^	6.03 × 10^3^	1.00 × 10^7^	9.14 × 10^0^	7.45 × 10^0^	3.48 × 10^0^
F16	Min	1.02 × 10^1^	9.37 × 10^0^	1.14 × 10^1^	1.34 × 10^1^	1.17 × 10^1^	9.96 × 10^0^	1.18 × 10^1^	1.22 × 10^1^	1.23 × 10^1^	1.27 × 10^1^	1.18 × 10^1^	9.86 × 10^0^	***9.31 × 10^0^***
	Ave	1.12 × 10^1^	1.19 × 10^1^	1.27 × 10^1^	1.41 × 10^1^	1.23 × 10^1^	1.15 × 10^1^	1.23 × 10^1^	1.31 × 10^1^	1.28 × 10^1^	1.33 × 10^1^	1.21 × 10^1^	1.09 × 10^1^	***1.07 × 10^1^***
	Std	6.08 × 10^−1^	1.05 × 10^0^	5.41 × 10^−1^	2.61 × 10^−1^	3.26 × 10^−1^	4.45 × 10^−1^	3.02 × 10^−1^	5.42 × 10^−1^	2.32 × 10^−1^	3.69 × 10^−1^	***2.24 × 10^−1^***	5.00 × 10^−1^	6.65 × 10^−1^

**Table 8:** The results of ICOA and competitor algorithms for CEC-2014 hybrid functions (D=30).

Func.	Index	Algorithms												
		PSO	DE	WOA	AOA	SHO	AVOA	BBOA	EVO	GJO	HCO	RUN	COA	ICOA
F17	Min	1.83 × 10^4^	1.59 × 10^4^	3.11 × 10^4^	1.45 × 10^8^	1.10 × 10^6^	2.77 × 10^4^	2.65 × 10^6^	8.80 × 10^6^	3.49 × 10^6^	8.15 × 10^7^	3.36 × 10^5^	3.34 × 10^4^	***1.45 × 10^4^***
	Ave	1.44 × 10^5^	8.23 × 10^4^	3.49 × 10^5^	3.56 × 10^8^	1.14 × 10^7^	9.28 × 10^4^	1.46 × 10^7^	5.37 × 10^7^	1.32 × 10^7^	4.48 × 10^8^	1.57 × 10^6^	1.26 × 10^5^	***4.05 × 10^4^***
	Std	2.36 × 10^5^	6.24 × 10^4^	3.50 × 10^5^	1.38 × 10^8^	1.34 × 10^7^	3.45 × 10^4^	1.13 × 10^7^	5.54 × 10^7^	8.33 × 10^6^	2.10 × 10^8^	6.22 × 10^5^	1.44 × 10^5^	***1.73 × 10^4^***
F18	Min	6.76 × 10^4^	3.71 × 10^4^	4.29 × 10^4^	4.81 × 10^9^	5.33 × 10^4^	6.81 × 10^4^	2.14 × 10^7^	2.52 × 10^5^	7.79 × 10^6^	4.32 × 10^9^	1.01 × 10^7^	6.64 × 10^4^	***3.65 × 10^4^***
	Ave	1.04 × 10^5^	8.18 × 10^4^	1.06 × 10^5^	9.45 × 10^9^	6.12 × 10^7^	1.08 × 10^5^	9.51 × 10^8^	4.31 × 10^7^	4.33 × 10^7^	1.02 × 10^10^	3.81 × 10^7^	1.12 × 10^5^	***7.81 × 10^4^***
	Std	2.66 × 10^4^	2.65 × 10^4^	3.41 × 10^4^	2.73 × 10^9^	1.50 × 10^8^	***2.47 × 10^4^***	8.51 × 10^8^	9.94 × 10^7^	2.05 × 10^7^	3.66 × 10^9^	1.47 × 10^7^	3.08 × 10^4^	2.63 × 10^4^
F19	Min	1.35 × 10^1^	2.01 × 10^1^	3.86 × 10^1^	1.71 × 10^8^	2.58 × 10^1^	***1.10 × 10^1^***	4.90 × 10^2^	1.62 × 10^3^	3.56 × 10^5^	8.75 × 10^8^	1.89 × 10^3^	1.67 × 10^1^	1.74 × 10^1^
	Ave	3.58 × 10^2^	**2.41 × 10^1^**	1.81 × 10^3^	1.51 × 10^9^	1.53 × 10^5^	5.15 × 10^1^	9.25 × 10^6^	8.71 × 10^5^	2.42 × 10^6^	5.00 × 10^9^	1.28 × 10^5^	1.38 × 10^2^	1.29 × 10^2^
	Std	9.96 × 10^2^	**3.15 × 10^0^**	2.97 × 10^3^	7.43 × 10^8^	6.62 × 10^5^	8.74 × 10^1^	1.91 × 10^7^	2.49 × 10^6^	2.65 × 10^6^	2.56 × 10^9^	1.01 × 10^5^	4.57 × 10^2^	2.75 × 10^2^
F20	Min	2.64 × 10^4^	4.76 × 10^3^	2.52 × 10^4^	6.06 × 10^12^	8.98 × 10^4^	1.88 × 10^4^	1.40 × 10^8^	4.62 × 10^8^	2.60 × 10^9^	2.20 × 10^14^	5.67 × 10^5^	2.26 × 10^4^	***4.56 × 10^3^***
	Ave	8.71 × 10^4^	4.57 × 10^4^	8.79 × 10^4^	1.47 × 10^15^	1.25 × 10^10^	4.93 × 10^4^	6.04 × 10^12^	7.15 × 10^11^	1.14 × 10^10^	1.15 × 10^16^	1.49 × 10^7^	9.23 × 10^4^	***3.50 × 10^4^***
	Std	4.18 × 10^4^	6.64 × 10^4^	2.77 × 10^4^	1.18 × 10^15^	2.78 × 10^10^	2.04 × 10^4^	1.64 × 10^13^	1.81 × 10^12^	9.02 × 10^9^	1.40 × 10^16^	1.81 × 10^7^	5.41 × 10^4^	***2.03 × 10^4^***
F21	Min	3.91 × 10^3^	3.92 × 10^3^	8.42 × 10^3^	1.02 × 10^9^	3.10 × 10^6^	1.25 × 10^4^	2.34 × 10^7^	6.75 × 10^6^	3.54 × 10^7^	1.42 × 10^9^	8.27 × 10^5^	1.31 × 10^4^	***3.81 × 10^3^***
	Ave	2.52 × 10^4^	2.52 × 10^4^	4.33 × 10^4^	3.44 × 10^9^	3.89 × 10^7^	3.50 × 10^4^	1.28 × 10^8^	5.84 × 10^7^	8.81 × 10^7^	3.85 × 10^9^	2.49 × 10^6^	4.58 × 10^4^	***2.06 × 10^4^***
	Std	1.17 × 10^4^	1.45 × 10^4^	2.56 × 10^4^	1.49 × 10^9^	2.85 × 10^7^	1.22 × 10^4^	9.09 × 10^7^	5.13 × 10^7^	3.31 × 10^7^	1.75 × 10^9^	9.68 × 10^5^	1.99 × 10^4^	***1.05 × 10^4^***
F22	Min	2.90 × 10^2^	3.66 × 10^1^	2.49 × 10^3^	5.55 × 10^10^	1.92 × 10^3^	3.02 × 10^1^	2.11 × 10^3^	3.28 × 10^3^	7.80 × 10^5^	1.33 × 10^11^	2.12 × 10^3^	5.89 × 10^1^	***2.83 × 10^1^***
	Ave	1.23 × 10^3^	6.94 × 10^2^	4.91 × 10^3^	3.01 × 10^13^	2.85 × 10^3^	4.99 × 10^2^	7.25 × 10^7^	4.33 × 10^10^	1.57 × 10^7^	1.85 × 10^14^	8.36 × 10^3^	7.14 × 10^2^	***3.63 × 10^2^***
	Std	7.20 × 10^2^	7.41 × 10^2^	1.52 × 10^3^	3.86 × 10^13^	3.85 × 10^2^	***1.92 × 10^2^***	3.56 × 10^8^	1.56 × 10^11^	2.25 × 10^7^	2.20 × 10^14^	8.98 × 10^3^	3.90 × 10^2^	2.21 × 10^2^

**Table 9:** The results of ICOA and competitor algorithms for CEC-2014 composition functions (D=30).

Func.	Index	Algorithms												
		PSO	DE	WOA	AOA	SHO	AVOA	BBOA	EVO	GJO	HCO	RUN	COA	ICOA
F23	Min	2.23 × 10^2^	2.23 × 10^2^	2.23 × 10^2^	2.08 × 10^2^	***2.00 × 10^2^***	***2.00 × 10^2^***	***2.00 × 10^2^***	2.55 × 10^2^	***2.00 × 10^2^***	3.37 × 10^2^	2.25 × 10^2^	***2.00 × 10^2^***	***2.00 × 10^2^***
	Ave	2.23 × 10^2^	2.23 × 10^2^	2.26 × 10^2^	2.12 × 10^2^	2.41 × 10^2^	***2.00 × 10^2^***	2.50 × 10^2^	2.76 × 10^2^	2.30 × 10^2^	4.03 × 10^2^	2.27 × 10^2^	***2.00 × 10^2^***	***2.00 × 10^2^***
	Std	2.35 × 10^−1^	***0.00 × 10^0^***	5.88 × 10^0^	8.41 × 10^−1^	1.11 × 10^1^	***0.00 × 10^0^***	3.00 × 10^1^	1.83 × 10^1^	1.41 × 10^1^	5.69 × 10^1^	2.79 × 10^0^	***0.00 × 10^0^***	***0.00 × 10^0^***
F24	Min	2.23 × 10^2^	***2.01 × 10^2^***	***2.01 × 10^2^***	2.01 × 10^2^	***2.01 × 10^2^***	***2.01 × 10^2^***	***2.01 × 10^2^***	2.47 × 10^2^	***2.01 × 10^2^***	2.43 × 10^2^	2.01 × 10^2^	2.01 × 10^2^	***2.01 × 10^2^***
	Ave	2.42 × 10^2^	***2.01 × 10^2^***	***2.01 × 10^2^***	2.02 × 10^2^	***2.01 × 10^2^***	***2.01 × 10^2^***	***2.01 × 10^2^***	2.62 × 10^2^	***2.01 × 10^2^***	2.51 × 10^2^	2.01 × 10^2^	***2.01 × 10^2^***	***2.01 × 10^2^***
	Std	9.22 × 10^0^	***0.00 × 10^0^***	***0.00 × 10^0^***	2.09 × 10^−1^	***0.00 × 10^0^***	***0.00 × 10^0^***	***0.00 × 10^0^***	8.34 × 10^0^	***0.00 × 10^0^***	6.06 × 10^0^	3.67 × 10^−11^	***0.00 × 10^0^***	***0.00 × 10^0^***
F25	Min	2.06 × 10^2^	2.04 × 10^2^	***2.00 × 10^2^***	2.00 × 10^2^	***2.00 × 10^2^***	***2.00 × 10^2^***	***2.00 × 10^2^***	2.24 × 10^2^	***2.00 × 10^2^***	2.40 × 10^2^	2.00 × 10^2^	***2.00 × 10^2^***	***2.00 × 10^2^***
	Ave	2.17 × 10^2^	2.04 × 10^2^	2.12 × 10^2^	2.01 × 10^2^	***2.00 × 10^2^***	***2.00 × 10^2^***	***2.00 × 10^2^***	2.31 × 10^2^	***2.00 × 10^2^***	2.62 × 10^2^	2.15 × 10^2^	***2.00 × 10^2^***	***2.00 × 10^2^***
	Std	9.14 × 10^0^	3.14 × 10^−1^	2.12 × 10^1^	8.53 × 10^−2^	***0.00 × 10^0^***	***0.00 × 10^0^***	***0.00 × 10^0^***	6.49 × 10^0^	***0.00 × 10^0^***	1.49 × 10^1^	5.01 × 10^0^	***0.00 × 10^0^***	***0.00 × 10^0^***
F26	Min	1.01 × 10^2^	***1.00 × 10^2^***	1.00 × 10^2^	1.10 × 10^2^	1.02 × 10^2^	1.00 × 10^2^	1.05 × 10^2^	1.03 × 10^2^	1.03 × 10^2^	1.14 × 10^2^	1.01 × 10^2^	1.00 × 10^2^	1.00 × 10^2^
	Ave	1.34 × 10^2^	***1.00 × 10^2^***	1.01 × 10^2^	1.93 × 10^2^	1.61 × 10^2^	1.00 × 10^2^	1.12 × 10^2^	1.51 × 10^2^	1.36 × 10^2^	1.50 × 10^2^	1.47 × 10^2^	1.73 × 10^2^	1.01 × 10^2^
	Std	4.76 × 10^1^	***6.83 × 10^−2^***	1.65 × 10^0^	2.30 × 10^1^	4.83 × 10^1^	9.56 × 10^−2^	1.69 × 10^1^	5.07 × 10^1^	4.60 × 10^1^	3.60 × 10^1^	5.07 × 10^1^	4.48 × 10^1^	1.19 × 10^−1^
F27	Min	4.02 × 10^2^	3.71 × 10^2^	6.26 × 10^2^	2.01 × 10^2^	5.26 × 10^2^	***2.00 × 10^2^***	8.74 × 10^2^	9.07 × 10^2^	5.78 × 10^2^	9.32 × 10^2^	4.38 × 10^2^	***2.00 × 10^2^***	***2.00 × 10^2^***
	Ave	9.46 × 10^2^	4.66 × 10^2^	1.45 × 10^3^	2.03 × 10^2^	1.02 × 10^3^	***2.00 × 10^2^***	1.25 × 10^3^	1.07 × 10^3^	1.16 × 10^3^	1.59 × 10^3^	8.87 × 10^2^	2.00 × 10^2^	***2.00 × 10^2^***
	Std	2.05 × 10^2^	6.14 × 10^1^	2.33 × 10^2^	4.73 × 10^−1^	2.30 × 10^2^	***0.00 × 10^0^***	8.52 × 10^1^	9.54 × 10^1^	1.52 × 10^2^	2.45 × 10^2^	1.80 × 10^2^	***0.00 × 10^0^***	***0.00 × 10^0^***
F28	Min	1.16 × 10^3^	7.77 × 10^2^	2.16 × 10^3^	2.06 × 10^2^	***2.00 × 10^2^***	***2.00 × 10^2^***	9.29 × 10^2^	1.16 × 10^3^	***2.00 × 10^2^***	5.29 × 10^3^	1.18 × 10^3^	***2.00 × 10^2^***	***2.00 × 10^2^***
	Ave	1.89 × 10^3^	8.38 × 10^2^	3.07 × 10^3^	2.09 × 10^2^	3.31 × 10^3^	***2.00 × 10^2^***	1.73 × 10^3^	2.26 × 10^3^	4.80 × 10^2^	6.49 × 10^3^	1.47 × 10^3^	***2.00 × 10^2^***	***2.00 × 10^2^***
	Std	5.98 × 10^2^	4.11 × 10^1^	5.85 × 10^2^	7.67 × 10^−1^	8.82 × 10^2^	***0.00 × 10^0^***	5.34 × 10^2^	6.13 × 10^2^	8.11 × 10^2^	7.67 × 10^2^	1.38 × 10^2^	***0.00 × 10^0^***	***0.00 × 10^0^***
F29	Min	3.05 × 10^3^	1.37 × 10^3^	***2.08 × 10^2^***	1.62 × 10^6^	***2.08 × 10^2^***	***2.08 × 10^2^***	***2.08 × 10^2^***	1.24 × 10^8^	***2.08 × 10^2^***	1.38 × 10^9^	2.71 × 10^6^	***2.08 × 10^2^***	***2.08 × 10^2^***
	Ave	8.13 × 10^6^	6.07 × 10^5^	1.41 × 10^7^	1.19 × 10^7^	4.15 × 10^6^	***2.08 × 10^2^***	2.95 × 10^7^	3.05 × 10^8^	1.16 × 10^8^	4.64 × 10^9^	1.23 × 10^7^	***2.08 × 10^2^***	***2.08 × 10^2^***
	Std	2.04 × 10^7^	3.31 × 10^6^	2.47 × 10^7^	2.80 × 10^6^	1.75 × 10^7^	***0.00 × 10^0^***	7.93 × 10^7^	1.31 × 10^8^	6.21 × 10^7^	2.15 × 10^9^	7.15 × 10^6^	***0.00 × 10^0^***	***0.00 × 10^0^***
F30	Min	7.91 × 10^4^	6.79 × 10^3^	5.76 × 10^4^	7.12 × 10^9^	1.22 × 10^8^	2.05 × 10^2^	6.18 × 10^5^	5.59 × 10^8^	1.55 × 10^8^	5.63 × 10^11^	4.02 × 10^6^	***2.05 × 10^2^***	***2.05 × 10^2^***
	Ave	1.92 × 10^7^	1.10 × 10^4^	4.33 × 10^6^	2.44 × 10^11^	4.23 × 10^8^	3.14 × 10^4^	6.32 × 10^8^	1.48 × 10^10^	4.70 × 10^8^	1.64 × 10^14^	3.51 × 10^7^	***9.87 × 10^3^***	1.87 × 10^4^
	Std	2.70 × 10^7^	***2.89 × 10^3^***	7.42 × 10^6^	8.06 × 10^10^	3.06 × 10^8^	4.59 × 10^4^	6.96 × 10^8^	2.70 × 10^10^	3.15 × 10^8^	1.80 × 10^14^	3.46 × 10^7^	2.43 × 10^4^	6.20 × 10^4^
F. ave-rank		5.43	3.95	6.85	11.23	8.00	2.77	9.47	8.93	9.00	12.13	7.40	4.13	1.70

**Table 10:** Comparisons of WSRT for ICOA vs. PSO, DE, and WOA (D=10).

Func.	PSO-ICOA				DE-ICOA				WOA-ICOA			
	*p*-Value	T−	T+	W	*p*-Value	T−	T+	W	*p*-Value	T−	T+	W
F1	6.90 × 10^−1^	150	315	≈	3.29 × 10^−1^	147	318	≈	8.37 × 10^−2^	84	381	≈
F2	2.49 × 10^−10^	18	447	+	2.87 × 10^−11^	71	394	+	2.87 × 10^−11^	0	465	+
F3	1.66 × 10^−2^	38	427	+	2.87 × 10^−11^	82	383	+	2.87 × 10^−11^	0	465	+
F4	3.11 × 10^−1^	145	319	≈	2.62 × 10^−3^	148	317	+	9.78 × 10^−2^	119	346	≈
F5	1.34 × 10^−3^	103	362	+	2.87 × 10^−11^	0	465	+	1.06 × 10^−8^	2	463	+
F6	2.51 × 10^−5^	23	442	+	1.56 × 10^−3^	188	227	+	2.87 × 10^−11^	0	465	+
F7	5.23 × 10^−11^	0	465	+	4.88 × 10^−8^	15	450	+	2.87 × 10^−11^	0	465	+
F8	2.87 × 10^−11^	0	465	+	6.10 × 10^−3^	96.5	365.5	+	2.87 × 10^−11^	0	465	+
F9	3.08 × 10^−8^	8	457	+	2.94 × 10^−1^	170	295	≈	9.44 × 10^−11^	0	465	+
F10	3.01 × 10^−10^	463	2	-	3.18 × 10^−11^	465	0	-	5.41 × 10^−4^	383	82	-
F11	1.69 × 10^−1^	157	308	≈	1.10 × 10^−1^	175	290	≈	3.64 × 10^−10^	6	459	+
F12	7.34 × 10^−1^	253	212	≈	5.10 × 10^−2^	129	336	≈	2.87 × 10^−11^	0	465	+
F13	3.51 × 10^−11^	0	465	+	1.07 × 10^−4^	42	423	+	2.87 × 10^−11^	0	465	+
F14	8.37 × 10^−2^	103	362	≈	3.59 × 10^−3^	364	101	-	5.66 × 10^−6^	29	436	+
F15	1.04 × 10^−10^	0	465	+	4.37 × 10^−9^	3	462	+	2.87 × 10^−11^	0	465	+
F16	2.58 × 10^−6^	214	251	+	1.12 × 10^−9^	404	61	-	2.58 × 10^−6^	0	465	+
F17	2.51 × 10^−5^	38	427	+	2.87 × 10^−11^	195	270	+	3.88 × 10^−11^	0	465	+
F18	2.20 × 10^−1^	175	290	≈	2.87 × 10^−11^	181	284	+	2.87 × 10^−11^	0	465	+
F19	3.70 × 10^−6^	23	442	+	9.67 × 10^−3^	92	373	+	7.03 × 10^−11^	0	465	+
F20	5.31 × 10^−8^	14	451	+	3.18 × 10^−11^	185	280	+	2.87 × 10^−11^	0	465	+
F21	2.10 × 10^−8^	19	446	+	2.87 × 10^−11^	161	304	+	7.76 × 10^−11^	0	465	+
F22	1.55 × 10^−6^	37	428	+	7.32 × 10^−7^	231	234	+	7.45 × 10^−3^	175	290	+
F23	2.87 × 10^−11^	0	465	+	2.87 × 10^−11^	0	465	+	2.13 × 10^−9^	0	459	+
F24	2.40 × 10^−6^	32	433	+	5.27 × 10^−6^	129	336	+	2.05 × 10^−10^	5	460	+
F25	6.56 × 10^−5^	165	300	+	2.87 × 10^−11^	0	465	+	3.75 × 10^−1^	152	277	≈
F26	2.87 × 10^−11^	0	465	+	9.27 × 10^−3^	110	355	+	2.87 × 10^−11^	0	465	+
F27	3.70 × 10^−6^	26	439	+	5.82 × 10^−7^	10	455	+	8.56 × 10^−11^	0	465	+
F28	2.87 × 10^−11^	0	465	+	2.87 × 10^−11^	0	465	+	5.32 × 10^−10^	1	464	+
F29	2.87 × 10^−11^	0	465	+	2.87 × 10^−11^	0	465	+	2.87 × 10^−11^	0	465	+
F30	2.87 × 10^−11^	0	465	+	2.87 × 10^−11^	0	465	+	2.87 × 10^−11^	0	465	+

**Table 11:** Comparisons of WSRT for ICOA vs. AOA, SHO, and AVOA (D=10).

Func.	AOA-ICOA				SHO-ICOA				AVOA-ICOA			
	*p*-Value	T−	T+	W	*p*-Value	T−	T+	W	*p*-Value	T−	T+	W
F1	2.87 × 10^−11^	0	465	+	2.87 × 10^−11^	0	465	+	3.01 × 10^−10^	0	465	+
F2	2.87 × 10^−11^	0	465	+	2.87 × 10^−11^	0	465	+	2.87 × 10^−11^	0	465	+
F3	2.87 × 10^−11^	0	465	+	2.87 × 10^−11^	0	465	+	2.87 × 10^−11^	0	465	+
F4	2.87 × 10^−11^	0	465	+	9.31 × 10^−10^	11	454	+	2.46 × 10^−2^	174	290	+
F5	2.87 × 10^−11^	0	465	+	4.37 × 10^−9^	4	461	+	5.43 × 10^−5^	66	399	+
F6	2.87 × 10^−11^	0	465	+	1.49 × 10^−8^	8	457	+	4.75 × 10^−3^	88	377	+
F7	2.87 × 10^−11^	0	465	+	2.87 × 10^−11^	0	465	+	1.05 × 10^−2^	113	352	+
F8	2.87 × 10^−11^	0	465	+	2.87 × 10^−11^	0	465	+	1.27 × 10^−2^	134.5	327.5	+
F9	2.87 × 10^−11^	0	465	+	3.51 × 10^−11^	0	465	+	8.70 × 10^−8^	17	448	+
F10	5.32 × 10^−10^	2	463	+	1.66 × 10^−7^	451	14	-	3.51 × 10^−11^	465	0	-
F11	2.87 × 10^−11^	0	465	+	1.55 × 10^−6^	36	429	+	2.20 × 10^−5^	46	419	+
F12	2.87 × 10^−11^	0	465	+	1.25 × 10^−2^	109	356	+	9.50 × 10^−5^	414	51	-
F13	2.87 × 10^−11^	0	465	+	2.87 × 10^−11^	0	465	+	2.76 × 10^−2^	123	342	+
F14	2.87 × 10^−11^	0	465	+	1.15 × 10^−10^	1	464	+	1.35 × 10^−2^	355	110	-
F15	2.87 × 10^−11^	0	465	+	2.87 × 10^−11^	0	465	+	5.41 × 10^−4^	81	384	+
F16	2.87 × 10^−11^	0	465	+	8.94 × 10^−1^	17	448	≈	2.98 × 10^−6^	157	308	+
F17	2.87 × 10^−11^	0	465	+	2.87 × 10^−11^	0	465	+	3.51 × 10^−11^	0	465	+
F18	2.87 × 10^−11^	0	465	+	2.87 × 10^−11^	0	465	+	1.39 × 10^−10^	1	464	+
F19	2.87 × 10^−11^	0	465	+	2.33 × 10^−9^	1	464	+	6.73 × 10^−4^	78	387	+
F20	2.87 × 10^−11^	0	465	+	2.87 × 10^−11^	0	465	+	3.88 × 10^−11^	1	464	+
F21	2.87 × 10^−11^	0	465	+	2.87 × 10^−11^	0	465	+	1.54 × 10^−10^	0	465	+
F22	2.87 × 10^−11^	0	465	+	1.48 × 10^−3^	116	349	+	1.69 × 10^−1^	202	263	≈
F23	2.87 × 10^−11^	0	465	+	1	0	0	≈	1	0	0	≈
F24	2.87 × 10^−11^	0	465	+	1.11 × 10^−7^	9	456	+	2.60 × 10^−4^	67	398	+
F25	2.87 × 10^−11^	0	465	+	8.24 × 10^−1^	87	102	≈	1	0	0	≈
F26	2.87 × 10^−11^	0	465	+	2.87 × 10^−11^	0	465	+	3.08 × 10^−1^	304	161	≈
F27	2.87 × 10^−11^	0	465	+	1.06 × 10^−8^	12	453	+	3.06 × 10^−5^	78	387	+
F28	2.87 × 10^−11^	0	465	+	2.87 × 10^−11^	0	465	+	1	0	0	≈
F29	2.87 × 10^−11^	0	465	+	2.87 × 10^−11^	0	465	+	1	0	0	≈
F30	2.87 × 10^−11^	0	465	+	5.32 × 10^−10^	0	462	+	1.90 × 10^−3^	0	329	+

**Table 12:** Comparisons of WSRT for ICOA vs. BBOA, EVO, and GJO (D=10).

Func.	BBOA-ICOA				EVO-ICOA				GJO-ICOA			
	*p*-Value	T−	T+	W	*p*-Value	T−	T+	W	*p*-Value	T−	T+	W
F1	2.87 × 10^−11^	0	465	+	2.87 × 10^−11^	0	465	+	2.87 × 10^−11^	0	465	+
F2	2.87 × 10^−11^	0	465	+	2.87 × 10^−11^	0	465	+	2.87 × 10^−11^	0	465	+
F3	2.87 × 10^−11^	0	465	+	2.87 × 10^−11^	0	465	+	2.87 × 10^−11^	0	465	+
F4	2.87 × 10^−11^	0	465	+	1.27 × 10^−10^	1	464	+	2.87 × 10^−11^	0	465	+
F5	2.10 × 10^−8^	31	434	+	5.27 × 10^−6^	42	423	+	2.87 × 10^−11^	0	465	+
F6	2.13 × 10^−9^	12	453	+	4.10 × 10^−4^	80	385	+	9.44 × 10^−11^	0	465	+
F7	2.87 × 10^−11^	0	465	+	2.87 × 10^−11^	0	465	+	2.87 × 10^−11^	0	465	+
F8	2.87 × 10^−11^	0	465	+	2.87 × 10^−11^	0	465	+	2.87 × 10^−11^	0	465	+
F9	1.04 × 10^−10^	0	465	+	2.06 × 10^−5^	42	423	+	2.87 × 10^−11^	0	465	+
F10	1.56 × 10^−3^	381	84	-	2.55 × 10^−9^	458	7	-	9.27 × 10^−4^	363	102	-
F11	1.31 × 10^−7^	3	462	+	8.86 × 10^−9^	20	445	+	2.87 × 10^−11^	0	465	+
F12	3.21 × 10^−8^	16	449	+	4.22 × 10^−5^	35	430	+	2.87 × 10^−11^	0	465	+
F13	2.87 × 10^−11^	0	465	+	2.87 × 10^−11^	0	465	+	2.87 × 10^−11^	0	465	+
F14	2.87 × 10^−11^	0	465	+	3.51 × 10^−11^	1	464	+	2.87 × 10^−11^	0	465	+
F15	2.87 × 10^−11^	0	465	+	2.87 × 10^−11^	0	465	+	2.87 × 10^−11^	0	465	+
F16	3.59 × 10^−1^	19	446	≈	1.21 × 10^−4^	0	465	+	5.77 × 10^−11^	0	465	+
F17	5.23 × 10^−11^	0	465	+	2.87 × 10^−11^	0	465	+	2.87 × 10^−11^	0	465	+
F18	2.87 × 10^−11^	0	465	+	2.87 × 10^−11^	0	465	+	2.87 × 10^−11^	0	465	+
F19	6.04 × 10^−2^	152	313	≈	6.37 × 10^−11^	1	464	+	3.88 × 10^−11^	0	465	+
F20	2.87 × 10^−11^	0	465	+	2.87 × 10^−11^	0	465	+	2.87 × 10^−11^	0	465	+
F21	2.87 × 10^−11^	0	465	+	2.87 × 10^−11^	0	465	+	2.87 × 10^−11^	0	465	+
F22	1.41 × 10^−3^	154	311	+	1.94 × 10^−9^	6	459	+	6.73 × 10^−4^	92	373	+
F23	1	0	0	≈	2.87 × 10^−11^	0	465	+	1	0	0	≈
F24	6.37 × 10^−11^	1	464	+	4.40 × 10^−10^	6	459	+	2.87 × 10^−11^	0	465	+
F25	7.60 × 10^−2^	87	242	≈	2.87 × 10^−11^	0	465	+	1	0	0	≈
F26	2.87 × 10^−11^	0	465	+	3.51 × 10^−11^	0	465	+	2.87 × 10^−11^	0	465	+
F27	2.13 × 10^−9^	5	460	+	1.69 × 10^−10^	0	465	+	2.87 × 10^−11^	0	465	+
F28	2.87 × 10^−11^	0	465	+	2.87 × 10^−11^	0	465	+	2.87 × 10^−11^	0	465	+
F29	2.87 × 10^−11^	0	465	+	2.87 × 10^−11^	0	465	+	2.87 × 10^−11^	0	465	+
F30	2.87 × 10^−11^	0	465	+	2.87 × 10^−11^	0	465	+	2.13 × 10^−9^	0	459	+

**Table 13:** Comparisons of WSRT for ICOA vs. HCO, RUN, and COA (D=10).

Func.	HCO-ICOA				RUN-ICOA				COA-ICOA			
	*p*-Value	T−	T+	W	*p*-Value	T−	T+	W	*p*-Value	T−	T+	W
F1	2.87 × 10^−11^	0	465	+	2.87 × 10^−11^	0	465	+	1.12 × 10^−9^	2	463	+
F2	2.87 × 10^−11^	0	465	+	2.87 × 10^−11^	0	465	+	3.51 × 10^−11^	0	465	+
F3	2.87 × 10^−11^	0	465	+	2.87 × 10^−11^	0	465	+	2.87 × 10^−11^	0	465	+
F4	2.87 × 10^−11^	0	465	+	8.79 × 10^−4^	134	331	+	4.44 × 10^−2^	175	290	+
F5	2.87 × 10^−11^	0	465	+	5.32 × 10^−10^	30	435	+	8.02 × 10^−1^	228	237	≈
F6	4.73 × 10^−11^	1	464	+	2.76 × 10^−4^	77	388	+	1.01 × 10^−2^	104	361	+
F7	2.87 × 10^−11^	0	465	+	2.87 × 10^−11^	0	465	+	1.25 × 10^−2^	102	363	+
F8	2.87 × 10^−11^	0	465	+	3.18 × 10^−11^	0	465	+	1.73 × 10^−2^	110	355	+
F9	1.27 × 10^−10^	0	465	+	2.35 × 10^−5^	68	397	+	1.44 × 10^−6^	20	445	+
F10	5.22 × 10^−9^	1	464	+	4.37 × 10^−9^	455	10	-	6.78 × 10^−7^	435	30	-
F11	2.87 × 10^−11^	0	465	+	1.13 × 10^−5^	37	428	+	6.82 × 10^−3^	107	358	+
F12	2.87 × 10^−11^	0	465	+	3.51 × 10^−11^	0	465	+	6.04 × 10^−4^	79	386	+
F13	3.88 × 10^−11^	0	465	+	9.44 × 10^−11^	2	463	+	8.02 × 10^−8^	13	452	+
F14	3.18 × 10^−11^	0	465	+	1.43 × 10^−1^	147	318	≈	3.33 × 10^−2^	136	329	+
F15	2.87 × 10^−11^	0	465	+	2.87 × 10^−11^	0	465	+	1.02 × 10^−7^	3	462	+
F16	1.54 × 10^−4^	4	461	+	1.05 × 10^−5^	172	293	+	5.31 × 10^−8^	291	174	-
F17	2.87 × 10^−11^	0	465	+	2.87 × 10^−11^	0	465	+	1.69 × 10^−10^	1	464	+
F18	2.87 × 10^−11^	0	465	+	2.87 × 10^−11^	0	465	+	3.71 × 10^−5^	67	398	+
F19	2.87 × 10^−11^	0	465	+	1.12 × 10^−9^	7	458	+	1.84 × 10^−4^	66	399	+
F20	2.87 × 10^−11^	0	465	+	2.87 × 10^−11^	0	465	+	3.18 × 10^−11^	0	465	+
F21	2.87 × 10^−11^	0	465	+	2.87 × 10^−11^	0	465	+	1.35 × 10^−9^	26	439	+
F22	2.87 × 10^−11^	0	465	+	5.28 × 10^−2^	264	201	≈	1.90 × 10^−3^	117	348	+
F23	2.87 × 10^−11^	0	465	+	2.87 × 10^−11^	0	465	+	1	0	0	≈
F24	1.54 × 10^−10^	2	463	+	1.25 × 10^−2^	134	331	+	3.31 × 10^−10^	1	464	+
F25	2.87 × 10^−11^	0	465	+	1.07 × 10^−6^	114	351	+	1	0	0	≈
F26	2.87 × 10^−11^	0	465	+	2.87 × 10^−11^	0	465	+	1.44 × 10^−6^	31	434	+
F27	7.03 × 10^−11^	1	464	+	7.04 × 10^−10^	0	465	+	1.23 × 10^−9^	10	455	+
F28	2.87 × 10^−11^	0	465	+	2.87 × 10^−11^	0	465	+	1	0	0	≈
F29	2.87 × 10^−11^	0	465	+	2.87 × 10^−11^	0	465	+	1	0	0	≈
F30	2.87 × 10^−11^	0	465	+	2.87 × 10^−11^	0	465	+	1	0	0	≈

**Table 14:** Summary of WSRT results of all the algorithms for CEC-2014 (D=10).

**Function Type**	**PSO-ICOA**	**DE-ICOA**	**WOA-ICOA**	**AOA-ICOA**	**SHO-ICOA**	**AVOA-ICOA**
Unimodal	2/1/0	2/1/0	2/1/0	3/0/0	3/0/0	3/0/0
Multimodal	8/4/1	7/3/3	11/1/1	13/0/0	11/1/1	10/0/3
Hybrid	5/1/0	6/0/0	6/0/0	6/0/0	6/0/0	5/1/0
Composition	8/0/0	8/0/0	7/1/0	8/0/0	6/2/0	3/5/0
**Total**	***23/6/1***	***23/4/3***	***26/3/1***	***30/0/0***	***26/3/1***	***21/6/3***
**Function type**	**BBOA-ICOA**	**EVO-ICOA**	**GJO-ICOA**	**HCO-ICOA**	**RUN-ICOA**	**COA-ICOA**
Unimodal	3/0/0	3/0/0	3/0/0	3/0/0	3/0/0	3/0/0
Multimodal	11/1/1	12/0/1	12/0/1	13/0/0	11/1/1	10/1/2
Hybrid	5/1/0	6/0/0	6/0/0	6/0/0	5/1/0	6/0/0
Composition	6/2/0	8/0/0	6/2/0	8/0/0	8/0/0	3/5/0
**Total**	***25/4/1***	***29/0/1***	***27/2/1***	***30/0/0***	***27/2/1***	***22/6/2***

**Table 15:** Comparisons of WSRT for ICOA vs. PSO, DE, and WOA (D=30).

Func.	PSO-ICOA				DE-ICOA				WOA-ICOA			
	*p*-Value	T−	T+	W	*p*-Value	T−	T+	W	*p*-Value	T−	T+	W
F1	1.35 × 10^−2^	80	385	+	2.87 × 10^−11^	181	284	+	8.87 × 10^−3^	0	465	+
F2	4.58 × 10^−6^	85	380	+	2.87 × 10^−11^	145	320	+	4.84 × 10^−10^	188	277	+
F3	8.79 × 10^−4^	186	279	+	2.87 × 10^−11^	42	423	+	4.29 × 10^−11^	0	465	+
F4	3.15 × 10^−1^	12	453	≈	2.87 × 10^−11^	79	386	+	3.70 × 10^−6^	0	465	+
F5	2.87 × 10^−11^	0	465	+	2.87 × 10^−11^	0	465	+	2.95 × 10^−8^	14	451	+
F6	6.04 × 10^−2^	1	464	≈	2.87 × 10^−11^	80	385	+	3.18 × 10^−11^	0	465	+
F7	3.45 × 10^−6^	16	449	+	1.27 × 10^−10^	185	280	+	2.56 × 10^−2^	34	431	+
F8	2.07 × 10^−4^	0	465	+	2.87 × 10^−11^	185	280	+	8.70 × 10^−8^	0	465	+
F9	1.03 × 10^−3^	45	420	+	1.66 × 10^−7^	198	267	+	1.02 × 10^−7^	2	463	+
F10	3.21 × 10^−6^	0	465	+	5.23 × 10^−11^	26	439	+	2.55 × 10^−9^	0	465	+
F11	4.13 × 10^−2^	187	278	+	6.90 × 10^−2^	52	413	≈	4.00 × 10^−9^	0	465	+
F12	8.02 × 10^−1^	228	237	≈	1.81 × 10^−5^	34	431	+	2.87 × 10^−11^	0	465	+
F13	5.10 × 10^−5^	55	410	+	3.96 × 10^−7^	250	215	+	1.27 × 10^−3^	77	388	+
F14	9.29 × 10^−1^	130	335	≈	4.28 × 10^−7^	142	323	+	1.56 × 10^−1^	180	285	≈
F15	2.43 × 10^−1^	3	462	≈	2.11 × 10^−7^	46	419	+	2.87 × 10^−11^	0	465	+
F16	2.19 × 10^−4^	99	366	+	7.45 × 10^−1^	48	417	≈	1.29 × 10^−4^	0	465	+
F17	7.10 × 10^−4^	61	404	+	1.27 × 10^−10^	71	394	+	9.31 × 10^−10^	4	461	+
F18	2.92 × 10^−4^	55	410	+	2.55 × 10^−9^	225	240	+	8.79 × 10^−4^	87	378	+
F19	1.21 × 10^−1^	146	319	≈	9.48 × 10^−2^	321	144	≈	8.02 × 10^−8^	18	447	+
F20	1.27 × 10^−3^	18	447	+	2.87 × 10^−11^	244	221	+	5.79 × 10^−5^	0	465	+
F21	2.37 × 10^−1^	163	302	≈	4.73 × 10^−11^	177	288	+	6.46 × 10^−2^	40	425	≈
F22	9.50 × 10^−5^	15	450	+	5.71 × 10^−9^	168	297	+	2.87 × 10^−11^	0	465	+
F23	2.87 × 10^−11^	0	465	+	2.87 × 10^−11^	0	465	+	2.87 × 10^−11^	0	465	+
F24	2.87 × 10^−11^	0	465	+	1.00 × 10^0^	0	0	≈	1.00 × 10^0^	0	0	≈
F25	2.87 × 10^−11^	0	465	+	2.87 × 10^−11^	0	465	+	2.66 × 10^−2^	0	255	+
F26	6.26 × 10^−1^	11	454	≈	2.13 × 10^−9^	442	23	-	1.63 × 10^−4^	2	463	+
F27	2.87 × 10^−11^	0	465	+	2.87 × 10^−11^	0	465	+	2.87 × 10^−11^	0	465	+
F28	2.87 × 10^−11^	0	465	+	2.87 × 10^−11^	0	465	+	2.87 × 10^−11^	0	465	+
F29	2.87 × 10^−11^	0	465	+	2.87 × 10^−11^	0	465	+	3.21 × 10^−6^	0	420	+
F30	4.73 × 10^−11^	0	465	+	8.58 × 10^−6^	115	350	+	6.37 × 10^−11^	0	465	+

**Table 16:** Comparisons of WSRT for ICOA vs. AOA, SHO, and AVOA (D=30).

Func.	AOA-ICOA				SHO-ICOA				AVOA-ICOA			
	*p*-Value	T−	T+	W	*p*-Value	T−	T+	W	*p*-Value	T−	T+	W
F1	2.87 × 10^−11^	0	465	**+**	2.87 × 10^−11^	0	465	+	2.11 × 10^−2^	122	343	+
F2	2.87 × 10^−11^	0	465	+	2.87 × 10^−11^	0	465	+	1.47 × 10^−2^	122	343	+
F3	2.87 × 10^−11^	0	465	+	2.87 × 10^−11^	0	465	+	3.18 × 10^−11^	0	465	+
F4	2.87 × 10^−11^	0	465	+	2.87 × 10^−11^	0	465	+	8.87 × 10^−3^	63	402	+
F5	2.87 × 10^−11^	0	465	+	3.18 × 10^−11^	0	465	+	3.26 × 10^−3^	77	388	+
F6	2.87 × 10^−11^	0	465	+	2.87 × 10^−11^	0	465	+	1.07 × 10^−4^	54	411	+
F7	2.87 × 10^−11^	0	465	+	2.87 × 10^−11^	0	465	+	5.44 × 10^−3^	162	303	+
F8	2.87 × 10^−11^	0	465	+	2.87 × 10^−11^	0	465	+	2.87 × 10^−2^	110	355	+
F9	2.87 × 10^−11^	0	465	+	2.87 × 10^−11^	0	465	+	2.20 × 10^−5^	53	412	+
F10	2.87 × 10^−11^	0	465	+	2.87 × 10^−11^	0	465	+	1.64 × 10^−3^	94	371	+
F11	2.87 × 10^−11^	0	465	+	4.84 × 10^−10^	5	460	+	6.37 × 10^−4^	84	381	+
F12	2.87 × 10^−11^	0	465	+	1.26 × 10^−8^	10	455	+	1.27 × 10^−10^	463	3	-
F13	2.87 × 10^−11^	0	465	+	2.87 × 10^−11^	0	465	+	1.26 × 10^−8^	444	21	-
F14	2.87 × 10^−11^	0	465	+	2.87 × 10^−11^	0	465	+	2.00 × 10^−3^	86	379	+
F15	2.87 × 10^−11^	0	465	+	2.87 × 10^−11^	0	465	+	1.15 × 10^−10^	465	0	+
F16	2.87 × 10^−11^	0	465	+	2.87 × 10^−11^	0	465	+	4.99 × 10^−7^	36	429	+
F17	2.87 × 10^−11^	0	465	+	2.87 × 10^−11^	0	465	+	2.10 × 10^−8^	11	454	+
F18	2.87 × 10^−11^	0	465	+	1.48 × 10^−9^	2	463	+	6.98 × 10^−5^	58	407	+
F19	2.87 × 10^−11^	0	465	+	4.00 × 10^−9^	11	454	+	1.04 × 10^−1^	289	176	≈
F20	2.87 × 10^−11^	0	465	+	2.87 × 10^−11^	0	465	+	5.44 × 10^−3^	95	370	+
F21	2.87 × 10^−11^	0	465	+	2.87 × 10^−11^	0	465	+	3.06 × 10^−5^	20	445	+
F22	2.87 × 10^−11^	0	465	+	2.87 × 10^−11^	0	465	+	1.35 × 10^−2^	102	363	+
F23	2.87 × 10^−11^	0	465	+	5.32 × 10^−10^	0	462	+	1	0	0	≈
F24	2.87 × 10^−11^	0	465	+	1	0	0	≈	1	0	0	≈
F25	2.87 × 10^−11^	0	465	+	1	0	0	≈	1	0	0	≈
F26	2.87 × 10^−11^	0	465	+	2.87 × 10^−11^	0	465	+	1.93 × 10^−6^	430	35	-
F27	2.87 × 10^−11^	0	465	+	2.87 × 10^−11^	0	465	+	1	0	0	≈
F28	2.87 × 10^−11^	0	465	+	1.27 × 10^−10^	0	464	+	1	0	0	≈
F29	2.87 × 10^−11^	0	465	+	6.57 × 10^−1^	0	59	≈	1	0	0	≈
F30	2.87 × 10^−11^	0	465	+	2.87 × 10^−11^	0	465	+	3.83 × 10^−6^	96	368	+

**Table 17:** Comparisons of WSRT for ICOA vs. BBOA, EVO, and GJO (D=30).

Func.	BBOA-ICOA				EVO-ICOA				GJO-ICOA			
	*p*-Value	T−	T+	W	*p*-Value	T−	T+	W	*p*-Value	T−	T+	W
F1	2.87 × 10^−11^	0	465	+	2.87 × 10^−11^	0	465	+	2.87 × 10^−11^	0	465	+
F2	2.87 × 10^−11^	0	465	+	2.87 × 10^−11^	0	465	+	2.87 × 10^−11^	0	465	+
F3	2.87 × 10^−11^	0	465	+	2.87 × 10^−11^	0	465	+	2.87 × 10^−11^	0	465	+
F4	2.87 × 10^−11^	0	465	+	2.87 × 10^−11^	0	465	+	2.87 × 10^−11^	0	465	+
F5	2.87 × 10^−11^	0	465	+	1.80 × 10^−7^	52	413	+	2.87 × 10^−11^	0	465	+
F6	2.87 × 10^−11^	0	465	+	4.73 × 10^−11^	0	465	+	2.87 × 10^−11^	0	465	+
F7	2.87 × 10^−11^	0	465	+	2.87 × 10^−11^	0	465	+	2.87 × 10^−11^	0	465	+
F8	2.87 × 10^−11^	0	465	+	3.51 × 10^−11^	0	465	+	2.87 × 10^−11^	0	465	+
F9	2.87 × 10^−11^	0	465	+	5.10 × 10^−5^	63	402	+	2.87 × 10^−11^	0	465	+
F10	2.87 × 10^−11^	0	465	+	2.87 × 10^−11^	0	465	+	2.87 × 10^−11^	0	465	+
F11	2.87 × 10^−11^	0	465	+	6.81 × 10^−9^	7	458	+	2.87 × 10^−11^	0	465	+
F12	1.26 × 10^−8^	14	451	+	7.32 × 10^−7^	15	450	+	2.87 × 10^−11^	0	465	+
F13	2.87 × 10^−11^	0	465	+	7.03 × 10^−11^	1	464	+	2.87 × 10^−11^	0	465	+
F14	2.87 × 10^−11^	0	465	+	2.87 × 10^−11^	0	465	+	2.87 × 10^−11^	0	465	+
F15	2.87 × 10^−11^	0	465	+	2.87 × 10^−11^	0	465	+	2.87 × 10^−11^	0	465	+
F16	2.87 × 10^−11^	0	465	+	2.87 × 10^−11^	0	465	+	2.87 × 10^−11^	0	465	+
F17	2.87 × 10^−11^	0	465	+	2.87 × 10^−11^	0	465	+	2.87 × 10^−11^	0	465	+
F18	2.87 × 10^−11^	0	465	+	2.87 × 10^−11^	0	465	+	2.87 × 10^−11^	0	465	+
F19	3.51 × 10^−11^	0	465	+	2.87 × 10^−11^	0	465	+	2.87 × 10^−11^	0	465	+
F20	2.87 × 10^−11^	0	465	+	2.87 × 10^−11^	0	465	+	2.87 × 10^−11^	0	465	+
F21	2.87 × 10^−11^	0	465	+	2.87 × 10^−11^	0	465	+	2.87 × 10^−11^	0	465	+
F22	2.87 × 10^−11^	0	465	+	2.87 × 10^−11^	0	465	+	2.87 × 10^−11^	0	465	+
F23	3.39 × 10^−7^	0	437	+	2.87 × 10^−11^	0	465	+	2.13 × 10^−9^	0	459	+
F24	1	0	0	≈	2.87 × 10^−11^	0	465	+	1	0	0	≈
F25	1	0	0	≈	2.87 × 10^−11^	0	465	+	1	0	0	≈
F26	2.87 × 10^−11^	0	465	+	2.87 × 10^−11^	0	465	+	2.87 × 10^−11^	0	465	+
F27	2.87 × 10^−11^	0	465	+	2.87 × 10^−11^	0	465	+	2.87 × 10^−11^	0	465	+
F28	2.87 × 10^−11^	0	465	+	2.87 × 10^−11^	0	465	+	3.75 × 10^−1^	0	114	≈
F29	3.75 × 10^−1^	0	114	≈	2.87 × 10^−11^	0	465	+	8.12 × 10^−9^	0	455	+
F30	2.87 × 10^−11^	0	465	+	2.87 × 10^−11^	0	465	+	2.87 × 10^−11^	0	465	+

**Table 18:** Comparisons of WSRT for ICOA vs. HCO, RUN, and COA (D=30).

Func.	HCO-ICOA				RUN-ICOA				COA-ICOA			
	*p*-Value	T−	T+	W	*p*-Value	T−	T+	W	*p*-Value	T−	T+	W
F1	2.87 × 10^−11^	0	465	+	2.87 × 10^−11^	0	465	+	7.49 × 10^−4^	56	409	+
F2	2.87 × 10^−11^	0	465	+	2.87 × 10^−11^	0	465	+	6.52 × 10^−3^	0	465	+
F3	2.87 × 10^−11^	0	465	+	2.87 × 10^−11^	0	465	+	2.87 × 10^−11^	0	465	+
F4	2.87 × 10^−11^	0	465	+	2.87 × 10^−11^	0	465	+	4.34 × 10^−4^	88	377	+
F5	2.87 × 10^−11^	0	465	+	2.87 × 10^−11^	0	465	+	3.21 × 10^−8^	28	437	+
F6	2.87 × 10^−11^	0	465	+	3.18 × 10^−11^	0	465	+	1.06 × 10^−8^	5	460	+
F7	2.87 × 10^−11^	0	465	+	2.87 × 10^−11^	0	465	+	6.51 × 10^−6^	26	439	+
F8	2.87 × 10^−11^	0	465	+	2.87 × 10^−11^	0	465	+	3.88 × 10^−11^	0	465	+
F9	2.87 × 10^−11^	0	465	+	3.51 × 10^−11^	0	465	+	1.12 × 10^−9^	3	462	+
F10	2.87 × 10^−11^	0	465	+	2.87 × 10^−11^	0	465	+	6.80 × 10^−8^	9	456	+
F11	2.87 × 10^−11^	0	465	+	2.87 × 10^−11^	0	465	+	7.04 × 10^−10^	0	465	+
F12	2.87 × 10^−11^	0	465	+	2.87 × 10^−11^	0	465	+	5.39 × 10^−7^	11	454	+
F13	2.87 × 10^−11^	0	465	+	2.71 × 10^−8^	7	458	+	5.54 × 10^−1^	237	228	≈
F14	2.87 × 10^−11^	0	465	+	3.88 × 10^−11^	0	465	+	1.35 × 10^−2^	116	349	+
F15	2.87 × 10^−11^	0	465	+	2.87 × 10^−11^	0	465	+	6.37 × 10^−4^	65	400	+
F16	2.87 × 10^−11^	0	465	+	2.87 × 10^−11^	0	465	+	2.09 × 10^−1^	160	305	≈
F17	2.87 × 10^−11^	0	465	+	2.87 × 10^−11^	0	465	+	1.63 × 10^−8^	12	453	+
F18	2.87 × 10^−11^	0	465	+	2.87 × 10^−11^	0	465	+	9.85 × 10^−6^	39	426	+
F19	2.87 × 10^−11^	0	465	+	2.87 × 10^−11^	0	465	+	7.01 × 10^−1^	247	218	≈
F20	2.87 × 10^−11^	0	465	+	2.87 × 10^−11^	0	465	+	3.96 × 10^−7^	8	457	+
F21	2.87 × 10^−11^	0	465	+	2.87 × 10^−11^	0	465	+	7.89 × 10^−7^	28	437	+
F22	2.87 × 10^−11^	0	465	+	2.87 × 10^−11^	0	465	+	3.27 × 10^−4^	49	416	+
F23	2.87 × 10^−11^	0	465	+	2.87 × 10^−11^	0	465	+	1	0	0	≈
F24	2.87 × 10^−11^	0	465	+	2.87 × 10^−11^	0	465	+	1	0	0	≈
F25	2.87 × 10^−11^	0	465	+	2.87 × 10^−11^	0	465	+	1	0	0	≈
F26	2.87 × 10^−11^	0	465	+	9.31 × 10^−10^	10	455	+	6.51 × 10^−6^	24	441	+
F27	2.87 × 10^−11^	0	465	+	2.87 × 10^−11^	0	465	+	1	0	0	≈
F28	2.87 × 10^−11^	0	465	+	2.87 × 10^−11^	0	465	+	1	0	0	≈
F29	2.87 × 10^−11^	0	465	+	2.87 × 10^−11^	0	465	+	1	0	0	≈
F30	2.87 × 10^−11^	0	465	+	2.87 × 10^−11^	0	465	+	9.47 × 10^−1^	131	103	≈

**Table 19:** Summary of WSRT results of all the algorithms for CEC-2014 (D=30).

**Function Type**	**PSO-ICOA**	**DE-ICOA**	**WOA-ICOA**	**AOA-ICOA**	**SHO-ICOA**	**AVOA-ICOA**
Unimodal	3/0/0	3/0/0	3/0/0	3/0/0	3/0/0	3/0/0
Multimodal	8/5/0	11/2/0	12/1/0	13/0/0	13/0/0	11/0/2
Hybrid	4/2/0	5/1/0	5/1/0	6/0/0	6/0/0	5/1/0
Composition	7/1/0	6/1/0	7/1/0	8/0/0	5/3/0	1/6/1
**Total**	***22/8/0***	***25/4/1***	***27/3/0***	***30/0/0***	***27/3/0***	***20/7/3***
**Function type**	**BBOA-ICOA**	**EVO-ICOA**	**GJO-ICOA**	**HCO-ICOA**	**RUN-ICOA**	**COA-ICOA**
Unimodal	3/0/0	3/0/0	3/0/0	3/0/0	3/0/0	3/0/0
Multimodal	13/0/0	13/0/0	13/0/0	13/0/0	13/0/0	11/2/0
Hybrid	6/0/0	6/0/0	6/0/0	6/0/0	6/0/0	5/1/0
Composition	5/3/0	8/0/0	5/3/0	8/0/0	8/0/0	1/7/0
**Total**	***27/3/0***	***30/0/0***	***27/3/0***	***30/0/0***	***30/0/0***	***20/10/0***

**Table 20:** Comparisons of *t*-test for ICOA vs. competing algorithms (D=10).

Func.	Algorithms											
	PSO	DE	WOA	AOA	SHO	AVOA	BBOA	EVO	GJO	HCO	RUN	COA
F1	4.64 × 10^−2^	4.55 × 10^−2^	3.39 × 10^−2^	7.17 × 10^−10^	5.33 × 10^−12^	7.18 × 10^−10^	4.28 × 10^−5^	1.27 × 10^−8^	6.03 × 10^−19^	2.09 × 10^−10^	4.91 × 10^−8^	1.40 × 10^−6^
F2	3.34 × 10^−2^	4.84 × 10^−2^	7.90 × 10^−5^	5.96 × 10^−22^	1.06 × 10^−4^	6.84 × 10^−5^	2.66 × 10^−9^	1.98 × 10^−8^	1.49 × 10^−19^	1.77 × 10^−16^	1.46 × 10^−9^	1.33 × 10^−3^
F3	3.98 × 10^−2^	2.04 × 10^−2^	4.78 × 10^−6^	1.48 × 10^−4^	7.36 × 10^−11^	1.38 × 10^−9^	2.20 × 10^−9^	3.37 × 10^−5^	7.40 × 10^−16^	8.42 × 10^−2^	5.18 × 10^−12^	4.83 × 10^−2^
F4	3.68 × 10^−2^	7.85 × 10^−4^	6.37 × 10^−2^	4.59 × 10^−11^	3.95 × 10^−7^	2.26 × 10^−2^	4.00 × 10^−8^	1.12 × 10^−8^	3.67 × 10^−9^	4.06 × 10^−12^	1.51 × 10^−2^	3.95 × 10^−2^
F5	6.58 × 10^−3^	4.28 × 10^−18^	2.92 × 10^−4^	4.05 × 10^−24^	1.30 × 10^−3^	1.55 × 10^−3^	5.42 × 10^−1^	1.32 × 10^−4^	2.75 × 10^−19^	4.38 × 10^−16^	7.37 × 10^−1^	6.47 × 10^−1^
F6	4.69 × 10^−6^	2.86 × 10^−2^	4.97 × 10^−19^	2.97 × 10^−23^	6.28 × 10^−9^	1.88 × 10^−3^	3.36 × 10^−9^	1.16 × 10^−3^	3.77 × 10^−15^	7.10 × 10^−13^	6.99 × 10^−4^	4.24 × 10^−3^
F7	1.60 × 10^−6^	1.94 × 10^−7^	2.26 × 10^−9^	3.11 × 10^−19^	3.31 × 10^−5^	1.57 × 10^−2^	4.09 × 10^−8^	6.24 × 10^−9^	2.15 × 10^−16^	1.57 × 10^−11^	3.79 × 10^−22^	6.27 × 10^−3^
F8	5.46 × 10^−12^	1.54 × 10^−3^	5.56 × 10^−13^	5.16 × 10^−28^	7.56 × 10^−17^	4.53 × 10^−2^	2.99 × 10^−18^	6.20 × 10^−14^	2.81 × 10^−29^	2.96 × 10^−16^	2.50 × 10^−14^	7.01 × 10^−3^
F9	1.31 × 10^−7^	1.69 × 10^−2^	1.72 × 10^−13^	7.46 × 10^−29^	1.76 × 10^−13^	3.85 × 10^−7^	1.26 × 10^−12^	5.94 × 10^−6^	2.67 × 10^−23^	7.82 × 10^−9^	2.37 × 10^−4^	4.13 × 10^−7^
F10	3.23 × 10^−13^	6.91 × 10^−15^	3.73 × 10^−3^	1.94 × 10^−9^	7.78 × 10^−9^	2.58 × 10^−14^	1.70 × 10^−3^	2.99 × 10^−11^	2.78 × 10^−3^	4.69 × 10^−10^	9.60 × 10^−10^	7.71 × 10^−7^
F11	3.07 × 10^−2^	2.70 × 10^−2^	6.17 × 10^−11^	2.54 × 10^−25^	1.37 × 10^−5^	4.46 × 10^−5^	2.07 × 10^−9^	2.30 × 10^−8^	2.44 × 10^−17^	8.92 × 10^−21^	1.03 × 10^−5^	8.20 × 10^−3^
F12	6.61 × 10^−1^	4.36 × 10^−2^	6.22 × 10^−16^	1.90 × 10^−17^	5.22 × 10^−3^	1.53 × 10^−4^	5.23 × 10^−8^	1.87 × 10^−5^	1.57 × 10^−14^	4.05 × 10^−16^	3.81 × 10^−16^	6.79 × 10^−4^
F13	2.35 × 10^−10^	6.88 × 10^−6^	2.53 × 10^−13^	7.52 × 10^−21^	9.97 × 10^−10^	2.75 × 10^−2^	2.36 × 10^−8^	1.39 × 10^−14^	1.25 × 10^−22^	8.21 × 10^−8^	1.31 × 10^−12^	1.53 × 10^−6^
F14	2.82 × 10^−2^	4.49 × 10^−3^	1.74 × 10^−4^	5.73 × 10^−23^	4.12 × 10^−3^	1.01 × 10^−2^	1.26 × 10^−8^	1.59 × 10^−6^	5.20 × 10^−17^	1.05 × 10^−10^	1.99 × 10^−1^	2.85 × 10^−2^
F15	6.62 × 10^−8^	1.15 × 10^−8^	1.08 × 10^−4^	1.62 × 10^−5^	5.73 × 10^−7^	1.61 × 10^−3^	7.10 × 10^−2^	8.59 × 10^−2^	8.37 × 10^−9^	3.34 × 10^−6^	3.65 × 10^−19^	1.91 × 10^−8^
F16	3.99 × 10^−1^	1.51 × 10^−4^	2.17 × 10^−13^	3.66 × 10^−21^	8.96 × 10^−7^	4.28 × 10^−2^	7.11 × 10^−7^	8.00 × 10^−12^	3.05 × 10^−17^	2.00 × 10^−9^	1.00 × 10^−1^	3.00 × 10^−2^
F17	5.91 × 10^−3^	1.01 × 10^−2^	1.41 × 10^−8^	7.22 × 10^−4^	2.52 × 10^−12^	7.53 × 10^−10^	1.16 × 10^−5^	8.55 × 10^−2^	2.04 × 10^−8^	1.64 × 10^−7^	1.85 × 10^−11^	6.87 × 10^−5^
F18	4.10 × 10^−2^	4.46 × 10^−2^	3.09 × 10^−7^	2.33 × 10^−5^	2.30 × 10^−23^	4.16 × 10^−5^	1.25 × 10^−11^	1.28 × 10^−1^	1.30 × 10^−49^	1.26 × 10^−6^	2.42 × 10^−10^	6.91 × 10^−3^
F19	1.41 × 10^−5^	2.34 × 10^−3^	6.69 × 10^−4^	5.99 × 10^−3^	1.03 × 10^−12^	1.30 × 10^−3^	7.68 × 10^−2^	2.48 × 10^−1^	1.31 × 10^−1^	8.14 × 10^−6^	1.84 × 10^−9^	7.04 × 10^−4^
F20	4.75 × 10^−3^	1.99 × 10^−2^	2.09 × 10^−6^	6.63 × 10^−2^	1.46 × 10^−13^	1.29 × 10^−6^	1.47 × 10^−6^	3.00 × 10^−1^	6.78 × 10^−2^	2.22 × 10^−2^	9.16 × 10^−12^	2.31 × 10^−4^
F21	5.62 × 10^−5^	1.14 × 10^−2^	1.42 × 10^−3^	2.87 × 10^−5^	3.77 × 10^−22^	1.27 × 10^−6^	1.29 × 10^−5^	5.75 × 10^−3^	4.09 × 10^−6^	2.19 × 10^−7^	9.80 × 10^−12^	2.59 × 10^−5^
F22	1.71 × 10^−3^	8.04 × 10^−3^	4.30 × 10^−1^	1.53 × 10^−1^	3.05 × 10^−2^	4.24 × 10^−2^	5.03 × 10^−1^	3.14 × 10^−1^	9.74 × 10^−4^	4.74 × 10^−2^	1.60 × 10^−2^	3.11 × 10^−2^
F23	1.77 × 10^−42^	4.20 × 10^−41^	4.88 × 10^−16^	2.55 × 10^−20^	1.00 × 10^0^	1.00 × 10^0^	1.00 × 10^0^	7.33 × 10^−30^	1.00 × 10^0^	3.49 × 10^−19^	8.31 × 10^−2^	1.00 × 10^0^
F24	3.39 × 10^−6^	4.40 × 10^−2^	3.29 × 10^−9^	7.78 × 10^−33^	1.62 × 10^−5^	2.59 × 10^−3^	6.15 × 10^−10^	1.30 × 10^−8^	8.90 × 10^−21^	1.05 × 10^−9^	4.19 × 10^−2^	1.67 × 10^−21^
F25	4.53 × 10^−2^	0.00 × 10^0^	3.49 × 10^−1^	8.42 × 10^−23^	1.17 × 10^−1^	1.00 × 10^0^	2.27 × 10^−1^	3.72 × 10^−15^	1.00 × 10^0^	2.19 × 10^−18^	8.11 × 10^−2^	1.00 × 10^0^
F26	1.23 × 10^−10^	4.90 × 10^−3^	2.76 × 10^−17^	5.59 × 10^−9^	2.55 × 10^−1^	1.90 × 10^−1^	6.24 × 10^−8^	1.54 × 10^−7^	3.80 × 10^−14^	1.64 × 10^−11^	7.03 × 10^−18^	1.48 × 10^−3^
F27	1.47 × 10^−7^	3.21 × 10^−10^	3.29 × 10^−16^	2.43 × 10^−9^	5.74 × 10^−9^	2.63 × 10^−4^	1.45 × 10^−10^	1.32 × 10^−16^	9.23 × 10^−14^	3.58 × 10^−15^	9.81 × 10^−13^	1.58 × 10^−7^
F28	2.24 × 10^−17^	4.25 × 10^−26^	5.01 × 10^−16^	0.00 × 10^0^	9.53 × 10^−20^	1.00 × 10^0^	7.57 × 10^−21^	1.76 × 10^−13^	1.30 × 10^−19^	5.96 × 10^−20^	3.01 × 10^−17^	1.00 × 10^0^
F29	1.15 × 10^−31^	8.17 × 10^−33^	3.15 × 10^−9^	1.32 × 10^−24^	3.19 × 10^−14^	1.00 × 10^0^	7.81 × 10^−6^	5.07 × 10^−12^	2.36 × 10^−24^	6.46 × 10^−14^	1.40 × 10^−26^	1.00 × 10^0^
F30	8.43 × 10^−4^	1.39 × 10^−3^	7.11 × 10^−2^	3.45 × 10^−15^	2.85 × 10^−2^	3.07 × 10^−2^	1.01 × 10^−3^	6.02 × 10^−5^	3.13 × 10^−6^	1.80 × 10^−2^	4.08 × 10^−5^	1.00 × 10^0^

**Table 21:** Comparisons of *t*-test for ICOA vs. competing algorithms (D=30).

Func.	Algorithms											
	PSO	DE	WOA	AOA	SHO	AVOA	BBOA	EVO	GJO	HCO	RUN	COA
F1	1.75 × 10^−3^	3.78 × 10^−2^	2.30 × 10^−5^	2.96 × 10^−27^	1.92 × 10^−14^	1.68 × 10^−2^	5.03 × 10^−17^	2.04 × 10^−14^	2.32 × 10^−22^	4.02 × 10^−18^	3.79 × 10^−15^	9.58 × 10^−4^
F2	3.93 × 10^−2^	3.57 × 10^−2^	6.47 × 10^−1^	6.32 × 10^−35^	2.44 × 10^−18^	2.44 × 10^−2^	3.16 × 10^−26^	2.25 × 10^−19^	2.07 × 10^−27^	4.56 × 10^−30^	9.93 × 10^−21^	3.05 × 10^−2^
F3	4.79 × 10^−2^	8.43 × 10^−6^	2.22 × 10^−3^	1.14 × 10^−3^	8.17 × 10^−24^	3.29 × 10^−8^	1.32 × 10^−25^	1.04 × 10^−17^	8.91 × 10^−35^	1.61 × 10^−4^	1.27 × 10^−24^	4.30 × 10^−6^
F4	3.31 × 10^−7^	2.77 × 10^−2^	3.13 × 10^−10^	7.61 × 10^−23^	2.80 × 10^−11^	4.37 × 10^−3^	6.62 × 10^−19^	4.33 × 10^−13^	8.92 × 10^−23^	1.46 × 10^−20^	3.13 × 10^−17^	2.34 × 10^−3^
F5	2.86 × 10^−19^	2.12 × 10^−17^	7.64 × 10^−5^	1.81 × 10^−36^	1.02 × 10^−15^	6.85 × 10^−4^	1.52 × 10^−25^	2.82 × 10^−4^	2.85 × 10^−33^	6.59 × 10^−30^	7.21 × 10^−33^	2.20 × 10^−5^
F6	4.51 × 10^−14^	2.27 × 10^−3^	1.57 × 10^−25^	2.08 × 10^−30^	6.98 × 10^−19^	8.70 × 10^−5^	3.98 × 10^−22^	1.64 × 10^−13^	1.77 × 10^−23^	8.31 × 10^−24^	1.99 × 10^−15^	6.33 × 10^−10^
F7	6.62 × 10^−5^	4.11 × 10^−2^	8.39 × 10^−2^	3.56 × 10^−33^	1.70 × 10^−17^	3.38 × 10^−2^	5.58 × 10^−25^	1.60 × 10^−17^	4.73 × 10^−27^	6.66 × 10^−25^	3.51 × 10^−24^	3.74 × 10^−4^
F8	7.97 × 10^−15^	4.55 × 10^−2^	7.56 × 10^−18^	8.68 × 10^−40^	3.29 × 10^−22^	8.56 × 10^−3^	1.09 × 10^−31^	3.41 × 10^−14^	4.11 × 10^−33^	1.82 × 10^−25^	5.92 × 10^−21^	2.41 × 10^−12^
F9	2.31 × 10^−5^	2.80 × 10^−1^	5.31 × 10^−11^	7.84 × 10^−29^	1.07 × 10^−13^	2.11 × 10^−5^	3.58 × 10^−17^	2.02 × 10^−4^	1.70 × 10^−22^	4.20 × 10^−19^	1.86 × 10^−14^	3.72 × 10^−10^
F10	5.13 × 10^−14^	7.22 × 10^−6^	2.37 × 10^−23^	7.40 × 10^−37^	7.52 × 10^−26^	1.94 × 10^−3^	4.51 × 10^−29^	1.97 × 10^−18^	3.00 × 10^−40^	1.98 × 10^−35^	1.06 × 10^−20^	8.04 × 10^−6^
F11	3.42 × 10^−1^	1.73 × 10^−5^	1.68 × 10^−12^	1.27 × 10^−29^	3.14 × 10^−11^	1.17 × 10^−3^	1.26 × 10^−18^	2.12 × 10^−8^	4.58 × 10^−23^	8.57 × 10^−27^	1.22 × 10^−19^	3.79 × 10^−13^
F12	8.86 × 10^−1^	5.04 × 10^−6^	7.59 × 10^−18^	7.30 × 10^−22^	2.60 × 10^−8^	2.54 × 10^−10^	1.36 × 10^−7^	2.60 × 10^−4^	1.09 × 10^−20^	2.61 × 10^−18^	1.17 × 10^−19^	4.64 × 10^−7^
F13	7.21 × 10^−5^	4.95 × 10^−1^	6.12 × 10^−4^	3.20 × 10^−37^	2.40 × 10^−24^	2.51 × 10^−8^	7.26 × 10^−30^	4.59 × 10^−14^	1.96 × 10^−31^	1.06 × 10^−28^	2.29 × 10^−9^	4.18 × 10^−2^
F14	5.98 × 10^−3^	2.08 × 10^−2^	1.28 × 10^−1^	6.21 × 10^−28^	1.70 × 10^−20^	5.67 × 10^−4^	2.36 × 10^−28^	2.23 × 10^−17^	4.03 × 10^−30^	1.86 × 10^−21^	1.68 × 10^−7^	1.59 × 10^−2^
F15	2.34 × 10^−8^	6.18 × 10^−6^	1.92 × 10^−11^	8.79 × 10^−15^	1.99 × 10^−5^	7.76 × 10^−10^	1.11 × 10^−9^	4.98 × 10^−7^	2.46 × 10^−12^	5.29 × 10^−11^	1.75 × 10^−17^	3.57 × 10^−4^
F16	2.96 × 10^−3^	9.34 × 10^−6^	6.82 × 10^−14^	2.34 × 10^−20^	4.59 × 10^−13^	8.44 × 10^−6^	3.04 × 10^−14^	3.77 × 10^−17^	1.80 × 10^−16^	1.23 × 10^−18^	8.30 × 10^−12^	1.04 × 10^−1^
F17	2.44 × 10^−2^	1.18 × 10^−3^	4.48 × 10^−5^	1.71 × 10^−14^	6.68 × 10^−5^	2.65 × 10^−8^	9.30 × 10^−8^	1.11 × 10^−5^	1.70 × 10^−9^	1.81 × 10^−12^	4.66 × 10^−14^	2.77 × 10^−3^
F18	3.82 × 10^−5^	6.25 × 10^−1^	1.54 × 10^−3^	7.19 × 10^−18^	3.36 × 10^−2^	3.18 × 10^−4^	1.14 × 10^−6^	2.47 × 10^−2^	2.39 × 10^−12^	2.24 × 10^−15^	1.55 × 10^−14^	4.31 × 10^−5^
F19	2.46 × 10^−1^	4.67 × 10^−2^	4.33 × 10^−3^	5.67 × 10^−12^	2.16 × 10^−1^	1.69 × 10^−1^	1.27 × 10^−2^	6.48 × 10^−2^	2.53 × 10^−5^	1.34 × 10^−11^	1.44 × 10^−7^	9.23 × 10^−1^
F20	4.93 × 10^−7^	3.99 × 10^−2^	1.38 × 10^−11^	1.92 × 10^−7^	2.00 × 10^−2^	1.45 × 10^−3^	5.32 × 10^−2^	3.87 × 10^−2^	1.39 × 10^−7^	1.04 × 10^−4^	1.03 × 10^−4^	5.07 × 10^−6^
F21	1.48 × 10^−1^	3.53 × 10^−2^	5.69 × 10^−5^	2.68 × 10^−13^	3.09 × 10^−8^	1.22 × 10^−7^	1.73 × 10^−8^	8.29 × 10^−7^	7.08 × 10^−15^	8.23 × 10^−13^	2.07 × 10^−14^	1.18 × 10^−6^
F22	4.16 × 10^−7^	2.00 × 10^−2^	7.23 × 10^−16^	1.91 × 10^−4^	3.74 × 10^−23^	4.54 × 10^−3^	2.73 × 10^−1^	1.39 × 10^−1^	6.43 × 10^−4^	7.75 × 10^−5^	3.53 × 10^−5^	9.24 × 10^−5^
F23	1.99 × 10^59^	1.00 × 10^0^	9.39 × 10^−21^	2.66 × 10^−35^	1.55 × 10^−18^	1.00 × 10^0^	5.18 × 10^−10^	4.63 × 10^−20^	2.59 × 10^−12^	3.03 × 10^−18^	2.22 × 10^−30^	1.00 × 10^0^
F24	4.06 × 10^−21^	1.00 × 10^0^	1.00 × 10^0^	6.52 × 10^−26^	1.00 × 10^0^	1.00 × 10^0^	1.00 × 10^0^	4.07 × 10^−27^	1.00 × 10^0^	1.27 × 10^−28^	9.39 × 10^−49^	1.00 × 10^0^
F25	6.23 × 10^−11^	5.14 × 10^−33^	3.13 × 10^−3^	2.49 × 10^−24^	1.00 × 10^0^	1.00 × 10^0^	1.00 × 10^0^	8.46 × 10^−22^	1.00 × 10^0^	4.73 × 10^−20^	3.91 × 10^−16^	1.00 × 10^0^
F26	6.31 × 10^−4^	3.37 × 10^−6^	1.10 × 10^−2^	1.35 × 10^−19^	1.44 × 10^−7^	7.30 × 10^−6^	1.01 × 10^−3^	6.45 × 10^−6^	2.12 × 10^−4^	2.54 × 10^−8^	2.13 × 10^−5^	8.19 × 10^−10^
F27	1.72 × 10^−18^	1.46 × 10^−20^	3.41 × 10^−23^	1.70 × 10^−25^	3.02 × 10^−18^	1.00 × 10^0^	1.84 × 10^−33^	1.04 × 10^−29^	3.91 × 10^−25^	8.48 × 10^−24^	4.74 × 10^−19^	1.00 × 10^0^
F28	1.42 × 10^−15^	2.41 × 10^−36^	4.78 × 10^−22^	2.09 × 10^−33^	4.19 × 10^−18^	1.00 × 10^0^	1.02 × 10^−15^	1.63 × 10^−17^	6.90 × 10^−2^	2.26 × 10^−28^	7.13 × 10^−30^	1.00 × 10^0^
F29	3.69 × 10^−2^	2.61 × 10^−2^	4.02 × 10^−3^	2.76 × 10^−20^	2.06 × 10^−1^	1.00 × 10^0^	5.07 × 10^−2^	2.04 × 10^−13^	3.78 × 10^−11^	1.37 × 10^−12^	2.55 × 10^−10^	1.00 × 10^0^
F30	5.35 × 10^−4^	2.49 × 10^−2^	3.44 × 10^−3^	2.34 × 10^−16^	2.36 × 10^−8^	3.85 × 10^−1^	2.73 × 10^−5^	5.45 × 10^−3^	5.24 × 10^−9^	2.60 × 10^−5^	5.36 × 10^−6^	2.35 × 10^−1^

**Table 22:** Summary of *t*-test results of all the algorithms for CEC-2014.

Alg.	D = 10 (+/−/=)	D = 30 (+/−/=)
PSO	23/5/2	260/0/4
DE	24/6/0	24/1/5
WOA	24/2/4	24/2/4
AOA	28/0/2	30/0/0
SHO	26/1/3	24/2/4
AVOA	20/5/5	20/2/8
BBOA	24/0/6	24/1/5
EVO	23/1/6	28/0/2
GJO	25/1/4	25/2/3
HCO	29/0/1	30/0/0
RUN	24/1/5	30/0/0
COA	21/3/6	21/1/8

**Table 23:** Ablation study results: component-wise performance analysis for representative CEC-2014 functions.

Func.	Dim	COA	COA + V	COA + m	ICOA	COA Execute Time	ICOA Execute Time
F1	10	1.39 × 10^4^	1.06 × 10^4^	8.54 × 10^3^	1.44 × 10^3^	67.568	68.926
F4		1.93 × 10^1^	1.83 × 10^1^	1.87 × 10^1^	1.83 × 10^1^	79.734	80.466
F8		5.35 × 10^0^	3.73 × 10^0^	1.93 × 10^0^	1.16 × 10^0^	65.882	66.186
F11		5.19 × 10^2^	4.30 × 10^2^	4.20 × 10^2^	3.61 × 10^2^	124.644	124.972
F17		4.89 × 10^3^	3.50 × 10^3^	1.57 × 10^3^	2.51 × 10^2^	140.897	142.501
F20		6.95 × 10^3^	6.06 × 10^3^	3.92 × 10^2^	2.61 × 10^1^	146.747	146.865
F24		1.98 × 10^2^	1.90 × 10^2^	1.70 × 10^2^	1.21 × 10^2^	164.135	166.922
F27		1.83 × 10^2^	1.74 × 10^2^	8.48 × 10^1^	6.43 × 10^1^	306.342	309.292
F1	30	9.73 × 10^5^	8.21 × 10^5^	7.40 × 10^5^	5.26 × 10^5^	79.918	80.072
F4		8.60 × 10^1^	6.45 × 10^1^	6.43 × 10^1^	5.88 × 10^1^	83.940	85.720
F8		1.13 × 10^2^	2.58 × 10^1^	9.57 × 10^1^	2.47 × 10^1^	78.780	79.044
F11		4.44 × 10^3^	3.96 × 10^3^	3.27 × 10^3^	3.01 × 10^3^	145.862	146.425
F17		1.26 × 10^5^	4.51 × 10^4^	6.15 × 10^4^	4.05 × 10^4^	167.574	170.213
F20		9.23 × 10^4^	5.40 × 10^4^	6.24 × 10^4^	3.50 × 10^4^	184.263	189.231
F24		2.01 × 10^2^	2.01 × 10^2^	2.01 × 10^2^	2.01 × 10^2^	195.695	196.892
F27		2.00 × 10^2^	2.00 × 10^2^	2.00 × 10^2^	2.00 × 10^2^	372.137	374.889

**Table 24:** The results of ICOA and competitor algorithms for F1–F10 (D=500).

Func	Index	PSO	DE	WOA	AOA	SHO	AVOA	BBOA	EVO	GJO	HCO	RUN	COA	ICOA
F1	Ave	1.24 × 10^7^	7.53 × 10^6^	1.88 × 10^7^	8.88 × 10^9^	6.09 × 10^9^	1.49 × 10^7^	3.13 × 10^9^	1.53 × 10^9^	1.61 × 10^9^	1.81 × 10^10^	6.89 × 10^8^	1.06 × 10^7^	***5.99 × 10^6^***
	Std	1.30 × 10^7^	7.93 × 10^6^	1.81 × 10^7^	1.86 × 10^9^	5.12 × 10^9^	1.97 × 10^7^	1.59 × 10^9^	5.74 × 10^8^	1.22 × 10^9^	1.02 × 10^10^	7.16 × 10^8^	1.14 × 10^7^	***5.39 × 10^6^***
F2	Ave	1.35 × 10^4^	9.49 × 10^3^	1.72 × 10^4^	2.35 × 10^11^	2.17 × 10^11^	1.38 × 10^4^	2.06 × 10^11^	1.28 × 10^11^	1.27 × 10^11^	4.26 × 10^11^	4.86 × 10^10^	1.33 × 10^4^	***5.46 × 10^3^***
	Std	2.98 × 10^4^	1.89 × 10^4^	2.72 × 10^4^	8.03 × 10^10^	1.05 × 10^11^	1.32 × 10^4^	1.20 × 10^11^	8.12 × 10^10^	8.10 × 10^10^	2.35 × 10^11^	4.32 × 10^10^	***7.60 × 10^3^***	1.32 × 10^4^
F3	Ave	1.39 × 10^4^	6.17 × 10^3^	1.48 × 10^4^	1.27 × 10^6^	2.40 × 10^5^	8.64 × 10^3^	2.08 × 10^5^	3.51 × 10^5^	2.23 × 10^5^	9.03 × 10^5^	9.66 × 10^4^	5.79 × 10^3^	***4.71 × 10^3^***
	Std	1.94 × 10^4^	8.37 × 10^3^	2.05 × 10^4^	2.62 × 10^5^	1.07 × 10^5^	9.69 × 10^3^	1.28 × 10^5^	3.25 × 10^5^	9.79 × 10^4^	5.67 × 10^5^	1.02 × 10^5^	7.78 × 10^3^	***6.52 × 10^3^***
F4	Ave	4.46 × 10^2^	***2.64 × 10^1^***	6.13 × 10^2^	8.41 × 10^4^	5.40 × 10^4^	2.31 × 10^2^	6.37 × 10^4^	2.15 × 10^4^	2.06 × 10^4^	7.19 × 10^4^	3.71 × 10^3^	1.94 × 10^2^	1.95 × 10^2^
	Std	4.00 × 10^2^	1.56 × 10^2^	4.71 × 10^2^	4.62 × 10^4^	3.44 × 10^4^	1.87 × 10^2^	4.68 × 10^4^	9.06 × 10^3^	1.40 × 10^4^	4.30 × 10^4^	3.70 × 10^3^	***5.88 × 10^1^***	1.50 × 10^2^
F5	Ave	2.04 × 10^1^	2.01 × 10^1^	2.02 × 10^1^	2.14 × 10^1^	2.12 × 10^1^	2.01 × 10^1^	2.11 × 10^1^	2.01 × 10^1^	2.12 × 10^1^	2.12 × 10^1^	2.12 × 10^1^	2.04 × 10^1^	***2.00 × 10^1^***
	Std	2.76 × 10^−2^	3.00 × 10^−2^	2.93 × 10^−2^	2.23 × 10^−2^	1.15 × 10^−1^	8.27 × 10^−2^	2.59 × 10^−1^	5.13 × 10^−2^	1.35 × 10^−1^	1.31 × 10^−1^	1.50 × 10^−1^	1.10 × 10^−1^	***1.68 × 10^−2^***
F6	Ave	8.62 × 10^1^	9.54 × 10^1^	1.18 × 10^2^	1.27 × 10^2^	1.17 × 10^2^	7.47 × 10^1^	1.09 × 10^2^	8.85 × 10^1^	1.02 × 10^2^	1.15 × 10^2^	9.20 × 10^1^	8.05 × 10^1^	***6.40 × 10^1^***
	Std	8.64 × 10^1^	7.25 × 10^1^	8.07 × 10^1^	6.44 × 10^1^	6.08 × 10^1^	***3.26 × 10^1^***	5.90 × 10^1^	4.20 × 10^1^	5.27 × 10^1^	6.26 × 10^1^	5.36 × 10^1^	5.90 × 10^1^	4.03 × 10^1^
F7	Ave	1.11 × 10^−2^	6.50 × 10^−3^	1.55 × 10^−2^	2.19 × 10^3^	2.21 × 10^3^	1.59 × 10^−2^	1.95 × 10^3^	1.25 × 10^3^	1.28 × 10^3^	1.87 × 10^3^	3.86 × 10^2^	2.03 × 10^−2^	***4.92 × 10^−3^***
	Std	4.28 × 10^−3^	3.22 × 10^−3^	5.88 × 10^−3^	9.75 × 10^2^	1.07 × 10^3^	6.96 × 10^−3^	1.18 × 10^3^	8.86 × 10^2^	8.17 × 10^2^	9.53 × 10^2^	3.40 × 10^2^	2.87 × 10^−2^	***1.87 × 10^−3^***
F8	Ave	5.61 × 10^2^	4.24 × 10^2^	4.75 × 10^2^	1.22 × 10^3^	1.03 × 10^3^	***1.85 × 10^2^***	9.21 × 10^2^	5.38 × 10^2^	9.39 × 10^2^	8.80 × 10^2^	7.02 × 10^2^	2.20 × 10^2^	2.19 × 10^2^
	Std	2.23 × 10^2^	1.63 × 10^2^	2.66 × 10^2^	5.67 × 10^2^	5.90 × 10^2^	1.23 × 10^2^	4.79 × 10^2^	4.30 × 10^2^	5.16 × 10^2^	3.69 × 10^2^	4.59 × 10^2^	2.36 × 10^2^	***8.45 × 10^1^***
F9	Ave	6.92 × 10^2^	***4.39 × 10^2^***	1.02 × 10^3^	1.20 × 10^3^	1.11 × 10^3^	4.54 × 10^2^	9.39 × 10^2^	8.17 × 10^2^	9.25 × 10^2^	9.05 × 10^2^	8.68 × 10^2^	4.94 × 10^2^	4.59 × 10^2^
	Std	4.51 × 10^2^	4.92 × 10^2^	1.04 × 10^3^	5.20 × 10^2^	5.68 × 10^2^	2.88 × 10^2^	5.77 × 10^2^	5.48 × 10^2^	4.53 × 10^2^	4.58 × 10^2^	5.40 × 10^2^	***2.34 × 10^2^***	3.31 × 10^2^
F10	Ave	3.78 × 10^3^	3.58 × 10^3^	5.10 × 10^3^	2.57 × 10^4^	2.28 × 10^4^	2.30 × 10^3^	1.95 × 10^4^	1.24 × 10^4^	2.02 × 10^4^	2.32 × 10^4^	1.75 × 10^4^	2.78 × 10^3^	***1.85 × 10^3^***
	Std	3.54 × 10^3^	2.50 × 10^3^	4.48 × 10^3^	1.24 × 10^4^	1.22 × 10^4^	***1.41 × 10^3^***	1.11 × 10^4^	8.39 × 10^3^	1.07 × 10^4^	1.35 × 10^4^	8.34 × 10^3^	2.57 × 10^3^	1.43 × 10^3^

**Table 25:** Statistical comparisons of ICOA and competing algorithms for cantilever beam design.

Alg.	Best	Ave	Worst	Std	Execute Time
ICOA	***1.339965***	***1.340014***	***1.340091***	***3.07 × 10^−5^***	1.27496
COA	1.339974	1.340178	1.341242	2.89 × 10^−4^	1.22471
AOA	1.340798	1.346354	1.358246	3.66 × 10^−3^	1.23660
AVOA	1.339978	1.340164	1.340509	1.47 × 10^−4^	1.63958
BBOA	1.678988	3.647702	6.604720	1.33 × 10^0^	2.24914
EVO	1.590256	2.824445	4.496960	6.31 × 10^−1^	3.69383
GJO	1.340045	1.340440	1.340440	3.02 × 10^−4^	1.27667
HCO	3.512354	6.253555	8.385139	1.28 × 10^0^	0.96254
RUN	1.340820	1.346735	1.352481	2.74 × 10^−3^	3.03150
SHO	1.345395	1.375816	1.445755	2.22 × 10^−2^	1.54913
PSO	1.340031	1.340040	1.340068	7.99 × 10^−6^	1.43468
DE	1.339990	1.340035	1.340112	4.32 × 10^−10^	1.71873
WOA	2.211835	5.16124	8.86818	1.92 × 10^0^	1.15853

**Table 26:** The best results of ICOA and competing algorithms for cantilever beam design.

Alg.	Optimal Variables					Optimal Weigh
	X1	X2	X3	X4	X5	
ICOA	***6.00741***	***5.32034***	***4.49318***	***3.50262***	***2.15025***	***1.339965***
COA	5.99646	5.32403	4.48720	3.50900	2.15725	1.339974
AOA	6.14601	5.32551	4.39422	3.47063	2.15077	1.340798
AVOA	6.00736	5.29199	4.51273	3.50162	2.16030	1.339978
BBOA	5.46754	5.16362	9.12321	2.79622	4.35626	1.678988
EVO	7.18146	6.29623	3.54348	5.50300	2.96070	1.590256
GJO	6.04345	5.27270	4.50876	3.49917	2.15099	1.340045
HCO	20.33664	6.68054	8.63522	2.91363	17.72169	3.512354
RUN	5.92967	5.34792	4.56708	3.42831	2.21453	1.340820
SHO	6.01809	5.41483	4.79057	3.37090	1.96643	1.345395
PSO	6.05320	5.27831	4.50514	3.50346	2.13476	1.340031
DE	6.03635	5.31036	4.49535	3.47560	2.15654	1.339990
WOA	9.55734	9.87126	3.10593	3.01073	9.90082	2.211835

**Table 27:** Statistical comparisons of ICOA and competing algorithms for gear train design.

Alg.	Best	Ave	Worst	Std	Execute Time
ICOA	***2.70086 × 10^−12^***	3.63159 × 10^−9^	***1.18341 × 10^−9^***	5.78387 × 10^−9^	1.19214
COA	***2.70086 × 10^−12^***	5.48541 × 10^−10^	2.35764 × 10^−9^	5.82634 × 10^−10^	1.16125
AOA	2.30782 × 10^−11^	6.62514 × 10^−9^	2.72645 × 10^−8^	9.39338 × 10^−9^	1.13105
AVOA	***2.70086 × 10^−12^***	5.43563 × 10^−10^	2.35764 × 10^−9^	6.08656 × 10^−10^	1.62427
BBOA	1.36165 × 10^−9^	1.90407 × 10^−4^	3.92092 × 10^−3^	7.31225 × 10^−4^	2.30207
EVO	***2.70086 × 10^−12^***	3.98385 × 10^−7^	2.41494 × 10^−6^	6.50550 × 10^−7^	3.71810
GJO	2.30782 × 10^−11^	4.92848 × 10^−10^	2.35764 × 10^−9^	6.32866 × 10^−10^	1.15495
HCO	1.36165 × 10^−9^	4.65817 × 10^−4^	4.81704 × 10^−3^	1.22843 × 10^−3^	0.91430
RUN	***2.70086 × 10^−12^***	***3.05617 × 10^−10^***	***1.18341 × 10^−9^***	***4.22063 × 10^−10^***	2.60598
SHO	9.92158 × 10^−10^	5.95694 × 10^−8^	7.77863 × 10^−7^	1.95440 × 10^−7^	1.50559
PSO	***2.70086 × 10^−12^***	1.92707 × 10^−9^	8.70083 × 10^−9^	2.26206 × 10^−9^	1.37893
DE	***2.70086 × 10^−12^***	4.03119 × 10^−10^	2.35764 × 10^−9^	7.32360 × 10^−10^	1.69266
WOA	***2.70086 × 10^−12^***	5.16815 × 10^−9^	3.88059 × 10^−8^	7.80601 × 10^−9^	1.12738

**Table 28:** The best results of ICOA and competing algorithms for gear train design.

Alg.	Optimal Variables				Optimal Cost
	X1	X2	X3	X4	
ICOA	***43***	***16***	***19***	***49***	***2.70086 × 10^−12^***
COA	43	19	16	49	2.70086 × 10^−12^
AOA	51	30	13	53	2.30782 × 10^−11^
AVOA	43	16	19	49	2.70086 × 10^−12^
BBOA	60	34	14	55	1.36165 × 10^−9^
EVO	54	27	14	48	2.70086 × 10^−12^
GJO	51	15	26	53	2.30782 × 10^−11^
HCO	60	14	34	55	1.36165 × 10^−9^
RUN	43	19	16	49	2.70086 × 10^−12^
SHO	47	12	13	23	9.92158 × 10^−10^
PSO	43	16	19	49	2.70086 × 10^−12^
DE	49	16	19	43	2.70086 × 10^−12^
WOA	49	16	19	43	2.70086 × 10^−12^

**Table 29:** Statistical comparisons of ICOA and competing algorithms for rolling element bearing design.

Alg.	Best	Ave	Worst	Std	Execute Time
ICOA	***85,547.81075***	***85,498.37802***	85,129.74657	110.25947	2.10745
COA	85,417.68346	84,196.95936	76,621.89497	2061.33780	2.07492
AOA	85,498.82923	82,738.60151	80,625.48721	1472.93601	1.87460
AVOA	85,470.17433	85,436.57894	***85,211.57496***	***56.83115***	2.00904
BBOA	67,547.45237	51,280.09397	41,368.89540	7020.17021	2.91190
EVO	78,809.55710	60,138.00288	40,804.90764	9097.12415	4.00890
GJO	85,509.21685	85,072.63773	84,305.32188	346.53160	2.06363
HCO	62,000.33053	40,744.99121	26,775.78264	9688.44748	1.27705
RUN	84,406.39164	78,291.80792	72,219.81683	2999.95596	5.48862
SHO	82,042.76450	66,561.86365	57,683.98071	6263.99900	2.06516
PSO	85,541.56851	84,461.70325	75,074.61692	2225.01216	1.77061
DE	85,488.74415	85,410.85487	85,241.52514	82.15798	2.10831
WOA	84,821.70430	66,373.59830	43,374.08175	9824.43939	1.49705

**Table 30:** The best results of ICOA and competing algorithms for rolling element bearing design.

Alg.	Optimal Variables										Optimal Load-Carrying Capacity
	X1	X2	X3	X4	X5	X6	X7	X8	X9	X10	
ICOA	150.00000	19.60000	14.05500	0.51500	0.51500	0.49467	0.70000	0.37348	0.09826	0.60029	85,547.81075
COA	150.00000	19.59040	14.04385	0.51500	0.54838	0.41610	0.69977	0.31708	0.07042	0.65990	85,417.68346
AOA	150.00000	19.59444	14.05402	0.51500	0.51500	0.40000	0.70000	0.34178	0.02000	0.85000	85,498.82923
AVOA	149.99997	19.60000	14.03587	0.51500	0.51502	0.42607	0.70000	0.37327	0.02522	0.73401	85,470.17433
BBOA	140.15308	17.40978	13.64071	0.51500	0.51500	0.45613	0.64064	0.31034	0.05163	0.79420	67,547.45237
EVO	144.13745	18.80901	13.89104	0.51500	0.51500	0.47843	0.67646	0.38529	0.06847	0.60000	78,809.55710
GJO	150.00000	19.59682	14.05670	0.51500	0.60000	0.43896	0.70000	0.36977	0.02046	0.67765	85,509.21685
HCO	136.45277	17.75661	13.44485	0.52000	0.58243	0.48522	0.64840	0.39257	0.03490	0.64648	62,000.33053
RUN	147.80636	19.53148	13.89983	0.51501	0.56775	0.40217	0.69759	0.30157	0.08539	0.74734	84,406.39164
SHO	148.83557	19.07543	14.22603	0.51500	0.51500	0.40000	0.69609	0.30000	0.02000	0.60000	82,042.76450
PSO	149.99732	19.59998	14.05478	0.51500	0.54025	0.48633	0.70000	0.31846	0.06652	0.74275	85,541.56851
DE	150.00000	19.59970	14.04378	0.51500	0.56489	0.50000	0.70000	0.33171	0.03925	0.61213	85,488.74415
WOA	149.95849	19.48097	14.11789	0.51500	0.59783	0.44713	0.69981	0.30000	0.06881	0.80023	84,821.70430

**Table 31:** Statistical comparisons of ICOA and competing algorithms for heat exchanger network design.

Alg.	Best	Ave	Worst	Std	Execute Time
ICOA	***7083.33171***	***7083.33171***	***7083.33171***	***6.89 × 10^−11^***	1.68429
COA	7083.33173	7083.43778	7083.86566	1.34 × 10^−1^	1.65863
AOA	7083.39035	10,497.48515	14,636.75742	2.43 × 10^3^	1.47541
AVOA	7083.39657	9457.66839	13,607.05720	1.97 × 10^3^	1.74121
BBOA	7083.34356	9996.10612	9996.10612	2.02 × 10^3^	2.41270
EVO	3.010 × 10^18^	1.392 × 10^20^	4.958 × 10^20^	1.26 × 10^20^	3.81911
GJO	7084.06861	9586.58148	13,075.74652	2.09 × 10^3^	1.64325
HCO	1.01 × 10^20^	8.45 × 10^20^	1.53 × 10^21^	3.69 × 10^20^	1.02708
RUN	5.02 × 10^14^	4.36 × 10^16^	1.47 × 10^17^	4.09 × 10^16^	4.40927
SHO	7085.31046	10,740.24843	13,803.23418	1.82 × 10^3^	1.70422
PSO	7278.55164	38,496.92404	657,571.58608	1.19 × 10^5^	1.53752
DE	7083.33172	7083.33173	7083.33174	7.75 × 10^−6^	1.83050
WOA	7084.11786	7084.18510	7084.33171	7.42 × 10^−2^	1.24512

**Table 32:** The best results of ICOA and competing algorithms for heat exchanger network design.

Alg.	Optimal Variables								Optimal Weight
	X1	X2	X3	X4	X5	X6	X7	X8	
ICOA	833.33171	1000.00000	5250.00000	200.00000	226.52015	200.00000	208.14713	313.50735	7083.33171
COA	833.33173	1000.00000	5250.00000	200.00000	342.14339	200.00000	213.24883	372.45671	7083.33173
AOA	833.39657	1000.00000	5250.00000	200.00000	312.80710	200.00000	200.00000	300.85189	7083.39035
AVOA	6401.97024	1955.08696	5250.00000	200.00000	255.36994	200.00000	285.03443	300.00000	7083.39657
BBOA	833.34356	1000.00000	5250.00000	200.00000	309.78461	200.00000	201.20677	300.26021	7083.34356
EVO	2323.70447	4196.41566	8010.73251	215.17004	368.72519	206.77365	209.83636	466.21007	3.01 × 10^18^
GJO	834.06861	1000.00000	5250.00000	200.00000	334.32038	200.00000	214.57454	362.41009	7084.06861
HCO	7514.54982	7008.70382	7296.34686	207.76339	329.57803	319.18097	260.86927	371.01401	1.01 × 10^20^
RUN	8229.17631	4692.25921	9044.09747	200.13973	299.25466	200.14378	208.87721	391.53264	5.02 × 10^14^
SHO	835.31046	1000.00000	5250.00000	200.00000	240.61885	200.00000	267.08589	310.30210	7085.31046
PSO	841.34890	1035.19770	5385.30518	200.00000	255.61261	200.00000	206.74049	312.88869	7278.55164
DE	834.11786	1000.00000	5250.00000	200.00000	238.24044	200.00000	331.58420	330.08496	7083.33172
WOA	834.11786	1000.00000	5250.00000	200.00000	238.24044	200.00000	331.58420	330.08496	7084.11786

**Table 33:** Statistical comparisons of ICOA and competing algorithms for tabular column design.

Alg.	Best	Ave	Worst	Std	Execute Time
ICOA	***26.53132788***	***26.531344***	26.532119	4.65 × 10^−5^	1.11318
COA	26.53133097	26.531420	26.531734	8.46 × 10^−5^	1.10274
AOA	26.85384345	27.742752	28.913339	4.93 × 10^−1^	1.09600
AVOA	26.53132791	26.531352	***26.531446***	***3.26 × 10^−5^***	1.73041
BBOA	26.78579561	28.541879	31.572845	1.34 × 10^0^	2.42526
EVO	26.55358141	27.773353	31.533105	1.03 × 10^0^	3.70978
GJO	26.53456288	26.543037	26.553968	5.42 × 10^−3^	1.09865
HCO	26.64907321	29.219263	33.194887	1.47 × 10^0^	1.10662
RUN	26.53470498	26.552540	26.603866	1.61 × 10^−2^	1.90228
SHO	26.53214517	26.701739	27.305270	1.94 × 10^−1^	1.66486
PSO	26.53448094	26.540042	26.550116	5.94 × 10^−3^	1.55243
DE	26.53132922	26.534228	26.540988	4.50 × 10^−3^	1.81912
WOA	26.58234046	28.255892	33.192385	1.61 × 10^0^	1.25096

**Table 34:** The best results of ICOA and competing algorithms for tabular column design.

Alg.	Optimal Variables		Optimum Cost
	X1	X2	
ICOA	5.45116	0.29197	26.53132788
COA	5.45116	0.29197	26.53133097
AOA	5.36765	0.30579	26.85384345
AVOA	5.45116	0.29197	26.53132791
BBOA	5.38409	0.30295	26.78579561
EVO	5.44605	0.29285	26.55358141
GJO	5.45068	0.29207	26.53456288
HCO	6.23869	0.33817	26.64907321
RUN	5.45159	0.29199	26.53470498
SHO	5.45094	0.29200	26.53214517
PSO	5.45081	0.29206	26.53448094
DE	5.45116	0.29197	26.53132922
WOA	5.43733	0.29418	26.58234046

**Table 35:** Keywords of ROAS problem.

Keywords	Variable
Makarna lütfen	x
Pasta	y
Toddler food	z
Organic	a
Baby biscuit	b
Biscuit	c
Baby semolina	d
Baby tarhana	e
Baby butter	f
Rice flour	g
Baby curd	h
Gluten-free pasta	p
Pudding	w

**Table 36:** The results of ICOA and competitor algorithms for ROAS problems.

	ICOA	COA	APO	FLA	GGO	HO	HOA	CFOA	EEFO	PO
Σx	28,000.04	26,000.09	27,768.23	27,998.53	26,020.49	26,091.9	26,040.57	27,868.94	26,136.68	24,001.39
Σy	9099.80	9118.60	9000.69	9082.79	9010.61	9010.61	9002.57	9096.33	9046	9013
Σz	6350.60	6085.88	6353.97	6072.36	6031.64	6009.1	6070.76	6160.19	6047.69	6305.72
Σa	4800	4731.69	4752.05	4737.15	4780.59	4758.67	4753.43	4732.73	4754.47	4726.49
Σb	500	256.14	250	263.9	254.64	251.22	250	317.68	308.92	250
Σc	1125	1124.32	1021.3	1137.41	900.26	1131.82	1120.88	904.98	1141.51	1048.93
Σd	6900.1	6905.52	6891.82	6913.15	6850.4	6865.08	6857.95	6907.49	6870.19	6857.59
Σe	2760	2807.87	2761.33	2763.39	2768.46	2760.07	2760.52	2798.87	2760	2796.4
Σf	5600.02	5597.47	5609.19	5607.62	5599.27	5631.74	5597.69	5599.35	5600.96	5592.83
Σg	213.66	202.38	214.93	200.79	204.925	200.07	200.68	202.07	202.36	200.71
Σh	110.11	54.89	63.55	116.68	51.6	50.41	50.8	82.68	50	50.87
Σp	70	70.09	70.62	70	70.48	70.2	70.02	76.95	75.79	70.95
Σw	700	700.04	700.04	700	700.02	700	700	700	700.06	700
Total	66,229.34	63,654.97	65,457.7	65,663.75	63,243.39	63,530.87	63,475.85	65,448.2	63,694.61	61,614.88
ROAS	***144.601***	144.072	143.353	144.211	142.694	143.202	143.838	143.15	143.939	142.473

## Data Availability

The raw data supporting the conclusions of this article will be made available by the authors on request.

## Nomenclature

temp	Temperature
p	Crayfish intake
σ, $C_{1}$ and $C_{2}$	Control parameter
μ	The best temperature for crayfish
t	Iteration
T	Maximum iteration
$X_{shade}$	Cave location
$X_{G}$	Best location
$X_{L}$	Best location (current population)
$X_{i,j}^{t}$	The position of the ith agent in the jth dimension at the tth iteration
$X_{i,j}^{t+1}$	The position of the ith agent in the jth dimension at the (t + 1)th iteration
$X_{z,j}^{t}$	The position of the randomly selected zth agent from the population in the jth dimension at the tth iteration
N	Population size
z	Randomly selected agent
$C_{3}$	food factor
$X_{food}$	location of food
${fitness}_{i}$	Fitness value of agent i
${fitness}_{food}$	Fitness value of the food
V	step length
m	mapping vector
d, D	Dimension of the problem
MHA	Meta-heuristic algorithms
FE	Function evaluations
WSRT	Wilcoxon Signed-Rank Test
PSO	Particle Swarm Optimization
DE	Differential Evolution
WOA	Whale Optimization Algorithm
AOA	Arithmetic Optimization Algorithm
AVOA	African Vultures Optimization Algorithm
GJO	Golden Jackal Optimization
SHO	Sea-Horse Optimizer
EVO	Energy Valley Optimizer
HCO	Human Conception Optimizer
RUN	RUNge Kutta optimizer
BBOA	Brown-Bear Optimization Algorithm
COA	Crayfish Optimization Algorithm
ICOA	Improved Crayfish Optimization Algorithm
ROAS	Return on advertising spend
AS	Advertising spend
R	Revenue
APO	Artificial Protozoa Optimizer
FLA	Flood Algorithm
GGO	Greylag Goose Optimization
HO	Hippopotamus Optimization
HOA	Hiking Optimization Algorithm
CFOA	Catch Fish Optimization Algorithm
EEFO	Electric Eel Foraging Optimization
PO	Puma optimizer

## Appendix A

**Table A1:** Group 1 keywords information.

Keywords	Advertising Spend	Num. of Impressions	Num. of Clicks	Hit Rate(%)	Sales Volume	Revenue	ROAS	Proposed CPM	Actual CPM	Selected CPM
Makarna lutfen	1299.50	9532	156	1.64	130	18,940.01	14.57	42.51	136.33	225.42
Baby biscuit	462.85	8165	58	0.71	8	1277.16	2.76	18.41	56.69	206.04
Biscuit	16.56	4523	14	0.31	32	5251.80	317.14	15.22	3.66	145.51
Organic	116.36	1742	17	0.98	3	312.59	2.69	18.43	66.80	211.30
Pasta	402.09	6377	18	0.28	1	105.61	0.26	64.72	63.05	86.76
Toddler food	37.40	830	37	4.46	13	1667.82	44.59	21.00	45.06	101.03
Baby semolina	93.94	1180	33	2.80	21	2623.33	27.93	32.65	79.61	87.34
Baby tarhana	51.98	832	9	1.08	3	434.52	8.36	37.28	62.48	102.37
Gluten-free pasta	43.91	1207	9	0.75	6	612.40	13.95	12.37	36.38	53.18

**Table A2:** Group 2 keyword information.

Keywords	Advertising Spend	Num. of Impressions	Num. of Clicks	Hit Rate (%)	Sales Volume	Revenue	ROAS	Proposed CPM	Actual CPM	Selected CPM
Makarna lutfen	442.97	7497	83	1.11	92	13,709.16	30.95	73.57	59.09	124.99
Baby semolina	118.11	1905	45	2.36	9	1538.10	13.02	28.05	62.00	103.69
Pasta	148.75	3353	12	0.36	5	929.50	6.25	30.48	44.36	76.92
Baby tarhana	50.68	829	36	4.34	6	746.40	14.73	41.18	61.13	67.88
Gluten-free pasta	51.18	2177	51	2.34	5	822.50	16.07	13.30	23.51	45.05
Pudding	2.36	933	3	0.32	3	459.70	194.79	10.28	2.53	50.28
Toddler food	46.34	748	10	1.34	2	309.80	6.69	45.59	61.95	97.84
Organic	0.25	132	3	2.27	10	1405.63	5622.52	12.37	1.89	52.37

**Table A3:** Group 3 keyword information.

Keywords	Advertising Spend	Num. of Impressions	Num. of Clicks	Hit Rate (%)	Sales Volume	Revenue	ROAS	Proposed CPM	Actual CPM	Selected CPM
Pudding	1076.37	27,121	145	0.53	4	1106.6	1.03	14.46	39.69	74.4
Makarna lutfen	10,331.77	94,109	10,065	10.7	10,703	1,211,873.38	117.3	23.4	109.79	138
Organic	131.63	5060	87	1.72	6	599.19	4.55	38.69	26.01	72
Pasta	906.84	7287	31	0.43	10	661	0.73	28.17	124.45	145.71
Baby curd	9.86	242	9	3.72	13	1952.44	198.02	14.24	40.74	57
Baby butter	2.11	110	9	8.18	4	999.78	473.83	12.37	19.18	21
Toddler food	1449.46	15,005	252	1.68	93	11,648.8	8.04	40.98	96.6	541

**Table A4:** Group 4 keyword information.

Keywords	Advertising Spend	Num. of Impressions	Num. of Clicks	Hit Rate (%)	Sales Volume	Revenue	ROAS	Proposed CPM	Actual CPM	Selected CPM
Makarna lutfen	661.88	9722	83	0.85	138	20,105.91	30.38	63.96	68.08	74.65
Toddler food	206.31	2750	18	0.65	8	997.61	4.84	48.15	75.02	112.41
Pasta	404.93	5280	22	0.42	1	319.9	0.79	25.69	76.69	104.72
Organic	6.83	200	4	2	3	541.7	79.31	11.06	34.15	40.79
Biscuit	1115.59	13,124	30	0.23	8	1447.2	1.3	49.36	85	86.39
Baby biscuit	36.96	708	8	1.13	4	1134.6	30.7	38.62	52.2	102.37
Baby semolina	1566.92	19,175	509	2.65	189	21,785.87	13.9	25.14	81.72	163.4

**Table A5:** Group 5 keyword information.

Keywords	Advertising Spend	Num. of Impressions	Num. of Clicks	Hit Rate (%)	Sales Volume	Revenue	ROAS	Proposed CPM	Actual CPM	Selected CPM
Makarna lutfen	17,665.35	11,4206	13,709	12	13,790	1,811,070.41	102.52	81.57	154.68	287
Pasta	7494.32	56,982	638	1.12	371	53,609.57	7.15	70.41	131.52	212
Toddler food	1784.95	12,867	349	2.71	111	14,323.48	8.02	41.9	138.72	370
Baby semolina	5198.98	37,666	955	2.54	342	38,720.82	7.45	25.08	138.03	910
Baby tarhana	2780.55	16,440	381	2.32	131	16,951.23	6.1	27.39	169.13	291
Organic	4511.94	17,223	337	1.96	26	3479.74	0.77	57.02	261.97	112
Baby butter	4389.9	60,975	308	0.51	27	3451.92	0.79	67.66	72	170
Rice flour	107.78	2770	24	0.87	10	1773	16.45	16.71	38.91	93.26

**Table A6:** Group 6 keyword information.

Keywords	Advertising Spend	Num of Impressions	Num. of Clicks	Hit Rate (%)	Sales Volume	Revenue	ROAS	Proposed CPM	Actual CPM	Selected CPM
Makarna lutfen	300.51	6952	53	0.76	54	9519.31	31.68	54.93	43.23	65.21
Rice flour	183.78	6480	36	0.56	5	859.5	4.68	13.62	28.36	52.37
Organic	36.74	796	27	3.39	8	1515.2	41.24	12.37	46.16	61.02
Pasta	12.98	1311	12	0.92	6	931.4	71.76	20.57	9.9	57.29
Toddler food	3292.64	33,583	620	1.85	139	16,264.6	4.94	58.08	98.04	300
Baby semolina	9.83	253	36	14.23	20	2279	231.84	45.55	38.85	45
Baby butter	1655.21	79,968	583	0.73	28	5149.68	3.11	12.99	20.7	28
Baby curd	74.21	5589	60	1.07	18	2779.6	37.46	12.37	13.28	46
